# Supplementary material for: Novel Zebrafish Mono-α2,8-sialyltransferase (ST8Sia VIII): An Evolutionary Perspective of α2,8-Sialylation
Source: Int J Mol Sci. 2019 Jan 31;20(3):622. doi: 10.3390/ijms20030622 (PMC6387029; doi:10.3390/ijms20030622)
Supplement: Supplementary file 1 [file ijms-20-00622-s001.pdf]

Novel zebrafish mono-alpha2,8-sialyltransferase (ST8Sia VIII): an evolutionary perspective of alpha2,8-sialylation.

Lan Yi Chang[1], Roxana Elin Teppa[2], Maxence Noel[1], Pierre-André Gilormini[1], Mathieu Decloquement[1], Christine Thisse[2], Bernard Thisse[2], Anne Marie Mir[1], Virginie Cogez[1], Philippe Delannoy[1], Kay Hooi Khoo[4], Daniel Petit[5], Yann Guérardel[1], Anne Harduin-Lepers[1‡]

[1]Université de Lille, CNRS, UMR 8576 - UGSF - Unité de Glycobiologie Structurale et Fonctionnelle, F-59000 Lille, France

[2]Bioinformatics Unit, Fundación Instituto Leloir-IIBBA CONICET, Av. Patricias Argentinas 435, C1405BWE, Buenos Aires, Argentina

[3]Department of Cell Biology, University of Virginia, Charlottesville, Virginia 22903, USA

[4]Institute of Biological Chemistry, Academia Sinica, Taipei, 11529, Taiwan

[5]Glycosylation et différenciation cellulaire, EA 7500, Laboratoire PEIRENE, Université de Limoges, 123 avenue Albert Thomas, 87060 Limoges CEDEX, France

[‡]Corresponding author : Anne Harduin-Lepers, Unité de Glycobiologie Structurale et Fonctionnelle, UMR CNRS 8576, Université de Lille, Faculté des sciences et Technologies, 59655 Villeneuve d'Ascq, France. Phone: +33 320 33 62 46; Fax: +33 320 43 65 55; E-mail: anne.harduin-lepers@univ-lille.fr

orcid.org/0000-0002-1233-3799

Supplemental figures legends

Supplemental Figure S1 : Molecular Phylogenetic analysis by Maximum Likelihood (ML) method.

Supplemental FigureS2 : Molecular Phylogenetic analysis by Minimum Evolution (ME).

Supplemental Figure S1 : Molecular Phylogenetic analysis by Maximum Likelihood (ML) method.

The evolutionary history of vertebrate oligo- and mono-α2,8-sialyltransferases was inferred using the Maximum Likelihood method based on the JTT matrix-based model [1]. The tree with the highest log likelihood (-26151.14) is shown. Initial tree(s) for the heuristic search were obtained automatically by applying Neighbor-Join and BioNJ algorithms to a matrix of pairwise distances estimated using a JTT model, and then selecting the topology with superior log likelihood value. A discrete Gamma distribution was used to model evolutionary rate differences among sites (5 categories (+*G*, parameter = 1.3753)). The rate variation model allowed for some sites to be evolutionarily invariable ([+*I*], 4.68% sites). The tree is drawn to scale, with branch lengths measured in the number of substitutions per site. The analysis involved 147 amino acid sialyltransferase sequences. All positions with less than 95% site coverage were eliminated. That is, fewer than 5% alignment gaps, missing data, and ambiguous bases were allowed at any position. There were a total of 283 positions in the final dataset. Evolutionary analyses were conducted in MEGA7 [2].

Supplemental Figure S2 : Molecular Phylogenetic analysis by Minimum Evolution (ME).

The evolutionary history of vertebrate oligo- and mono-α2,8-sialyltransferases was inferred using the Minimum Evolution method and the optimal tree with the sum of branch length = 21.18281333 is shown. The tree is drawn to scale, with branch lengths in the same units as those of the evolutionary distances used to infer the phylogenetic tree. The evolutionary distances were computed using the JTT matrix-based method and are in the units of the number of amino acid substitutions per site. The rate variation among sites was modeled with a gamma distribution (shape parameter = 1.5). The ME tree was searched using the Close-Neighbor-Interchange (CNI) algorithm at a search level of 1. The Neighbor-joining algorithm [1] was used to generate the initial tree. The analysis involved 147 amino acid of vertebrate oligo- and mono-α2,8-sialyltransferases sequences, all positions with less than 95% site coverage were eliminated and there were a total of 283 positions in the final dataset. Evolutionary analyses were conducted in MEGA7.0 [2].

Additional references for supplemental figure legends :

1. Saitou N, Nei M: **The neighbor-joining method: a new method for reconstructing phylogenetic trees**. *Mol Biol Evol* 1987, **4**(4):406-425.
2. Kumar S, Stecher G, Tamura K: **MEGA7: Molecular Evolutionary Genetics Analysis Version 7.0 for Bigger Datasets**. *Mol Biol Evol* 2016, **33**(7):1870-1874.

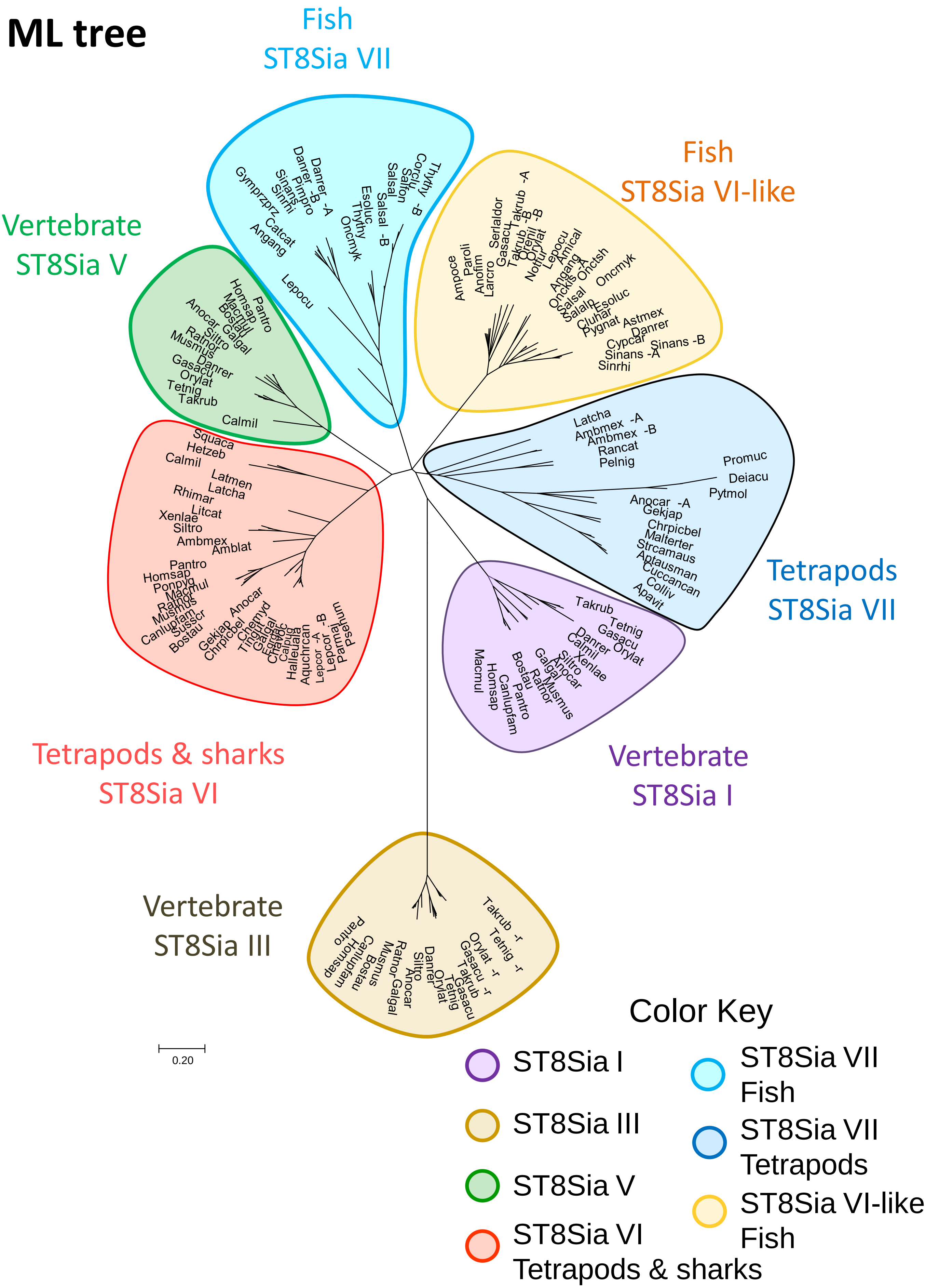
ML tree
Fish
ST8Sia VII
Fish
ST8Sia VI-like
Vertebrate
ST8Sia V
Tetrapods
ST8Sia VII
Tetrapods & sharks
ST8Sia VI
Vertebrate
ST8Sia I
Vertebrate
ST8Sia III
0.20
Color Key
ST8Sia I
ST8Sia III
ST8Sia V
ST8Sia VI
Tetrapods & sharks
ST8Sia VII
Fish
ST8Sia VII
Tetrapods
ST8Sia VI-like
Fish

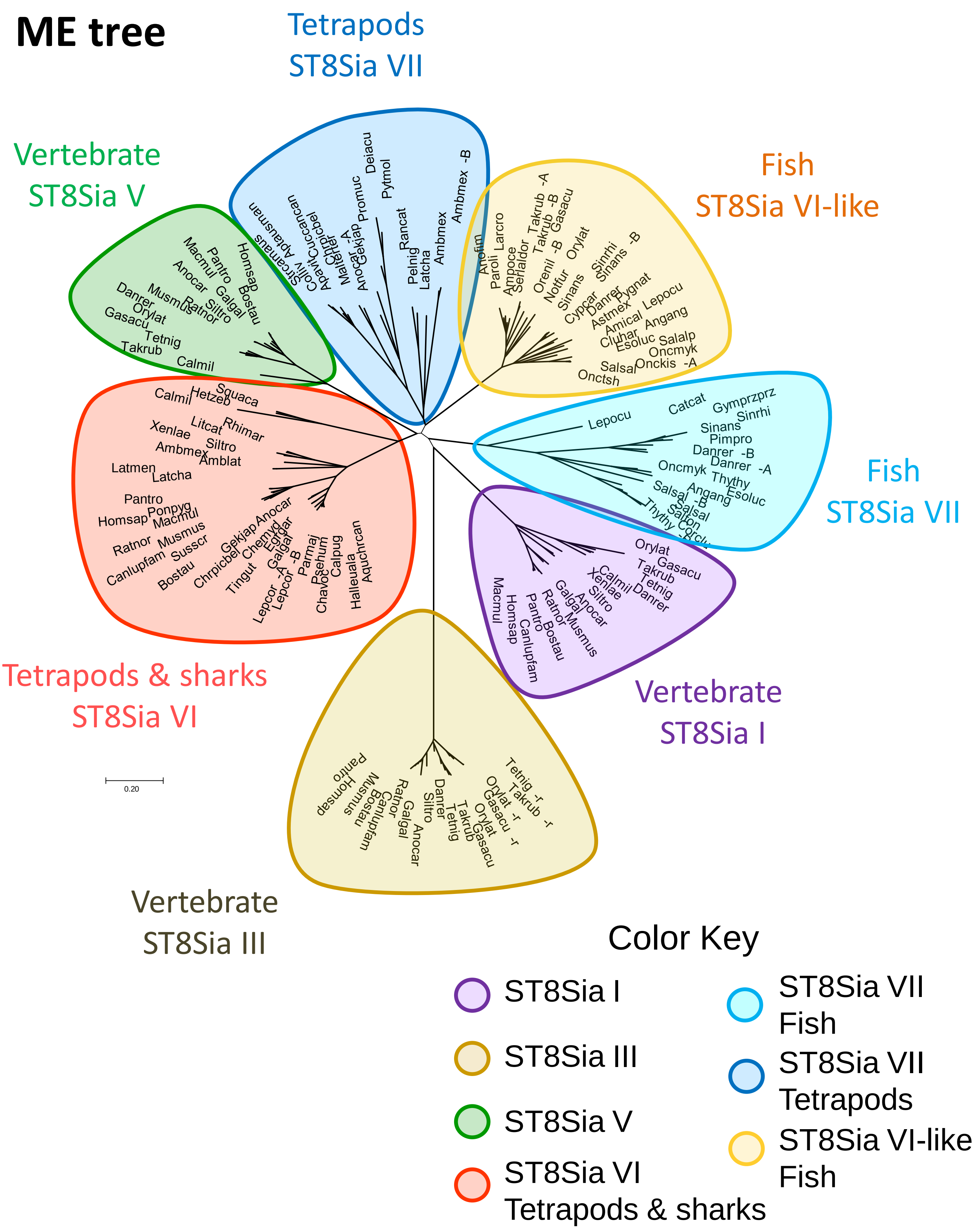

ME tree
Tetrapods
ST8Sia VII
Vertebrate
ST8Sia V
Fish
ST8Sia VI-like
Fish
ST8Sia VII
Tetrapods & sharks
ST8Sia VI
Vertebrate
ST8Sia I
Vertebrate
ST8Sia III
0.20
Color Key
ST8Sia I
ST8Sia III
ST8Sia V
ST8Sia VI
Tetrapods & sharks
ST8Sia VII
Fish
ST8Sia VII
Tetrapods
ST8Sia VI-like
Fish

Novel zebrafish mono-alpha2,8-sialyltransferase (ST8Sia VIII): an evolutionary perspective of alpha2,8-sialylation.

Lan Yi Chang[1], Roxana Elin Teppa[2], Maxence Noel[1], Pierre-André Gilormini[1], Mathieu Decloquement[1], Christine Thisse[2], Bernard Thisse[2], Anne Marie Mir[1], Virginie Cogez[1], Philippe Delannoy[1], Kay Hooi Khoo[4], Daniel Petit[5], Yann Guérardel[1], Anne Harduin-Lepers[1‡]

[1]Université de Lille, CNRS, UMR 8576 - UGSF - Unité de Glycobiologie Structurale et Fonctionnelle, F-59000 Lille, France

[2]Bioinformatics Unit, Fundación Instituto Leloir-IIBBA CONICET, Av. Patricias Argentinas 435, C1405BWE, Buenos Aires, Argentina

[3]Department of Cell Biology, University of Virginia, Charlottesville, Virginia 22903, USA

[4]Institute of Biological Chemistry, Academia Sinica, Taipei, 11529, Taiwan

[5]Glycosylation et différenciation cellulaire, EA 7500, Laboratoire PEIRENE, Université de Limoges, 123 avenue Albert Thomas, 87060 Limoges CEDEX, France

[‡]Corresponding author : Anne Harduin-Lepers, Unité de Glycobiologie Structurale et Fonctionnelle, UMR CNRS 8576, Université de Lille, Faculté des sciences et Technologies, 59655 Villeneuve d'Ascq, France. Phone: +33 320 33 62 46; Fax: +33 320 43 65 55; E-mail: anne.harduin-lepers@univ-lille.fr

orcid.org/0000-0002-1233-3799

List of supplementary data

Supplementary data1 : Full length sequences of 147 vertebrate mono- and oligo-α2,8-sialyltransferases.

Supplementary data2: Multiple sequence alignments of 147 vertebrate mono- and oligo-α2,8-sialyltransferases.

1 – Supplementary data1: Full length sequences of 147 vertebrate mono- and oligo-α2,8-sialyltransferases. Protein sequence of 147 selected vertebrate ST8Sia sequences: 129 mono-α2,8-sialyltransferases *i.e.* 17 ST8Sia I (green), 15 ST8Sia V (blue), 63 ST8Sia VI (black and teleost fish sequences in red) and 34 ST8Sia VII (violet) and 18 oligo-α2,8-sialyltransferases ST8Sia III (brown), which are used as an outgroup. Corresponding abbreviated names are as follows: Homsap: Homo sapiens; Pantro : Pan troglodytes; Macmul: Macaca mulatta; Musmus: Mus musculus; Ratnor: Rattus norvegicus; Bostau: Bos Taurus; Canlupfam: Canis lupus familiaris; Galgal: Gallus gallus; Siltro: Silurana tropicalis; Anocar: Anolis carolinensis; Susscr ; Sus scrofa ; Ponpyg: Pongo pygmaeus; Latcha: Latimeria chalumnae; Chemyd: Chelonia mydas; Chrpicbel: Chrysemys picta bellii; Aquchrcan: Aquila chrysaetos Canadensis; Egrgar: Egretta garzetta; Psehum: Pseudopodoces humilis; Lepcor: Lepidothrix coronata;Halleuala: Haliaeetus leucocephalus alascanus; Chavoc: Charadrius vociferous; Calpug: Calidris pugnax; Tingut : Tinamus guttatus ; Parmaj : Parus major ; Gekjap : Gekko japonicus ; Xenlae : Xenopus laevis ; Calmil : Callorhinchus milii ; Amblat : Ambystoma laterale ; Litcat: Rana catesbeiana; Hetzeb: Heterodontus zebra; Squaca: Squalus acanthias; Ambmex: Ambystoma mexicanum; Rhimar: Rhinella marina; Latmen: Latimeria menadoensis; Colliv: Columba livia; Deiacu: Deinagkistrodon acutus; Pytmol: Python molurus; Strcamaus: Struthio camelus australis; Apavit: Apaloderma vittatum; Promuc : Protobothrops mucrosquamatus ; Rancat : Rana catesbeiana ; Malterter : Malaclemys terrapin terrapin ; Gymprzprz : Gymnocypris przewalskii przewalskii ; Catcat : Catla catla ; Pimpro : Pimephales promelas ; Salfon : Salvelinus fontinalis ; Corclu : Coregonus clupeaformis ; Thythy : Thymallus thymallus ; Pelnig : Pelophylax nigromaculatus ; Aptausman : Apteryx australis mantelli ; Lepocu : Lepisosteus oculatus ; Anofim : Anoplopoma fimbria ; Amical : Amia calva ; Sinans : Sinocyclocheilus anshuiensis ; Sinrhi : Sinocyclocheilus rhinocerous ; Cypcar : Cyprinus carpio ; Pygnat: Pygocentrus nattereri; Onckis: Oncorhynchus kisutch; Esoluc: Esox Lucius; Salsal: Salmo salar; Astmex: Astyanax mexicanus; Salalp: Salvelinus alpinus; Cluhar : Clupea harengus ; Notfur : Nothobranchius furzeri ; Serlaldor ; Seriola lalandi dorsalis ; Larcro : Larimichthys crocea ; Paroli : Paralichthys olivaceus ; Ampoce : Amphiprion ocellaris ; Gasacu : Gasterosteus aculeatus ; Tetnig : Tetraodon nigroviridis ; Takrub: Takifugu rubripes; Gasacu: Gasterosteus aculeatus; Danrer: Danio rerio; Oncmyk: Oncorhynchus mykiss; Orylat: Oryzias latipes; Angang: Anguilla Anguilla; Orenil: Oreochromis niloticus; Onctsh: Oncorhynchus tshawytscha;

```
>Homsap_GT29_ST8Sia6

MRPGGALLALLASLLLLLLLRLLWCPADAPGRARILVEESREATHGTPAALRTLRSPATAVP
RATNSTYLNEKSLQLTEKCKNLQYGIESFSNKTKRYSENDYLQIITDIQSCPWKRQAEEYAN
FRAKLASCCDAVQNFVVSQNNTPVGTNMSYEVESKKEIPIKKNIFHMFPVSQPFVDYPYNQC
AVVGNGGILNKSLCGTEIDKSDFVFRCNLPPTTGDVSKDVGSKTNLVTINPSIITLKYGNLK
EKKALFLEDIATYGDAFFLLPAFSFRANTGTSFKVYYTLEESKARQKVLFFHPKYLKDLALF
WRTKGVTAYRLSTGLMITSVAVELCKNVKLYGFWPFSKTVEDIPVSHHYYDNKLPKHGFHQM
PKEYSQILQLHMKGILKLQFSKCEVA
```

>Pantro_GT29_ST8Sia6

MRPGGALLALLASLLLLLLLRLLWCPADAPGRARILVEESREATHGTPAALRTLRSPATAVP
RATNSTYLNEKSLHLTEKCKNLQYGIESFSNKTKGYSENDYLQIITDIQSCPWKRQAEEYAN
FRAKLASCCDAVQNFVVSQNNTPVGTNMSYEVESKKEIPIKKNIFHMFPVSQPFVDYPYNQC
AVVGNGGILNKSLCGTEIDKSDFVFRCNLPPTTGDVSKDVGSKTNLVTINPSIITLKYGNLK
EKKALFLEDIATYGEAFFLLPAFSFRANTGTSFKVYYTLEESKARQKVLFFHPKYLKDLALF
WRTKGVTAYRLSTGLMITSVAVELCKNVKLYGFWPFSKTVEDIPVSHHYYDNKLPKHGFHQM
PKEYSQILQLHMKGILKLQFSKCEVA

>Macmul_GT29_ST8Sia6

MRPGGALLALLASLLLLLLLRLLWCPADAPAHTRILVEESKEATHGTPAALRTLRSPATAVP
RATNSTYLNEKSLQLTEKCKNLQYGIESFSNNTKGYSENDYLQIITDIQSCPWKRQAEEYAN
FRAKLASCCDAVQNFVVSQNNTPVGTNMSYEVESKKKIPIKKNIFHMFPVSQPFVEYPYNQC
AVVGNGGILNKSLCGAEIDKSDFVFRCNLPPTTGDVSKDVGSKTNLVTINPSIITLKYGNLK
EKRALFLEDIATYGDAFFLLPAFSFRANTGTSFKVYYTLEESKARQKVLFFHPKYLKHLALF
WRTKGVTAYRLSTGLMITSVAVELCENVKLYGFWPFSKTVEDIPVSHHYYDNKLPKRGFHQM
PKEYSQILQLHMKGILKLQFSKCEVA

>Musmus_GT29_ST8Sia6

MRSGGTLFALIGSLMLLLLLRMLWCPADAPARSRLLMEGSREDTSGTSAALKTLWSPTTPVP
RTRNSTYLDEKTTQITEKCKDLQYSLNSLSNKTRRYSEDDYLQTITNIQRCPWNRQAEEYDN
FRAKLASCCDAIQDFVVSQNNTPVGTNMSYEVESKKHIPIRENIFHMFPVSQPFVDYPYNQC
AVVGNGGILNKSLCGAEIDKSDFVFRCNLPPITGSASKDVGSKTNLVTVNPSIITLKYQNLK
EKKAQFLEDISTYGDAFLLLPAFSYRANTGISFKVYQTLKESKMRQKVLFFHPRYLRHLALF
WRTKGVTAYRLSTGLMIASVAVELCENVKLYGFWPFSKTIEDTPLSHHYYDNMLPKHGFHQM
PKEYSQMLQLHMRGILKLQFSKCETA

>Ratnor_GT29_ST8Sia6

MRPGGTLFALVGSLMLLLLLRLFWCPADAPARSRMLGEESREETGGTSAALKTLWSPTTPVP
RTRNSTYLDEKTPETAEKCKGLKYSLDSLANKTRRYSEDDYLQIVTNIQRCPWKRQAEEYEN
FRAKLASCCDAIQDFVVSQNNTPVGSNMTYEVESKKQIPIRENIFHMFPVSQPFVDYPYNQC
AVVGNGGILNKSLCGAEIDKSDFVFRCNLPPITGSASQDVGSKTNLVTVNPSIITLKYKNLK
EKKARFLEDISAYGDAFLLLPAFSYRVNTGISFKVYQTLKESKVRQKVLFFHPRYLRHLALF
WRTKGVTAYRLSTGLMIASIAVELCENVTLYGFWPFSKTVEEIPLSHHYYDNKLPKHGFHQM
PKEYSQMLQLHMKGILKLQFSKCEAA

>Bostau_GT29_ST8Sia6

MRPGGALFALLASLLLLLLLRLLWCWTDAPARSRFLVEESREATHSSPAAPRKLRSSATLPP
RTGNSTYLNEKSLHLMERCQNLQAGLETLSNRTKGYSEDEYLQIITNIQSCPWKRQVEEYEN
FRAKLASCCDAVQNFIVSQNNTPIGTNMTYEVESKSKIQIKENIFDMLPVVQPFVGYSFNQC
AVVGNGGILNQSLCGAEIDKADFVFRCNLPPTTGNVTNDVGTKTNLVTLNPSIIKLRYGNLK
EKKAIFLEDVAAYGDAFVLLPAFSFRANTAASFKVYYTLKESKARQKVLFFHPKYLKNLALF

WRTKGVTEYRLSSGLMITSVAVELCENVKLYGFWPFSRTGENMPVSHHYYDNKLPKRGFHEM
PKEYSQILQLHVKGILKLQFSKCETA

>Canlupfam_GT29_ST8Sia6

MRPGGALLALLASLLLLLLLRLLCCSTDAPARSRLSAERSRDGPRGTPAAPRTLRSPATQLP
RPANSTYPNEKSLQLTEMCKSLRDGFQRLYSKAKNYLESDYLQIIRNIQNCPWKRQEEEYEN
FRAKLASCCDAAQNFIVSQNNTPAGTNMSYEVESKNEILIRENIFNMFPVSQPFVEYPYNQC
AVVGNGGILNKSLCGAEIDKSDFVFRCNLPPITGNISKDVGSKTNVVTVNPSIIRLKYGNLK
KKKEIFLEDIATYGDAFLLLPAFSFRANTIASFKVYSTLKESNARQKVIFFHPKYLRNLALF
WRTEGVTEFRLSSGLMITSVAVELCEHVKLYGFWPFSRTVKDTPVSHHYYDNNLPKRGFHEM
PKEYRQILQLHLKGILKLQFSKCEIA

>Galgal_GT29_ST8Sia6

MPAGQRPSGSGRRPVAALAGFDNFCYPVLLARGWEEEAEVATVTPKVVRALRSPLTSLPQTE
NRTTANKDGIYQFELDTKCKAIQDNILSSSFKKKRYPEDYYLHIVKKLQNCTWVRRPEESAK
FRSELASCCNAVHNFIASQNNTPLGSNMSYEVDSKKTILITEDIFRMLPVSSPLSVYPFKNC
AVVGNGGILKNSSCGAEIDRSDFVFRCNLPPTMGSISKDVGNKTKLVTVNPSIIAQKYNKLN
EKKTEFVENVAVYGDAFLLLPAFSFRSNTATSFKVYHTLQEFKATQRAIFFHPTYLKNLAQF
WRTKGVKAYRLSSGFMITSAAVELCENVKLYGFWPFSKSTEKMPISHHYYDNQLPKPGFHAM
PKEYNQILQLHGKGIKKLQFGKCESD

>Siltro_GT29_ST8Sia6

MRGHNMKKTLLVLLGTSLLLFVLTVWLSIGESAHYRPGTVTSTFGLVKPEVNFIVTSQSNYT
TSPVNNSYCRDVQDRLLTVSLKKRYSEDYYIQTVNDLQNCTWHKRPEERSKFRLDLSSCCNG
VKNFIVSQNNTSVGINITYEVESKKRILITEEIYRMLPKSQPFDGTPFKQCAVVGNGGILAN
SGCGVEIDQSDFVFRCNLPPTSGNVSVDVGNKTNLVTVNPSIISQKYRKLNKVKNVFLKYVS
NYGNSLLLLPAFSYSSNTAISFEVHRVLEKNQAKQKAVFFHPNYLKNLAKFWKGKGVRAYRL
STGLMITSAAMELCEEVILYGFWPFSKDLEGKPISHHYYDNMLPKPGFHAMPKEFYQVLQLH
HKGVLRLQIGKCEKR

>Takrub_GT29_ST8Sia6-A

MRGKFLKTRFCLGIVIVCLMGLLSILSWYLSDNKQESHSSWKTGSPPAVGRTVLRLQEITDK
VNRLYAQTLEKKEDKSKILRSELSSKCQGFEKAIITQANTVVGSKIVYDGERKSLVQVTPEM
FNTFPKEHPFPNKTWETCAVVGNGGILSDSGCGKMIDSAQFVIRCNLPPLNNGYQDHVGVKT
DLVTANPSILVEKYGALMGRRRPFIESLRSYGNSLLLLPTFSYRSNTPVSLRAFYSIEDFGS
PTRAISFNPQYLQKLDVFWRSKGLRAVRLSSGLMVASLALELCSNVHLYGFWPFSNHPNGLR
TLKNHYYDDIPPKKKFHAMPVEFELLLKLHTEGVLRLHLDDCVPGEVNLMGSTGGDGRP

>Takrub_GT29_ST8Sia6-B

MRGQFLKNLFSLGIVVVCLGGLLTTLSWYLSDNNKNHTAPVRQEVPQQADDLSSGCREIIDK
VNRLYAQTWEKKEDKYKKFRSELSSKCQGFEKAIITQANTVVGSKIVYDGERKRSVKVSTEM
FNTFPKEHPFPNKTWETCAVVGNGGILSDSGCGKMIDSAQFVIRCNLPPLNNGYQDHVGVKT
DLVTANPSILFEKYGALMGRRRPFIESLRSYGNSLLLIPAFSYGNNTPVSLRAFYSIEDFGS

PTRTIFFNPQYLQKLAVFWRSKGLRAVRLSSGLMVASLALELCSNVHLFGFWPFSNHPHGLR
TLKNHYYDDIQTKKKFHAMPVEFELLLQLHTEGVLRLHLDDCVPGEVNLMGSTRGDGPPL

>Gasacu_GT29_ST8Sia6

MRGRRLKPLFSLGIALFCLGILLTALVWYNGNSNVETRQLSLEKKRAPPPSELCKGCRQFID
KVRERYNKTWIKQVDDYLEFRSQLSRKCHGFDSAFITQYNTPVGAKIVYDGEKTRTLQVTPE
IFSTFPKEHPFSNKIWGTCSVVGNGGILSNSSCGKMIDSAEFVMRCNLPPLDNGYENDVGIK
TDLVTANPSILIKKYGSLQQRRRPFVESLRSYGNSLLLLPAFSYGFNTPLSLRAVYSIEDFK
SPTRPVFFNPEYLESLGLFWRSRGLKAVRASTGLMMASLALEHCTDVHLYGFWPFGNHPQGL
HALTNHYYDDVQPKNTVHAMPVEFEFLLQLHSQGVLRLHLEDCQPGEK

>Danrer_GT29_ST8Sia6

MRVMRTLMRWLLPVILLCSFCSVAFWIFISNNNVIPHPASRIPQKASNTQSCKACKDSVIIG
KALGNYSNSWKKHEANYKRFRLLLNEKCHAVSKAVVTQNNTPLGSNVVYDGERRKPLQVTQA
LYNILAKEQPFGNATWESCAVVGNGGVLANSSCGEEINSAQFVIRCNLPPLDDRYEKDVGNK
TNLVTANPSILHEKYSGLMERRRPFVESLHSYGQALLLLPAFSYGHNTPVSLRAFYTLEDFG
REGPLPIFLNPEYLRKLTKFWREQGLNSVRPSTGLIMASLALEICTNVHLYGFWPFGKHPND
SRPITNHYYDNRESKKNVHSMPSEFEQLLKLHKQGVVHIHLGECQPAHR

>Oncmyk_GT29_ST8Sia6

MRGLFSFMLTLSILGTVLTALVWYFFSKHNVQPGRPPHKSPIMNSGPFPSRTCKGCRDTVTD
KVLELYSHSWRKQEDKFRKFRSLLSNRCHGLTKAMVTQANTPLGSKVVYDGEKRKPLQVTPE
LFSTFAKENPFVNTTWDTCSVVGNGGILANSSCGEKIDSAQFVIRCNLPPLENGYERDVGNK
TDLVTANPSILMEKYGGLQERRRPFVESLRSYGDSLMLLPAFSYGHNTPVSLRALYTIQDFD
SPSRPVFLNPEYLQSLARFWQGQGLRTVRLSTGLIVASLALELCANVHLYGFWPFNEHPHRH
QPLTNHYYDNRQSKKTVHAMPAEFDHLLRLHTQGVLRIHLGECAPTASPGCSSKMAWTPASV
SGLSLPVGSLGSAMPTLVQRNTTSQSRKPWAGPDTNVSGPSGL

>Anocar_GT29_ST8Sia6

MHRAVLSLAALLGSLLLCLCLAWLSADGSSGARGWEKELRATTAESSPRVMMMMMRSLRSPT
APLPRPPTGSLAPNRTPSNQTGTQKSEQVSKCKETWNNNWSSSIKKKRYSEDYYLQIVGKLQ
NCTWKKRPQEYAKFRSELTSCCDAIHNFISSQNNTPLGSNMSYEVDNKKTIHITEEIFKMLP
ESQPLDYPFKQCAVVGNGGILKNSNCGAEIDKSDFVFRCNLPPTEGSVSQDVGNKTNLVTVN
PSIIAQKYNKLNEKKATFLENIASYGDAFLLLPAFSFRSNTATSFKVHHTLREFSAKQKAIF
FYPRYLKNLAQFWRTKGVKAYRLSSGFMITSAAVELCENVKLYGFWPFSKSIAGNPISHHYY
DNQLPKPGFHAMPKEYNQILQLHGRGILKLQLGKCHTE

>Susscr_GT29_ST8Sia6

MRPGGALLALLASLLLLLLLRLLWCSTDAPASSRFLVEESREATHSTPAALRTLRSRATQPP
HTTSSTYLNAKSLQPTERCQNLQDDLKALSNKTRKYSEDDYLQIITNIQSCPWKRQAEAYEN
FRAKLASCCDAVQNFIVSQNNTPVGTNMSYEVESKSEIQIRESIFDMLPVFQPFIGYPYNQC
AVVGNGGILNQSLCGAEIDKSDFVFRCNLPPTTGNISNDVGSKTNLVTLNPSIIRLRYGNLK
EKKAVFLEDIATYGDAFVLLPAFSFRANTAASFKVYYALKESNSRQKVLFFHPKYLKHLAVF

WRTKGVTEYRLSSGLMIASVAVELCENVKLYGFWPFSKTGENTPVSHHYYDNKLPKRGFHEM
PKEYRQILQLHVKGILQLQFSKCDTS

>Orylat_GT29_ST8Sia6

MKRHPLNELFSMMITFLFVGSLLTSLGWYMVDSNNVHPPLPPQKKSPPKLPDPCKNCDENIM
KALELYSEPWKKQEDNYHQFRSQLNSKCHGLEKAIITQANTPQGTKLVYDAERKRTLVVNAE
VFNTFIKENPFPNKTWDTCAVVGNGGILANSSCGKTIDSAQFVIRCNLPPLSNGFEKDVGIK
SDIVTANPSIITEKYFSLMRHRRPFAEAMRIYGNSMVLLPAFSFGHNTALSMRAFYTLEDFE
SSARAVYFNPEYLKNLANFWRSEGLKSPRLSTGIMMASIALEVCSEVHLYGFWPFDVHPYSH
QGLTNHYYDDRKAKNKFHAMPTEFNLLLQLHRKGVLRLHLGDCTPDEK

>Ponpyg_GT29_ST8Sia6

MRPGGALLALLASLLLLLLLLRLLWCPADAPARARIPVEESREATHGTPAALRTLRSPATAVP
RATNSTYLNEKSLQLTEKCKNLQYGIESFSNKTKGYSENDYLQIITDIQSCPWKRQAEEYAN
FRAKLASCCDAVQNFVVSQNNTPVGTNMSYEVESKKEIPIKKNIFHMFPVSQPFVEYPYNQC
AVVGNGGILNKSLCGTEIDKSDFVFRCNLPPTTGDVSKDVGSKTNLVTINPSIITLKYGNLK
EKKALFLEDIATYGDAFFLLPAFSFRANTGTSFKVYYTLEESKARQKVLFFHPKYLKDLALF
WRTKGVTAYRLSTGLMITSVAVELCKNVKLYGFWPFSKTVEDTPVSHHYYDNKLPKRGFHQM
PKEYSQILQLHMKGILKLQFSKCEVA

>Latcha_GT29_ST8Sia6

MWMCRMRKCILLWILLASGVFFLLTFWIKYDAALHYREIANKDWRDCLATACGGRRGMRELE
SAVSPHRPLPTSNSSNTNQNGSTFNKLNLLKADSSSCKELHNRMSATTPRKRYSDDYYIQTV
NELQKCSWVIRPEEHEKFRSELSVCCNAVRNFIVSQNNTPLGTNMSYEVENGRTFLITQRIF
KMFPQSQPFAGYPYNQCAVVGNGGILKNSACGTEIDQADFVFRCNLPPTLGNISIDVGSKTS
LVTLNPSIITHRFGKLNEKRKPFVETVSHYGEAFLLLPAFSFRSNTALSFKVYHTLEAFRGK
QKTIFFHPKYLKSLALFWRSKGIKVYRLSSGFMIANAAIELCKEVRLYGFWPFSKNNEGKNI
SHHYYDNQLPKPGIHSMPKEFYHFLKLHNKGIIKLQFGRCDIT

>Chemyd_GT29_ST8Sia6

MRAVIALIAMLSSLLLFLCLLWLTADGSLSTRSWEKETKSATDITPKGLRKLRSPLTSVQLA
TQTENRTTHKNAIYQLQLASKCKEMQDYIWSSSIKKKRYSEDYYLQIVTKIQNCTWKRRPEE
YTKFRLELASCCDAVHNFIASQNNTALGSNMSYEVDNKKTLLITEDIFRMLPESPPLLDYPF
KHCAVVGNGGILKNSSCGAEIDKSDFVFRCNLPPTTGSVSKDVGNKTNLVTVNPSIIAQKYN
KLNEKKVTFLENIASYGNAFLLLPAFSFKSNTAASFKVYHTLQEFRAKQRAIFFHPRYLKSL
AQFWRTKGVKAYRLSSGFMIASAAIELCENVKLYGFWPFSKTTEEMPISHHYYDNQLPKPGF
HAMPKEYNQILQLHGKGIVKLQFGKCDSDXKGTSDSSKIKHFRLSFRAKLPFLQPGAVPQIC
TLFPKRL

>Chrpicbel_GT29_ST8Sia6

MRAVIALIAMLSSLLLFVCLLWLTADGSLSTRSWEKETKSATDVTPKGLRKLRSPLTSVQLA
TQTENRTILKNAIYQLQQASKCKEMQDNIWSSSIKKKRYSEDYYLQIVTKIQNCTWKRRPEE
YTKFRLELASCCDAVHNFIASQNNTLLGSNMSYEVDNKKTILITEDIFRMLPESPPFLGYPF

KHCAVVGNGGVLKNSSCGAEIDNSDFVFRCNLPPTTGSVSKDVGNKTSLVTVNPSIIAQKYN
KLNEKKVTFLENIASYGNAFLLLPAFSFKSNTAASFKVYHTLQEFRAKQRAIFFHPRYLKNL
AQFWRTKGVKAYRLSSGFMIASAAIELCENVKLYGFWPFSKTTEEMPISHHYYDNQLPKPGF
HAMPKEYNQILQLHGKGILKLQFGKCDSD

>Aquchrcan_GT29_ST8Sia6

MRAVVALAAALGSLLLGGCLLRLGGQQPLRARGWEEDAGVATVTPKVVRALRSPLTSLPQTE
NRTTVNKNGIYQFEQASKCKAIQDNILSSSIKKKRYSEDYYLHIVTKLQNCTWIRRPEESTK
FRSELASCCDAVHNFIASQNNSPLGSNMSYEVDSKKTILITEDIFKMLPVSSPLSVYPFKTC
AVVGNGGILKNSSCGAEIDHSDFVFRCNLPPTTGSISKDVGNKTNLVTVNPSIIAQKYNKLN
EKKTEFLENIAVYGDAFLLLPAFSFRSNTATSFKVYHTLQEFKATQRAIFFHPTYLKSLAQF
WRTKGVKAYRLSSGFMITSAALELCENVKLYGFWPFSKSTEKMPISHHYYDNQLPKPGFHAM
PKEYNQILQLHGKGILKLQFGKCESD

>Egrgar_GT29_ST8Sia6

MPRQTATEGSVRGLGWVSCSHSTGASPHPSEQQRANSSVLWPTSAPLGTEEIQGVESQKLDL
LWRITVPYKLARGWEEDAAVAAVTPKVVRALRSPLPSLPQTENRTNVNKKGIYRFEQASKCK
AIQDNILSSSIKKKRYSEDYYLHIVTKLQNCTWIRRPEESMKFRSELASCCDAVHNFIASQN
NTPLGSNMSYEVDSKKTILITEDIFKMLPVSSPLSVYPFKTCAVVGNGGILKNSSCGAEIDR
SDFVFRCNLPPTTGSISKDVGNKTNLVTVNPSIIAQKYNKLNEKKTEFLEDVAVYGDAFLLL
PAFSFRSNTATSFKVYHTLKEFRASQRAIFFHPTYLKSLAQFWRTKGVKAYRLSSGFMITSA
ALELCENVKLYGFWPFSKSTEKMPISHHYYDNQLPKPGFHAMPKEYNQILQLHGKGILKLQF
GKCESD

>Psehum_GT29_ST8Sia6

MRAVIALAAALGSLLLGGCLLRLGGQQPLRARGWEEEAGVAAVTPKVVRALRSPLTPLPHTE
NRYGLAARRTTLNKNGIYQFEQASKCKAIQDNILSSSIKKKRYSEDYYLHIVTKLQNCTWIR
KPEESTKFRSELASCCDAVHNFIASQNNSPLGSNMSYEVDSKKTILITEDIFKMLPVSSPLS
VYPFKNCAVVGNGGILKNSSCGAEIDSSDFVFRCNLPPTTGNISKDVGNKTNLVTVNPSIIA
QKYNKLNEKKTEFLENIAAYGDAFLLLPAFSFRSNTATSFKVYHTLKEFKATQRAIFFHPSY
LKSLAQFWRTKGVKAYRLSSGFMITSAALELCENVKLYGFWPFSKSIEKMPISHHYYDNQLP
KPGFHAMPKEYNQILQLHGKGVLKLQFGKCESD

>Lepcor_GT29_ST8Sia6-B

MRAVIALAAALGSLLLGGCLLRLGGQQPLRARGWQEEEAGVAAVTPKVVRALRSPLTPLPQT
ENRTTANKNGIYQFEQASKCKAIQDNILSSSIKKKRYSEDYYLHIVTKLQNCTWIRRPEEST
KFRSELASCCDAVHNFIASQNNSPLGSNMSYEVDSKKTILITEDIFKMLPVSSPLSVYPFKT
CAVVGNGGILKNSSCGAEIDSSDFVFRCNLPPTTGSISKDVGNKTNLVTVNPSIIAQKYNKL
NEKKTEFLENIAVYGDAFLLLPAFSFRSNTATSFKVYHTLKEFKATQRAIFFHPAYLKSLAQ
FWRTKGVKAYRLSSGFMITSAALELCENVKLYGFWPFSKSTEKMPISHHYYDNQLPKPGFHA
MPKEYNQILQLHGKGILKLQFGKCESD

>Halleuala_GT29_ST8Sia6

MRAVVALAAALGSLLLGGCLLRLGGQQPLRASPVLLARGWEEDAGVATVTPKVVRALRSPLT
SLPQTENRKPVNKNGIYQFEQASKCKAIQDNILSSSIKKKRYSEDYYLHIVTKLQNCTWIRR
PEESTKFRSELASCCDAVHNFIASQNNSPLGSNMSYEVDSKKTILITEDIFKMLPVSSPLSV
YPFKTCAVVGNGGILKNSSCGAEIDHSDFVFRCNLPPTTGSISKDVGNKTNLVTVNPSIIAQ
KYNKLNEKKTEFLENIAVYGDAFLLLPAFSFRSNTATSFKVYHTLQEFKATQRAIFFHPTYL
KSLAQFWRTKGVKAYRLSSGFMITSAALELCENVKLYGFWPFSKSTEKMPISHHYYDNQLPK
PGFHAMPKEYNQILQLHGKGILKLQFGKCESD

>Lepcor_GT29_ST8Sia6-A

MRAVIALAAALGSLLLGGCLLRLGGQQPLRARGWQEEEAGVAAVTPKVVRALRSPLTPLPQT
ENRYGLPARRTTANKNGIYQFEQASKCKAIQDNILSSSIKKKRYSEDYYLHIVTKLQNCTWI
RRPEESTKFRSELASCCDAVHNFIASQNNSPLGSNMSYEVDSKKTILITEDIFKMLPVSSPL
SVYPFKTCAVVGNGGILKNSSCGAEIDSSDFVFRCNLPPTTGSISKDVGNKTNLVTVNPSII
AQKYNKLNEKKTEFLENIAVYGDAFLLLPAFSFRSNTATSFKVYHTLKEFKATQRAIFFHPA
YLKSLAQFWRTKGVKAYRLSSGFMITSAALELCENVKLYGFWPFSKSTEKMPISHHYYDNQL
PKPGFHAMPKEYNQILQLHGKGILKLQFGKCESD

>Chavoc_GT29_ST8Sia6

MSKTGKLVTMDKERGIRGEESGAAHARWCLIPGFDNFVSPLSLGRGWEEDAGVAAVTPRVVR
ALRSPLPSLAQGGNRTTVNKNGIYQFEQASKCKAIQDNILSSSIKKKRYSEDYYLHIVTKLQ
NCTWTRRPEESTKFRSELASCCDAVHNFIASQNNTPLGSNMSYEVDSKKTILITEDIFKMLP
VSSPLSAYPFKTCAVVGNGGILKNSSCGAEVDRSDFVFRCNLPPTTGSISKDVGNKTNLVTV
NPSIIAQKYNKLNEKKTEFLENIAVYGDAFLLLPAFSFRSNTATSFKVYHTLQEFKATQRAI
FFHPTYLRSLAQFWRTKGVKAYRLSSGFMITSAALELCENVKLYGFWPFSKSTEKMPISHHY
YDNQLPKPGFHAMPKEYNQILQLHGKGILKLQFGKCESD

>Calpug_GT29_ST8Sia6

MRAVIALAAALGSLLLGGCLLRLGGQQPLRARGWEEEEEEEAGAAAVTPKVGRALRSPSAAL
PQAGNRTTVNKNGIYQFEQASKCKAIQDNILSSSIKKKRYSEDYYLHIVTKLQNCTWIRRPE
ESTKFRSELASCCDAVHNFIASQNNTPLGSNMSYEVDSKKTVLITEDIFKMLPVSSPLSVYP
FKTCAVVGNGGILKNSSCGAEIDRSDFVFRCNLPPTTGSISKDVGNKTNLVTVNPSIIAQKY
NKLNEKKTEFLDNIAVYGDAFLLLPAFSFRSNTATSFKVYHTLQEFKATQRAIFFHPTYLKS
LAQFWRTKGVKAYRLSSGFMITSAALELCENVKLYGFWPFSKSTEKMPISHHYYDNQLPKPG
FHAMPKEYNQILQLHSKGILKVQFGKCESD

>Tingut_GT29_ST8Sia6

MLMSRLEHCQDELIWKRPCKLLGEVPAAMTQAPLRAPSQGQGANVPQSMLPGTEEMQAAVWQ
KVMFIELSLVYSHFSWFKLQLQKKDHFQLTELKKQRGETTISDKNGIYQFEQASKCKEIQDN
ILSSLTKKKRYSEDDFLHLVTKLQNCTWVKRPEECTKFRSELASCCDAVHNFVASQNNTPLG
SNMSYEVESKKTILITENIFRMLPVSSPFSAYPFKNCAVVGNGGILKNSSCGAEIDRADFVF
RCNLPPTTGSMSKDVGNKTNLVTVNPSIIAQKYNKLNKKKTEFLQNIAVYGDAFLLLPAFSY
RSNTATSFKVYQTLKEFKAMQRAIFFHPTYLKSLAQFWRTKGVKAYRLSSGFMITSAAIELC

ENVKIYGFWPFSKSTEKMPISHHYYDNQLPKPGFHAMPKEYNQILQLHGKGILKLQFGKCES
E

>Parmaj_GT29_ST8Sia6

MRAVIALAAALGSLLLGGCLLRLGGQQPLRARGWEEEAGVAAVTPKVVRALRSPLTPFPHTE
NRYGLAARRTTLNKNGIYQLEQASKCKAIQDNILSSSIKKKRYSEDYYLHIVTKLQNCTWIR
KPEESTKFRSELASCCDAVHNFIASQNNSPLGSNMSYEVDSKKTILITEDIFKMLPVSSPLS
VYPFKNCAVVGNGGILKNSSCGAEIDSSDFVFRCNLPPTTGNISKDVGNKTNLVTVNPSIIA
QKYNKLNEKKTEFLENIAAYGDAFLLLPAFSFRSNTATSFKVYHTLKEFKATQRAIFFHPAY
LKSLAQFWRTKGVKAYRLSSGFMITSAALELCENVKLYGFWPFSKSIEKMPISHHYYDNQLP
KPGFHAMPKEYNQILQLHGKGILKLQFGKCESD

>Gekjap_GT29_ST8Sia6

MPGIELACQVDAPYLSHGPAPRWKLDLQDDGRLEQLTDMIKVTMEKPPLRWRSLTTWACLAC
LLARGWEKEVKAATDAAPRVMRSLRSPVASLQLTPHMATNRTTPNQSGIQKPETASKCKEMW
SNSWAASVKRKRYAEDYYLQVVGRLQNCTWNKRPQEYAKFKSELASCCDAAHNFITSQNNTP
LGSNMSYEVDNKKTIHITEEIFRMLPESQPLEQPFKNCAVVGNGGILKNSNCGAEIDQSDFV
FRCNLPPTMGSIRQDVGSKTNLVTINPSIISQKYNKLNEKKATFLENIASYGDTFLLLPAFS
FRSNTAASFKVHHTLKEFSAKQKAIFFYPRYLRNLAQFWRTKGVKAYRLSSGFMITSAALEL
CENVKLYGFWPFSKNVAGTPISHHYYDNQLPKPGFHAMPKEYNQILQLHGRGILKLQFGKCL
PD

>Xenlae_GT29_ST8Sia6

MRGRNMKKTLFLLLGTSLLLFILTVWLSIGESARYRSGTVTSRVGLVKPELSYTVPSQSNYT
TSPVISSFCRDVEDRLLTVSLKKRYSEDYYIQTVNELQNCTWQKRPQEYSKFRLYLSTCCNG
LKNFIVSQNNTSLGSNITYEVESKKKILIAEEIYRMFPKSQPFVGAPFKQCAVVGNGGILAN
SGCGAEIDQSDFVFRCNLPPTWGNISVDVGNKTNLVTVNPSIISRKYRKLNKVKNVFLKNVS
NYGYSFLLLPAFSYSSNTAISFEVHRILEKNQAKQKAIFFHPYYLKNLAQFWKGRGVRAYRL
STGLMITSAAIELCEEVKLYGFWPFSKNQEGKPISHHYYDNILPKPGFHAMPKEFYQVLQLH
HKGVLRLQIGKCEKR

>Calmil_GT29_ST8Sia6

MVHQCVVRTVLISCGVCIFLFGILVNFEEPLNYRSQANQNESLDVPTDCSSCESTSQRLKSL
FLLEDKRPACEKLYNRIASASNQWRSEEDVLRNIAKIHQCKWGKQKRAVENFRWELRKCCRT
LSGSFVTQRNTPVGTELFYEAEPKKKIKITPSIFAIFPKDSPFRGRSIQRCAVVGNAGILHN
SSCGAEIDQADFVFRCNLPPMGGNFTKDVGSKTHLVTANPKVIIERYAELHKRRKPFANTLV
IYNDALLLFPAFYHSKNTALSFRAHYTLQDFKSKQRVIFFNPIYLKHLAHFWLSKGMQVQNL
STGITVASMAMELCSEVWIYGFWPFGKNTEGELMSHHYFDSLLAEPDLHSTSNEFYQLLRMH
SKGIVKLQMGQCEAEETDEFTLKPG

>Amblat_GT29_ST8Sia6

MGARGMKMFTLLVFLSTCLLLLSFWITSGELPTSRDKQTKERNIFTNNSQPGHSQQLKRNGG
ATTSKPDHHTSGSNLRSANSSVAFRFLKEDKLYLIQTNAQSNSVAKVQLCKETQDRYLSATL

RKRYSEDFYIQSFRKVQNCTWTRRPEEYYKFRSKLASCCNAVNNFIVSQNNTSLGSNMSYEV
DNKKNIVISETIFKMLPQSQPFVERSYKHCAVVGNGGILQNSSCGAEIDESDFVFRCNLPPV
NGDFHKDVGNKTNVVTVNPSIIALRYGKLSQKKTVFLQNITKYGDAYFLLPAFSYRSNTAVC
FKVFNALKEAKANQKAIFFHPKYLKNLGQFWRANGVRAYRLSTGLMIASAAIELCDHVKLYG
FWPFSKNTEGNLISHHYYDNQLPKPGFHAMPKEFIQYLQLHNKGILKLQVGECENKTKI

>Lepocu_GT29_ST8Sia6

MRKALLMLLFSLTLCLVIVGIMVTSLWSTLSNRSEAARGALKLVAGKQAATQCKECREKANI
DKLAQVHSQTWRKQESRLQTFRAQLNMKCQGFSRAIITQANTLLGSKVTYDGERRKPVEVTP
KLYSTFPKEHPFGNVSFQSCAVVGNGGILANSSCGEEIDGAQFVIRCNLPPVDRKYQDDVGN
KTDLVTANPSILLERFEGLMELRRPFVESLGDYGQPMLALPAFSYGHNTPVSLRAVYTLQDF
NSPVRAVFLNPEYLQNLARFWKAHGLRTVRLSTGLIVASLALELCASVTLYGFWPFSLHPFS
KQHLTNHYYDDQQSKKSFHAMPAEFEQLLRLHSQGVIRVHLGSC

>Anofim_GT29_ST8Sia6

MRGHLLQSFFSLVITLFFLGSLMTTLIWYMVDTNNVKPQRPSPQKKSDPQPSELCKGCREVI
NKVIERYSKTWKKQEENYQKFRSQLSSKCHGFDKAIITQANTPVGAKLVYDGEKKRTLQVTP
EIFSTFAKEHPFQNKIWDTCAVVGNGGILTNSSCGKTIDSAQFVMRCNLPPLDKGYEKHVGI
KTDLVTANPSILLEKYGALMGRRRPFVESLRSYGNSLLLLPAFSFGFNTPVCLRAVYSIEDF
ESPTRPVFFNPEYLQKLALFLRSQGLRAPRPSTGIIMASLALEHCANVHLYGFWPFSSHPHG
LHALTNHYYDDRQTKKKFHSMPAEFDLLLQLHSQGVLRLHLGDCPPGER

>Amical_GT29_ST8Sia6

MRRFIVKLLFSLMLCLFILGTVLMSFWYTFSYSNVRVTHPAVKKTVSEKCQNCREQSVIDRL
LQTHIVDWEKQEHKFQSFRAQLNSRCDGLSKAIISQTNTPLGSKVVYDGEKRKPIQVTPKLF
STFPKEHPFGNRTFTSCAVVGNGGILMNSSCGGEIDEAQFVIRCNLPPVDHGYQRDVGNKTS
LVTANPSILLDKFNGLMELRRPFVDSLGSYGDPLLVLPAFSYSRNTPVSLRALYTLQDFDSP
VRPVFLNPEYLHNLARFWKAQGLQAIRLSTGLIMASLALELCANVQLYGFWPFSKDPHSKHP
LTNHYYDDQQAKKTIHAMSTEFSHLLMLHNQGIIRVQLGKCQAGTRRATPSLHPPVSPLRH

>Sinans_GT29_ST8Sia6

MRVMRTLMLWLFPVILLCSFCSVAFWIFLSNNNVIPHPSSRIPQKSSETKSCKGCSKDNVLI
AKALENYSHKWKKHEANFKRFRSLLSSKCHAVSKAVVTQNNTPLGSNVIYDGERRKPLQVTQ
ALFNILAKEQPFGNATWESCAVVGNGGILANSSCGEEINSAQFVIRCNLPPLDNRYEKDVGN
KTSLVTANPSILHEKYSGLMERRRPFVESLRPYGQALLLLPAFSYGHNTPVSLRAFYTLEDF
GSDSPLPVFLNPEYLRRLSKFWRERGLNSVRPSTGLIMASLALEICSNVHLYGFWPFNKHPN
DSRPITNHYYDDRESKKNVHSMPTEFEHLLKLHKQGVIRIHLGECQPT

>Sinrhi_GT29_ST8Sia6

MRVMRTLMLWLLPIILLCSFCSVAFWIFLSNNNVIPHPSSRIPQKSSETKSSKGCSKDNVLI
AKALENYSHKWKKHEANFKRFRSLLSSKCHAVSKAVVTQNNTPLGSNVIYDGERRKPLQVTQ
ALFNILAKEQPFGNATWESCAVVGNGGILANSSCGEEINSAQFVIRCNLPPLDNRYEKDVGN
KTSLVTANPSILHEKYSGLMERRRPFVESLRPYGQALLLLPAFSYGHSTPVSLRAFYTLEDF

GSDSPLPVFLNPEYLRRLLKFWRERGLNSVRPSTGLIMASLALEICSNVHLYGFWPFNKHPN
DSRPITNHYYDDRESKKNVHSMPTEFEHLLKLHKQGVIRIHLGECQPT

>Sinans_GT29_ST8Sia6-B

MHVMRTLMLWLLPVIFLCSFCSVAFWIFLSNNNVIPHPLSRIPQKRPETQSCKECKDSVLIA
KALENYSDKWKKHEANLKRFRSLMSSKCHAVSKAVVTQNNTPLGSNVIYDGERRKPLQVTQA
LFNILAKEQPFGNATWESCAVVGNGGILANSSCGEEINSAQFVIRCNLPPLDKGYEKDVGKK
TNLVTANPSILHEKYSGLMERRRPFVESLRPYGQALLLLPAFSYGHNTPVSLRAFYTLEDFG
SNSPLPVFLNPEYLRRLSKFWREQGLNSVRPSTGLIVASLALEICSNVHLYGFWPFSKHPYD
SQPITNHYYNDRESKKNVHSMPNEFAHLLKLHKQGVIRIHLGECQPTHS

>Cypcar_GT29_ST8Sia6

MRVMRTLMLWLLSIILLCFFCSVTFWIFLSNNNVNPRPSYKIPQKRQSCKECNDDVLIAKAL
ENYSDKWKKQETNFKKFRSLLSNKCHAVSKAVVTQNNTPLGSNVIYDGERRKPLHVTQALFN
ILAKEQPFGNATWESCAVVGNGGILANSSCGEEINSAQFVIRCNLPPLDKGYEKDVGNKTNL
VTANPSILHEKYSGLMERRRPFVESLRPYGQALLLLPAFSYGHNTPVSLRAFYTLEDFGSNS
PLPVFLNPDYLRRLSKFWRERGLNSVRPSTGLIVASLALEICSNVHLYGFWPFSKHPYDSQA
ITNHYYDNRESKKNVHSMPTEFEYLLKLHNQGVIRIHLGKCQPTHS

>Pygnat_GT29_ST8Sia6

MRVLLLRLLAVLSLASFCSVLLWLFLSNSDAGLRGPRYTKKERADSIKPDSCKVCRENVIID
KALRVYSSRWRRQEANFKRFRSLLSRNCHALSKAVVTQANTPVGSKLVYDGEKTKPLQVTSA
LFNTFAKEQPFGNATWDTCAVVGNGGILANSSCGEKINSADFIIRCNLPPLGSGYEKDVGNQ
TSLVTANPSILIEKFGGLMERRRPFVESLRPYGDSLLVLPAFSYGHNTPVSLRAFYTLEDFG
ITSARPVFLNPEYLSSLARFWRGQGLRSARLSTGLIVASLALELCTNVHLYGFWPFSQHPYG
RQPITNHYYDDRQSKKNVHSMPAEFDHLLRLHMQGVIQMHLGACSMLNRTSN

>Onckis_GT29_ST8Sia6-A

MRGLFSFMLTLSILGTVLTALVWYFFSKHNVQPGRPPHKSHIMNSGPVPSRTCKGCRDTVTD
KVVELYSHSWKKQEDKFRNFRSLLSNRCNGLTKAMVTQANTPLGSKVVYDGEKRKPLQVTPE
LFSTFAKENPFVNTTWDTCSVVGNGGILANSSCGEKIDSAQFVIRCNLPPLENGYERDVGNK
TDLVTANPSILMEKYGGLQERRRPFVESLRSYGDSLMLLPAFSYGHNTPVSLRALYTIQDFD
SPSRPVFLNPEYLQSLARFWQGQGLRTVRLSTGLIVASLALELCANVHLYGFWPFNEHPHRH
QPLTNHYYDDRQSKKTVHAMPAEFDHLLRLHTQGVLRIHLGECAPTARCRPSSPLILWVFIP
VLYLSVCASSSCLFKSTSVLSQCLLFP

>Esoluc_GT29_ST8Sia6

MRGGLLKLLFSLMVILLILGTILTALLGYIFNNSVGPGSPLHQSQGNSGSDTSGTCKGCRDT
TIIEKVVEHYSHSWKKQEDNFRKFRSLLRNTCHGLTKAVVTQSNTPLGSKVVYDGEKRKPLQ
VTAELFSTFAKEHPFVNATWDTCSVVGNGGILANSSCGERIDSAQFIIRCNLPPLANGYERD
VGNKTDLVTANPSILQEKYKGLQEHRLPFVKSLHSYGDSFVLLPAFSYGHNTPLSLRALYTI
QDFNSPSRPIFLNPEYLRSLARFWRSQGLKTARLSTGLIVASLALELCANVHLYGFWPFSQH
PQHHRPLTNHYYDDRQSKKTVHAMPAEFDQLQRLHNQGVLRLHLGECAPAAT

>Salsal_GT29_ST8Sia6

MRGLFSFMLTLSILGTVMTALVWYVFSNRNVQPGRLRHKSNIMNSGPVPSRTCKGCRDTVTE
KVVERYSHSWKKQEDKFRNFRSLLSNRCHGLTKAMVTQANTPLGSKVVYDGEKRKPLQVTPE
LFSTFAKENPFVNTTWDTCSVVGNGGILANSSCGEKIDSAQFVIRCNLPPLENGYERDVGNK
TDLVTANPSILMEKYGGLQERRRPFVESLRSYGDSLMLLPAFSYGHNTPVSLRALYTIQDFD
SPSRPVFLNPEYLQSLARFWQGQGLRTVRLSTGLIVASLALELCANVHLYGFWPFNKHPHRH
QNLTNHYYDDRQSKKTVHAMPAEFDHLLRLHTQGVLRIHLGECTPTASPGCSSKMAWASASV
SGFSLPVGSLGSAMPTLVQHNTTSQTQKPWAGPNINVSGPSGLGQGASEDPIQGSDSEQRTK
HRRGNRRRPGK

>Astmex_GT29_ST8Sia6

MRLLLLRLLGVLFITSFFSVVLWHFLSNSAAGLHGPRYSERKAAGPIKPTTSRAVKDNAIID
RLLKSYSPRWKRREANFSKFRSLLSSSCHAVSKAVVTQSNTPVGSKVVYDGEKTKPLQVTKA
LFSTFAKEQPFGNASWDTCAVVGNGGILVNSSCAEKINSANLVIRCNLPPLGNGYEKDVGNK
TSLVTANPSILIEKFSGLMERRRPFVESLRPYGDSLLLLPAFSYSHNTPVSLRALYALEDFN
AVGPRPVFFNPEYLSSLARFWRGRGLRTARLSTGLIVTSLALELCTNVHLYGFWPFSLHPHG
RQTITNHYYDNRQSKKNVHSMPSEFEHLLRLHVSGVIQLHLGECSTTDLNHTEKT

>Salalp_GT29_ST8Sia6

MRGLFCFLLTLSILGTVLTALVWYVFRDRNVQPGRPPHKDPIMNSGPVPSGTCKGCRDTVTE
KVVERYSHSWKKQEDKFRNFRSLLSNRCHGLTKAMVTQANTPLGSKVVYDGEKRKPLQVTPE
LFSTFAKENPFVNTTWDTCSVVGNGGILANSSCGERIDSAQFVIRCNLPPLENGYERDVGNK
TDLVTANPSILMEKYGGLQERRRPFVESLRSYGDSLMLLPAFSYGPNTPVSLRALYTIQDFD
SPLRPVFLNPEYLQSLAHFWQGQGLRTVRLSTGLIVASLALELCANVHLYGFWPFNEHPHRH
QPLTNHYYDNRQSKKTVHAMPAEFDHLLRLHSQGVLRIHLGECAPAAR

>Cluhar_GT29_ST8Sia6

MKLVLLKLLLSLMLIVVALGSFFTLLLWIVSSESDTTPRSPFHVKKSPDLTDCTGCRDSVID
KVLERYSKNWKKQENNYKRFRALLSSRCHGATKAVVTQANTPLGSKVVYDGEKRKPLQVTSA
LFNVFPKEPPFGNTSWDTCSVVGNGGILANSSCGRRIDSAQFVIRCNLPPLEHGYEEDVGNK
TDLVTANPSILHEKFGGLMERRRPFVEGLRPYGNSLILLPAFSYSHNTPVSLRAVYTLEDFR
SPARPVFLSPDYLTSLARFWRSQGLRSVRLSTGLIVASLALELCTNVDLYGFWPFSQHPHGH
QPLTNHYYDNRETKKKIHAMPAEFDHLLRLHGQGILRVHLGQCPRSDG

>Notfur_GT29_ST8Sia6

MRGHLLKPLLSLMFVGSLLTMLIWYTLDNNNVDPNPQQAKTEPQSSASCKDCREDIKKALEL
YSQTWKKQEDSYQNFRSLLNRKCKCFDKAIITQNNTPLGSKLVYDGERKRTLQVNQEIFNTF
PKGHPFSNKTLHTCAVVGNGGILANSSCGKTIDSAEFVIRCNLPPLSNGYEKHVGIKTHLVT
ANPSILMEKYAALMARRRPFVENLRSYGDSMLLLPAFSYGRNTPVSLRAFYTLEDFESPIQS
IFFNPAYLRNLAAFWRSQGLKAVRLSTGIIMTSLALEICENVHLYGFWPFGVHPYSSQDLTN
HYYDDRKTKIKFHAMPDEFNLLLNLHSQGVLKMHLGDCEPDEKRFHRSD

>Serlaldor_GT29_ST8Sia6

MRGQLLKTLFSLMITLLFVGSLLTTFIWFMFNDNNVEPHRTNPQKKSAPQSSDPCKGCREII
DNVIERYSQTWKKQEDNYQKFRSQLNSKCRGFNKAIITQANTPVGSKLVYDGEKRRSLQVTP
EIFSTFVKEHPFSNKTWGTCAVVGNGGILTNSSCGQTIDSAQFVIRCNLPPLKNGYEKHVGN
KTDLVTANPSILVEKYGALMAHRRPFVDSLHTYGNSLLLLPAFSFGHNTPVSLRAAYTIEDF
GSPIKPVFFNPEYLQRLAVFWRSQGLRAVRLSTGIIMASLALELCADVHLYGFWPFSTHPHG
FHPLTNHYYDDRKTKMKFHAMPAEFDLLLRLHSQGVLRLHLGDCRPHEK

>Larcro_GT29_ST8Sia6

MRGLLKSLFSLAITLLCLGSLLTTFIWYMFDHNNVEPRKPHPQKKSTPQSSEICKGCREVID
KVMERYSQSWERQEDNYQKFRAQLNNKCHGFDKAIITQANTPVGSKLAYDGEKKRILQVTPE
IFSTFAKERPFPNKTWDTCAVVGNGGILTNSGCGKVIDSAQFVIRCNLPPLENGYEKHVGIK
TDLVTANPSIFLEKYGGLMGRRRPFVESLHSYGNSLLLIPAFSYGHNTPVSLRVAYSLEDFK
SSTRPIFFNPEYLHSLALFWRSQGLRAVRLSTGIIMASLALELCANVDLYGFWPFSNHPHGF
HTLTNHYYDDRQTKVKFHAMPAEFELLLRLHSQGVLRLHLGDCQPSEK

>Paroli_GT29_ST8Sia6

MRGQLLKSLVSMMITLLFVGSLLTTLIWYVFNDNNVQPQELHPQKKSAPEPVDPCKGCREII
DKVIQRYSQTWKRQEDNYQKIRSQLSSKCHGFDKAIITQANTPVGSKLVYDGEKKRTLQVTP
EVFSTFIKERPFSNKTWDTCAVVGNGGILTNSSCGQMIDSAQFVIRCNLPPLENGYEKHVGT
KTDLVTANPSILMEKYGALMGRRRPFVESLRIYGDSLLLIPAFSYGHNTPVSLRVVYTIEDF
ESPTRPIFFNPEYLQSLAVFWRSQGLKAVRLSTGIIMASLALELCSNVHLYGFWPFSNHPHG
LHVLNNHYYDDRKTKTKFHAMPAEFDLLLRLHSQGALRLHLGDC

>Ampoce_GT29_ST8Sia6

MRGQLLKSLFSLMVTLLFLGSLFTSLIWFMFISNNVEPHRPPQKKNAPQHSDPCKGCREVID
KVVARYSQPWKRQEDSYQKFRFQLSSKCHGFNKAIITKTNTPVGSKLVYDGEKKRTLQVTPE
IFSTFIKDHPFSNKTWDTCAVVGNGGILTNSSCGKTIDSAQFVIRCNLPPLANGYEKHVGVK
TDLVTANPSILLEKYGALMGRRRPFVESLRTYGDSLLLLPAFSFGHNTPVSLRAAYTIEDFE
SPTRPVFFNPEYLHSLALFWRSQGLKAVRLSTGLIMASLAVELCSNVHLYGFWPFSNHPHGL
QPLTNHYYDNRQTKTKFHAMPAEFDLLLRLHSQGVLRLHLGDCPTSGK

>Angang_GT29_ST8Sia6

MRGLLLKLLFSLMLCLFILGTVLTAFVWYVFSYSDVKPRSSPSQVRKSPESDTSACKDCREY
TIDKMVELYSYAWKKQNANFNKFRSQLSSRCRGVSTAIVTQNNTPLDSKIYYDGEKRKPLQV
TPKLYSTFAKEQPFENVTWKTCAVVGNGGILVNSSCGEAIDSAHFVFRCNLPPLDKAYQKDV
GNKTNLVTANPSILIEKFEGLMEYRRPFVESLSDYGEALLLLPAFSYAHNTPVSLRAFYTLR
DFGGRARPAFLSPAYLQSLAHFWRAQGLRTVRLSTGLIVASLALELCANVHLYGFWPHAQHP
HDRRPLTNHYYDDRQGKKKVHAMPAEFGHLLRLHRQGVVRVHLGQCEDRPR

>Orenil_GT29_ST8Sia6-B

MRGQLSKSLMITVLFLGSLMTTLIWYMSVGKHVEPQRPASQKKHAPKPSEICKGCKEIISKV
QERYNQTWKKQEDNYLKFRTNLSVKCNGFDKAIITKNNTPVGLKLVYDGEKKRTLQVNKDFF
NIFTKENPFSNKKWDTCSVVGNGGILSESSCGKMIDSADFVIRCNLPPLENGYEKDVGIKTS

LVTANPSIFTQRYGSLVGRRLPFVESLHKYGNSLLLLPAFSFGINTAVCQRVAYTIEDFKSP
IRPVFFNPQYLDSLAQFWRSEGLKERRLSTGLIMASMALELCENVHLYGFWPFSNHPYGFYT
LTNHYYDDKPAKTSFHAMPAEFDRLLQLHTEGVLRLHLEDCK

>Litcat_GT29_ST8Sia6

MRRSGLILLGTSLLLVLITVWLAIGEAPRYRRIQAVLDAKHHFGLHLSIRPTQQDKSEGSTH
LSHQFIWSGKKPGNITTRVGLVKPEVSHIIPAHNYTASFFCRDVQDRLLSVSLRKRYSEDYY
IQAVNDVQNCTWKKKPQEYSRFRLYLSSCCNAVNNFIVSQNNTSLGSNITYEVESKKNILIA
EDIYKMLPKSQPFDGIPFKQCAVVGNGGILKNSSCGAEIDQSDFVFRCNLPPIWGNVSIDVG
NKTDLVTVNPSIIALKYGKLNDMKTAFLKNLTNYGYSFLLLPAFSYSSNTAISFEVHHLLRK
YQAKQKAIFFHPNYLKSLAQFWKGRGVRAYRLSTGFMITSAAIELCQDVTLYGFWPFSKNLD
GKPISHHYYDNQLPKPGFHAMPKEFYQVLQLHHKGVLKLQLGECEKR

>Onctsh_GT29_ST8Sia6

MRGLFSFMLTLSILGTVLTALVWYFFSKHNVQPGRTPHKSHIMNSGPVPSRTCKGCRDTVTD
KVVELYSHSWRKQEDKFRNFRSLLSNRCHGLTKAMVTQANTPLGSKVVYDGEKRKPLQVTPE
LFSTFAKENPFVNTTWDTCSVVGNGGILANSSCGEKIDSAQFVIRCNLPPLENGYERDVGNK
TDLVTANPSILMEKYGGLQERRRPFVESLRSYGDSLMLLPAFSYGHNTPVSLRALYTIQDFD
SPSRPVFLNPEYLQSLARFWQGQGLRTVRLSTGLIVASLALELCANVHLYGFWPFNEHPHRH
QPLTNHYYDDRQSKKTVHAMPAEFDHLLRLHTQGVLRIHLGECAPTASPGCSSKMAWAPASV
SGLSLPVGSLGSAMPTLVQRNTTSQSQKPWAGPDTNVSGPSGLGQGASEDPTQGSEQRTKHR
TGNRRRPGK

>Hetzeb_GT29_ST8Sia6

MAASKCTLGIILLSCGIFILCAIFVVNFEQPSNYRLQGNDTERKGIHYSGKTAGDRGCDKCT
YSQVQNFWPLQQKNFTACERIYWEIASSPIQWKTEKDVIKSVAKLQKCQWHKQEVAAEELRS
ELKRCCHAAYNYLVTQKNMPVGTELIYDAESKNKINITPQIFSIFPKNSFFSGRQYRRCAVI
GNGGILANSSCGAEIDQADFVFRCNLPPMGGEFTRDIGTKTNLVTANPSIILERYAKLQDRR
RSFFTNLAVYDEALLLLPAFSYSKNTDLCFRALYTLQDFQAKQKVVFFNPVYLKNLANFWLS
KGIQVKRLSTGIMIVSVAVELCEEVWIYGFWPFGKNVEGKVVSHHYFDNLLPKPDTHSMPLE
FHHLLQMHSKGMIKLQMTQCQADPK

>Squaca_GT29_ST8Sia6

MASICALRIMVLSCGICIFLCGILVVNFEEPLNYRYMVQLSKLKLNYVFGSNLQVNDSEGKR
INDSEKTGGDQDCEKCTYRQTEEEVIKTVAQLQQCQWHKQELEAEKFRSELKRCCYAAYDGL
VTQRNTPVGTELTYDAEPKKKIKITHPIFTIFPKDSAFSSRQYRRCAVIGNGGILANSSCGA
EIDQADFVFRCNLPPVGGDFAQDVGTKTNLVTANPSIIAERYAELHDRRKPFFTNLTVYNEA
LLLLPAFSYTKNTVLCFRALYTLQDFQAKQKVVFFNPVYLKHLTEFWLSKGIQVNRLSTGIM
VVSVAIELCEEVWIYGFWPFGRNVEGRVMSHHYFDNLLPKPDVHSMPSEFHQLLQMHSKGII
KLQTAQCQADPKEMGNI

>Ambmex_GT29_ST8Sia6

MGARGMKMFTLLVFLSTCLLLLSFWITSGELPTSRDKQTKERNIFTNNSQPGHSQQLKRNGG
ATTSKPDHHTSRSNLRSANSSVAFRFLKQDKLYLMQTNAQSNSVANVQLCKETQDRYLSATL
RKRYSEDFYIQSFRKVQNCTWTRRPEEYYKFRSKLASCCNAVNNFIVSQNNTSLGSNMSYEV
DTKKNIVISETIFKMLPQSQPFVERSYKHCAVVGNGGILQNSSCGAEIDESDFVFRCNLPPV
NGDFHKDVGNKTNVVTVNPSIIALRYGKLSQKKTVFLQNITKYGDAYFLLPAFSYRSNTAVC
FKVFNALKEAKANQKAIFFHPKYLKNLGQFWRANGVRAYRLSTGLMIASAAIELCDHVKLYG
FWPFSKNTEGNLISHHYYDNQLPKPGFHAMPKEFIQYLQLHNKGILKLQVGECENKTKI

>Rhimar_GT29_ST8Sia6

MKRSAGILLLTCLLLLLLTGWLTIRDPPHYRPGNITTRVGLVKPEVNHLISTPNNYTTDLFC
RDIPDRLLTVSLRKRYSEDCYIQAVNEVQNCTWQKKPQEYSRFRFYLSSCCNAVNNFIVSQN
NTSLGSNITYEVESKKNILIAEDIYKMLPKSQPFEGIPFKQCAVVGNGGILTNSSCGAEIDR
SDFVFRCNLPPIWGNAAVDVGNKTDLVTVNPSIIALKYGKLNEMKTAFLRNLTSYGGSFLLL
PAFSYSSNTAISFEVHNLLKKYQAKQRAIFFHPNYLKSLAQFWRGRGVRAYRLSTGFMITSA
AIELCEDVKLYGFWPFSKNPNGKPISHHYYDNQLPKPGFHAMPKEFYQVLQLHYKGVLKLQI
GECQKR

>Latmen_GT29_ST8Sia6

MWMCRMRKCILLWILLASGVFFLLTFWIKYDAALHYREIANKDWRDCLATACGGRRGMRELE
SAVSPHRPLPTSNSSNTNQNGSTFNKLNLLKADSSSCKELHNRMSATTPRKRYSDDYYIQTV
NELQKCSWVIRPEEHEKFRSELSVCCNAVRNFIVSQNNTPLGTNMSYEVENGRTFLITQRIF
KMFPQSQPFAGYPYNQCAVVGNGGILKNSACGTEIDQADFVFRCNLPPTLGNISIDVGSKTS
LVTLNPSIITHRFGKLNEKRKPFVETVSHYGEAFLLLPAFSFRSNTALSFKVYHTLEAFRGK
QKTIFFHPKYLKSLALFWRSKGIKVYRLSSGFMIANAARRLCKEVRLYGFWPFSKNNEGKNI
SHHYYDNQLPKPGIHSMPKEFYHFLKLHNKGIIKLQFGRCDIT

>Homsap_GT29_ST8Sia1

MSPCGRARRQTSRGAMAVLAWKFPRTRLPMGASALCVVVLCWLYIFPVYRLPNEKEIVQGVL
QQGTAWRRNQTAARAFRKQMEDCCDPAHLFAMTKMNSPMGKSMWYDGEFLYSFTIDNSTYSL
FPQATPFQLPLKKCAVVGNGGILKKSGCGRQIDEANFVMRCNLPPLSSEYTKDVGSKSQLVT
ANPSIIRQRFQNLLWSRKTFVDNMKIYNHSYIYMPAFSMKTGTEPSLRVYYTLSDVGANQTV
LFANPNFLRSIGKFWKSRGIHAKRLSTGLFLVSAALGLCEEVAIYGFWPFSVNMHEQPISHH
YYDNVLPFSGFHAMPEEFLQLWYLHKIGALRMQLDPCEDTSLQPTS

>Musmus_GT29_ST8Sia1

MSPCGRALHTSRGAMAMLARKFPRTRLPVGASALCVVVLCWLYIFPVYRPPNEKEIVQGVLA
QSTAWRTNQTSASLFRRQMEDCCDPAHLFAMTKMNSPMGKSLWYDGELLYSFTIDNSTYSLF
PQATPFQLPLKKCAVVGNGGILKMSGCGRQIDEANFVMRCNLPPLSSEYTRDVGSKTQLVTA
NPSIIRQRFENLLWSRKKFVDNMKIYNHSYIYMPAFSMKTGTEPSLRVYYTLKDVGANQTVL
FANPNFLRNIGKFWKSRGIHAKRLSTGLFLVSAALGLCEEVSIYGSWPFSVNMQGDPISHHY
YDNVLPFTGYHAMPEEFLQLWYLHKIGALRMQLDPCEEPSPQPTS

>Ratnor_GT29_ST8Sia1

MSPGGGPLHTSRGAMAMLARKFPRTRLPVGASALCVVVLCWLYVFPVYRLPNEKEIVQGVLA
QRTAWRRNQTSARLFRKQMEDCCNPAHLFAMTKVNSPMGKSLWYDGEFLYSLTIDTSTYSLF
PQATPFQLPLKKCAVVGNGGILKMSGCGRQIDEANFVMRCNLPPLSSEYTRDVGSKTQLVTA
NPSIIRQRFENLLWSRKKFVDNMKIYNHSYIYMPAFSMKTGTEPSLRVYYTLKDAGANQTVL
FANPNFLRNIGKFWKGRGIHAKRLSTGLFLVSAALGLCEEVSIYGFWPFSVNMQGEPISHHY
YDNVLPFSGFHAMPEEFLQLWYLHKMGALRMQLDPCENPPSPQPTS

>Macmul_GT29_ST8Sia1

MSPCGRARRQTSRGAMAVLAWKFPRTRLPMGASALCVVVLCWLYIFPVYRLPNEKEIVQGVL
QQGTAWRRNQTAARAFRKQMEDCCDPAHLFAMTKMNSPMGKSMWYDGEFLYSFTIDNSTYSL
FPQATPFQLPLKKCAVVGNGGILKKSGCGRQIDEANFVMRCNLPPLSSEYTKDVGSKSHLVT
ANPSIIRQRFQNLLWSRKTFVDNMKIYNHSYIYMPAFSMKTGTEPSLRVYYTLSDVGANQTV
LFANPNFLRSIGKFWKSRGIHAKRLSTGLFLVSAALGLCEEVAIYGFWPFSVNMHEQPISHH
YYDNVLPFSGFHAMPEEFLQLWYLHKIGALRMQLDPCEDTSLQPTS

>Bostau_GT29_ST8Sia1

MSPCGRARRHTSRGAMAVLAWKFPRTRLPVGASALCVVVLCWLYVFPVYRLPDEKEIVQGVL
QQGTAWRRNRTAAGIFRKQMEDCCDPAHLFAMTKMNAPMGKSLWYDGEFLYSFTIDNSTYSL
FPQATPFQLPLKKCAVVGNGGILKKSGCGRQIDEADFVMRCNLPPLSSEYTKDVGSKSHLVT
ANPSIIRQRFQNLLWSRKTFVDHMKVYNHSYIYMPAFSMKTGTEPSLRVYYTLSDVGANQTV
LFANPNFLRSIGKFWKSRGIHAKRLSTGLFLVSAALGLCEEVAIYGFWPFSVNMHEQPISHH
YYDNVLPFSGFHAMPEEFLQLWYLHKIGALRMQLDPCEDNSLQPTS

>Canlupfam_GT29_ST8Sia1

MSPCGRARRHTSRGAMAILAWKFPRTRLPVGASALCVVVLCWLYIFPVYRLPNEKEIVQGVL
QQGTAWRRNQTAARVFRKQMEDCCDPAHLFAMTKLNSPMGKSMWYDGEFLYSFTIDNSTYSL
FPQATPFQLPLKKCAVVGNGGILKKSGCGRQIDEANFVMRCNLPPLSSEYTKDVGSKSHLVT
ANPSIIRQRFQNLLWSRKTFVDNMKIYNHSYIYMPAFSMKTGTEPSLRVYYTLSDVGANQTV
LFANPNFLRSIGKFWKSRGIHAKRLSTGLFLVSAALGLCEEVAIYGFWPFSVNMHEQPISHH
YYDNVLPFSGFHAMPEEFLQLWYLHKIGALRMQLDPCEDTSLQPTS

>Galgal_GT29_ST8Sia1

MAGLAWKWPRTRLPVGASALGVFVLCWLYIFPVYRLPDEKEIVQGVLMQQGKAWRRNHTAVA
VFRKLLEDCCDPGQLFAMTKQNSPMGKNLWFDGEFLYSFTIDNTTYSLFPQATPLQLPLKKC
SVVGNGGILKKSGCGKQIDQADFVMRCNLPPLSREYSKDVGLKTQLVTANPSIIQKRFQNLL
WSRKAFVDSVKVYNHSYIYMPAFSMKTGTGPSLRVYYTLKDFGAKQAVLFANPNFLRDIGKF
WKNKGIHAKRLSTGLFLVSAALGLCEEVTIYGFWPFSVDLHGKFISHHYYDNVLPDSGFHAM
PEEFLQLWFLHKSGVLRMQLEQCEDPLTQSSA

>Xenlae_GT29_ST8Sia1

MWGRWRGAGGRRGVAQPVIPQMKLLGGRVPLGASALGLLIVCWFYIFPGGERLPGHKEMIRQ
VLQFGPRWGRNRSSGDSFRKLLQDCCDPPRLFSMTKANTALGENLWYDGEFFQSLTIDNTTR
SLFPQDTPIKLPLKRCSVVGNGGILKNSRCGEQIDEADFVMRCNLPPLSREYTDDVGTKTQL

VTVNPSIIDKRFQNLLWSRKSFVESVSVYKQSYVYMPAFSTKRGTDPSLRVYYTLEDFGTNQ
TVLFANPNFLRNVGKFWKSKGVHSKRLSTGLFMVSAALSLCEEVTIYGFWPFQMDLGGRHIS
HHYYDNMLPLSGVHAMPEEFLQLWHLHKSGVLQMQLDQCKKDVSSKKPH

>Siltro_GT29_ST8Sia1

MKLQGSRMWLCPRTRLPVGASALGFLILCWLYVFPGYRLPGHKEMVREVLRFGPGWRKNRTE
MDSFRKLLQDCCDPPHLFSLTKVNTPLGENLWFDGEFFHSLTIDNSTRSLFPQDTPFKLPLK
RCSVVGNGGILKNSRCGEQIDEADFVMRCNLPPLSREYTEDVGTRTQLVTVNPSIIDKRYQN
LLWSRKSFVENLRVYQQSYVYMPAFSTKRGTDPSLRVYYTLADFGTNQTVLFANPNFLRNVG
KFWKSRGIHSKRLSTGLFMVSAALSLCEEVTIYGFWPFQMDLGGRYISHHYYDNTLPLSGVH
AMPEEFLQLWLLHKSGVLQMQLDQCKKDVSSQKPH

>Takrub_GT29_ST8Sia1

MLARCCRGKLSVWAGVCVLVLCWFYIFPGYRLPADKEIVDEVLRQGEAWQKNQTGIDLYRKL
LTECCDPPRMFAVTKQNSPLGKVMWYDGEFYHSHTVNNETYPLFVQENPLQLPLKKCAVVGN
GGILRGSKCGKDIDRADFIMRCNLPPLSKEYLEDVGTKTHLVTANPSIIENRFQNLLWSRKH
FVDSMKVYGTSYIYMPAFSMSPGTNPSLRAYYALADASANLTMLFANPEFLRSVGKFWKARN
VHAKRLSTGLFMVSLALALCKEVTVYGFWPFAIDLDEQPVSHHYYDNILPYKWFHTMPEEFV
QLWHLHKSGTLRMRVGRCPPEDGGR

>Tetnig_GT29_ST8Sia1

MLARCCRGKVSVWAGVCVLVLCWFYIFPGYRLPGDKEIVAEVLRQGEAWHRNQTGIDLYRRL
LTDCCDPPKMFAVTKRNSPLGKVMWYDGEFYHSHTVNNETYRLFVQDNPLQLPLKKCAVVGN
GGILRASKCGKDIDQADFIMRCNLPPLSEEYLEDVGTKTHLVTANPSIIENRFQSLLWSRKH
FVDSIKVYGTSYIYMPAFSMRPGTEPSLRAYYALADTSSNLTVLFANPEFLRTVGKFWKARN
VHAKRLSTGLFMVSLALALCEEVTVYGFWPFAIDLDEQPVSHHYYDNILPYKWFHTMPEEFV
QLWHLHKSGTLRMRVGRCSSQGGGR

>Gasacu_GT29_ST8Sia1

MLVRCYRGKLSVWAAMCVLVLCWFYVFPVYRLPRDKDIVEEVLKQGGVWQKNQTGIDLYRKL
LTECCDPRRMFAVTKENSPTGKVLWYDGEFYHSHTVNNETYPLLVKDNPLRLPLKKCAVVGN
GGILRHSQCGRDIDQADFVLRCNLPPLSKEYVDDVGTRTHLVTANPSIIEKRFQNLLWSRKV
FVDSMKVYGSGYIYMPAFSMKTGTDPSLRAYHALADSSSNLTMLFANPEFLRTVGRFWKARG
VHARRLSTGLFLVSLALGLCEEVTAYGFWPFSVGLDEQPVSHHYYDNILPYTWFHAMPEEFV
QLWHLHKSGALRVRVGRCSAQGGGG

>Danrer_GT29_ST8Sia1

MVSLRCHRSKYIWATLGVLALLWLYIFPVYRIPSDKEMVEEVLRQGQTWSRNQTAVELYRKL
LTDCCNPKRMFAVTKENSPLGKVLWYDGEFYHYHTVTNETYPIFVQDTPLQLPLKRCSVVGN
GGVLKHSGCGNEIDRADFIMRCNLPPLSKDYTDDVGTKTHLVSANPSIIEKSFQNLLWSRKS
FVESMKAYGSSYIYIPAFSMKPGTDPSLRAYHALADSSSNQTVLFANPDFLKNVGIFWKNHG
VHGKRLSTGLFLVSLALGLCEEVTAYGFWPFSVGLDERPVSHHYYDNILPSSRFHAMPEEFL
QLWHLHKSGTLRMRVGSCAKERMELKREK

>Anocar_GT29_ST8Sia1

GPRTRLPVGTNALGFFFVLCWLYVFSVYHLPGEKEIMCGMRLQQKCKAWQRGHHAAHAFRKL
LKECCDPGQLFAMTKENSPVGKNLWYDGEFLYSFTIDNETFSLFPKATPFQLPLKKCSVVGN
GGILKKSNCGRQIDQADFVMRCNLPPLTSEYSKDVGSKTQLVTANPSIIKKRFQNLLWSRKA
FVDSVKVYNQSYIYMPAFSMKAGTEPSLRVYYTLADVGARQTVIFANPNFLRDIGKFWKSKG
IHGKRLSTGLFLVSAALGMCEEVTIYGFWPFSMDLHGNFISHHYYDNILPDSGFHAMPEEFL
QLWFLHKSGVLRMQLEHCEDSIFHPAS

>Calmil_GT29_ST8Sia1

MNRDSALPPRSVFCFTTMWSIWKCYRSRLPVGAALCVFVLCWLYVFPVYRIPDQKEIVNDVL
HQPVWRRNQTATTAFRRLLENCCETKRMFAVTQHNSPMGKNLWFDGEFLHSLSVNNEMFAMF
PQDTPFQIPLKKCSVVGNGGILKRSACGDKIDKADFVMRCNLPPLSNEYARDVGHRTQLVTA
NPSIIKKRYQNLMWSRKAFVESMKVYGRSYIYMPGFSMKAGTEPSFRTYYTLSDVNANQTII
FANPDFLRNVQKFWKGKGVHAKRLSTGLFLVSVALGLCEEVTLYGFWPFSTDLEERTISHHY
YDNVMPDTSYHAMPEEFLQLWLLHKSGILKIHVGKCGQIPM

>Pantro_GT29_ST8Sia1

MSPCGRARRQTSRGAMAVLAWKFPRTRLPMGASALCVVVLCWLYIFPVYRLPNEKEIVQGVL
QQGTAWRRNQTAARAFRKQMEDCCDPAHLFAMTKMNSPMGKSMWYDGEFLYSFTIDNSTYSL
FPQATPFQLPLKKCAVVGNGGILKKSGCGRQIDEANFVMRCNLPPLSSEYTKDVGSKSQLVT
ANPSIIRQRFQNLLWSRKTFVDNMKIYNHSYIYMPAFSMKTGTEPSLRVYYTLSDVGANQTV
LFANPNFLRSIGKFWKSRGIHAKRLSTGLFLVSAALGLCEEVAIYGFWPFSVNMHEQPISHH
YYDNVLPFSGFHAMPEEFLQLWYLHKIGALRMQLERCEDTSLQPTS

>Orylat_GT29_ST8Sia1

MLARCYRAKLSVWAALCVLVLCWFYVFPGYRLPRDKDIVAEVLRQGDVWRKNQTGIDLYRDL
LTKCCNPKKMFAVTKENSPIGKVLWYDGEIYHSHTVNSETYQLFIKRRGAASSCRRCAVVGN
GGILKGSKKGKEIDHHHYIFRCNLPPLSREYVEDVGTKTHLVTANPSIIEKRFQNLVWSRKT
FVDSMKVYGSSYIYMPAFSMAPGTDPSLRAYFALTDVPSNLTMLFANPDFLLSVSKFWKAHG
VHARRLSTGLFLVSLAMGLCDEVTTYGFWPFSVDLDEQPISHHYYDNILPYKWFHAMPEEFV
QLWHLHKSGILRMKVGHCSPQN

>Homsap_GT29_ST8Sia3

MRNCKMARVASVLGLVMLSVALLILSLISYVSLKKENIFTTPKYASPGAPRMYMFHAGFRSQ
FALKFLDPSFVPITNSLTQELQEKPSKWKFNRTAFLHQRQEILQHVDVIKNFSLTKNSVRIG
QLMHYDYSSHKYVFSISNNFRSLLPDVSPIMNKHYNICAVVGNSGILTFIQCGREIDKSDFV
FRCNFAPSEAFQRDVGRKTNLTTFNPSILEKYYNNLLTIQDRNNFFLSLKKLDGAILWIPAF
FFHTSATVTRTLVDFFVEHRGQLKVQLAWPGNIMQHVNRYWKNKHLSPKRLSTGILMYTLAS
AICEEIHLYGFWPFGFDPNTREDLPYHYYDKKGTKFTTKWQESHQLPAEFQLLYRMHGEGLT
KLTLSHCA

>Pantro_GT29_ST8Sia3

MRNCKMARVASVLGLVMLSVALLNLSLISYVSLKKENIFATPKYASPGAPRMYMFHAGYRSQ
FALKFLDPSFVPITNSLTQELQEKPSKWTFNRTAFLHQRQEILQHVDVIKNFSLTKNSVRIG
QLMHYDYSSHKYVFSISNNFRSLLPDVSPIMNKHYNICAVVGNSGILTGSQCGQEIDKSDFV
FRCNFAPTEAFQRDVGRKTNLTTFNPSILEKYYNNLLTIQDRNNFFLSLKKLDGAILWIPAF
FFHTSATVTRTLVDFFVEHRGQLKVQLAWPGNIMQHVNRYWKNKHLSPKRLSTGILMYTLAS
AICEEIHLYGFWPFGFDPNTREDLPYHYYDKKGTKFTTKWQESHQLPAEFQLLYRMHGEGLT
KLTLSHCA

>Musmus_GT29_ST8Sia3

MRNCKMARVASVLGLVMLSVALLILSLISYVSLKKENIFTTPKYASPGAPRMYMFHAGFRSQ
FALKFLDQSFVPITNSLTHELQEKPSKWTFNRTAFLHQRQEILQHVDVIKNFSLTKSSVRIG
QLMHYDYSSHKYVFSISNNFRSLLPDVSPIMNKRYNVCAVVGNSGILTGSQCGQEIDKSDFV
SRCNFAPTEAFHKDVGRKTNLTTFNPSILEKYYNNLLTIQDRNNFFLSLKKLDGAILWIPAF
FFHTSATVTRTLVDFFVEHRGQLKVQLAWPGNIMQHVNRYWKNKHLSPKRLSTGILMYTLAS
AICEEIHLYGFWPFGFDPNTREDLPYHYYDKKGTKFTTKWQESHQLPAEFQLLYRMHGEGLT
KLTLSHCA

>Ratnor_GT29_ST8Sia3

MRNCKMARVASILGLVMLSVALLILSLISYVSLKKENIFTTPKYASPGAPRMYMFHAGFRSQ
FALKFLEPSFVPITNFLTHELQEKPSKWTFNRTAFLHQRQEILQHVDVIKNFSLTKNSVRIG
QLIHYDYSSHKYVFSISNNFRSLLPDVSPILNKRYNICAVVGNSGILTGSQCGQEIDKSDFV
FRCNFAPTEAFHKDVGKKTNLTTFNPSILEKYYNNLLTIQDRNNFFLSLKKLDGAILWIPAF
FFHTSATVTRTLVDFFVEHRGQLKVQLAWPGNIMQHVNRYWKNKHLSPKRLSTGFLMYTLAS
AICEEIHLYGFWPFGFDPNTREDLPYHYYDKKGTKFTTKWQESHQLPAEFQLLYRMHGEGLT
KLTLSHCA

>Bostau_GT29_ST8Sia3

MRNCKMARVASVLGLIMLSVALLILSLISYVSLKKESIFTTPKYANPGAPRMYMLHAGFPNL
RLEKLLDSSLVPFPHSVTHELQARPKWTFNRTAFLHQRQEILQHVDVIKNFSLTKNSVRIGQ
LMHYDYSSHKYVFSISNNFRSLLPDVSPIVNKRYNICAVVGNSGILTGSRCGPQIDKSDFVF
RCNFAPTEAFQRDVGRKTNLTTFNPSILEKYYNNLLTIQDRNNFFLSLKKLDGAILWIPAFF
FHTSATVTRTLVDFFVEHRGQLKLQLAWPGNIMQHVNRYWKNKHLSPKRLSTGILMYTLASA
VCEEIHLYGFWPFGFDPNTREDLPYHYYDKKGTKFTTKWQESHQLPAEFQLLYRMHGEGLTK
LTLSHCA

>Canlupfam_GT29_ST8Sia3

MSHYKMARVASVLGLVMLSVALLILSLISYVSLKKENIFTTPKYANPGAPRMYMFHAGFRSQ
FALKFLDPSVPITNSLTHELQEKPSKWTFNRTAFLHQRQEILQHVDVIKNFSLTKNSVRIGQ
LMHYDYSSHKYVFSISNNFRSLLPDVSPIVNKHFNICAVVGNSGILTGSRCGQEIDKSDFVF
RCNFAPTEAFQRDVGRKTNLTTFNPSILEKYYNNLLTIQDRNNFFLSLKKLDGAILWIPAFF
FHTSATVTRTLVDFFVEHRGQLKVQLAWPGNIMQHVNRYWKNKHLSPKRLSTGILMYTLASA
ICEEIHLYGFWPFGFDPNTREDLPYHYYDKKGTKFTTKWQESHQLPAEFQLLYRMHGEGLTK
LTLSHCA

>Galgal_GT29_ST8Sia3

MVRVASVLGLVMLSIALLILSLISYVSLKKDNIFGAPRAAGPGGPRMYMFHAGFRSQFALKF
LDPSFVPITNSLGQELQEKPSKWVFNRTAFAQQRQEILQHVDVIKNFSLTKNSVRIGQLMHY
DYSSHKYVFSISNNFRSLLPDVSPILNKHYNVCAVVGNSGILTGSQCGQEIDKSDFVFRCNF
APTEAFQKDVGRKTNLTTFNPSILEKYYNNLLTIQDRNNFFLSLKKLDGAILWIPAFFFHTS
ATVTRTLVDFFVEHRGQLKVQLAWPGNIMQHVNRYWKNKHLSPKRLSTGILMYTLASAICEE
IHLYGFWPFGFDPNTREDLPYHYYDKKGTKFTTKWQESHQLPAEFQLLYRMHGEGLAKLTLS
RCA

>Siltro_GT29_ST8Sia3

MVRVASVLGLVMLSVALLILSLISYVSLKKENIFTTPKYASAGGPRMYMFHAGFRSQFAMKF
LDPSFVPITNSLTQELQEKPAKWVFNKTAYARQRREILQNVDVIRNFSLTKNSVRTGQLMHY
DYSSHKYVFSISNNFRSLLPDTSPVMNKRYNVCAVIGNSGILTESQCGAEIDKADFVFRCNF
APTEGFQKDVGRKTNLTTFNPSILEKYYNNLLTIQDRNNFFLSLKKLDGAILWIPAFFFHTS
ASVTRTLVDFFVEHRDQLKVQLDWPGNIMQHVNRYWKNKHLSPKRLSTGILMYTLASSVCEE
IHLYGFWPFGWDPNTGKDLPYHYYDKKGTKFTTKWQESHQLPAEFKLLYKMHREGLTKLTLS
QCA

>Takrub_GT29_ST8Sia3

MVRIASALGLVMFSVVLLILSLISYVSIRKDFLLGSPRYGGPRMSMFHSGFRSQLAMKYLDP
VFTPLTSSLSEDLQNASKWSFNSSAFAQLKNEISQYIDVPRNFTLTRDSVRIGQLMHYDYSS
HKYVFSIGENFRSLLPETSPILQKHYNVCAVVGNSGILTGSRCGQQIDRFDFVFRCNFAPTE
IFKKDVGRRTNMTTFNPSILEKYYNNLLTVQDRNNFFLSLKKLDTTILWIPAFFFHTSATVT
RTLVDFFVEHRGQLKVRLAWPGNIMQYINNYWKTKQLSPKRLSTGILMYTLASSMCDQIHLY
GFWPFGWDPNTGKELPYHYYDKKGTKFTTKWQESHQLPTEFKLLYKMHREGLLKLSLSHCA

>Takrub_GT29_ST8Sia3-r

MVRVAKALGLVILCVAVLILSLISYVSLRKDSFFSSKYYTGGPRIMFHAGFRSQLAVNSLNP
SFIPLTNALNEELHGKASKWKFNKTAFHQQRKDILGFIDIPNNFSLTKDRVRVGQLMHFDYS
SHKYVFSISNNFKSLLPDTSPIHNKHYSLCSVVGNSGILTGSHCGANIDQADFVFRCNFAPT
EVYSKDVGRKTNMTTFNPSILERYYNNLLTIQDRNNFFLNLKKLGGAILWIPAFFLHTSATV
TRTLVDFFVEHKGQLKVELAWPGNIMHDVNKYWKNKNLSPKRVSTGIFMYTLASAMCDEIHL
YGFWPFGWDPNTGKELPYHYYDKKGTKFTTKWQETHQLPTEFKLLYKLHREGVIRLSLTHCT

>Tetnig_GT29_ST8Sia3

MVRIASVLGLVMFSVVLLILSLITYVSIRKDFVPGRYGGGPRMSLLHAGFRSQLAVNSLNPS
FIPLTSALSEDLQNSSKWSHNGSAFTQLKKEISQYIDIPRNFTLTRDSVRIGQLMHYDYSSH
KYVFSIGENFRSLLPEVSPIRQKHYNVCAVVGNSGILTGSRCGQEIDSLDFVFRCNFAPTEL
FKKDVGRRTNMTTFNPSILEKYYNNLLTVQDRNNFFLSLKRLDPAILWIPAFFFHTSATVTR
TLVDFFVEPRGQLKVQLAWPGNIMQYINNYWKTKQLSPKRLSTGILMYTLASSMCERIHLYG
FWPFGWDPNTGRELPYHYYDKKGTKFTTKWQESHQLPTEFKLLYKMHREGVLKLSLSHCG

>Tetnig_GT29_ST8Sia3-r

MVRVAKALGLVILCVAVLILSLISYVSLRKDSFFSSKYYTGGPRIMFHAGFRSQLAVNSLNP
SFIPLTNALNEELHGKASKWKFNKTAFHQQRKDILGFIDIPNNFSLTKDRVRVGQLMHFDYS
SHKYVFSISNNFKSLLPDTSPIHNKHYSLCSVVGNSGILTGSHCGANIDQADFVFRCNFAPT
EVYSKDVGRKTNMTTFNPSILERYYNNLLTIQDRNNFFLNLKKLGGAILWIPAFFLHTSATV
TRTLVDFFVEHKGQLKVELAWPGNIMHDVNKYWKNKNLSPKRVSTGIFMYTLASAMCDEIHL
YGFWPFGWDPNTGKELPYHYYDKKGTKFTTKWQETHQLPTEFKLLYKLHREGVIRLSLTHCT

>Danrer_GT29_ST8Sia3

MVRVVSVLGLVMFSVALLILSLISYVSIKKDFIFTAPKYANAGGPRMYMFHAGFRSQLAMKF
LDPAFTSLNTALNENLQESSNWRFNRSAYAELNKEIAQHIDVPHNFTLTKNSVRVGQLMHYD
YSSHKYVFSIGENLRSLLPDASPVLNKRYNTCAVVGNSGILTGSRCGPEIDKYDFVFRCNFA
PTEVFRRDVGRRTNLTTFNPSILEKYYNNLLTIQDRNNFFLSLKKLDGAILWIPAFFFHTSA
TVTRTLVDFFVEHKGQLKVQLAWPGNIMQYVNKYWKTKQLSPKRLSTGILMFTLASSLCEQV
HLYGFWPFGWDPNTGKELPYHYYDKKGTKFTTKWQESHQLPTEFKLLFKMHADGVLKLSLSH
CA

>Anocar_GT29_ST8Sia3

MRSCKMVRVVSVLGLVMLSVALLILSFISYVSLKKDNIFTTPKYANTGVPRMYMFHAGFRSQ
FALKFLDPSFVPNTNSLNHELQDKSPKWIFNRTAFLQQRREILQHVDVIKNFSLTKNMVRIG
QLMHYDYSSHKYVFSISNSFRMLLPDVSPIQNKHYNICAVVGNSGILVGSQCGQEIDKYDFV
FRCNFAPTEAFHKDVGRKTNITTFNPSILEKYYNNLLTIQDRNNFFLSLKKLDGAILWIPAF
FFHTSATITRTLVDFFIEHRAQLKVQLAWPGNIMQHVNRYWKNKHLAPKRLSTGILMYTLAS
AICDEIHLYGFWPFGFNPNNREDLPYHYFDKKGTKFTTKWQESHQLPAEFQQLFRMHSEGLA
KLTLSHCA

>Orylat_GT29_ST8Sia3

MLRYTSTFGLIIFSVALLILSFISFVSIKKDVNPRYGPNGGPRMYMFHAGFRSQVAMKYLDP
AFTSLSSAINEDLKNSSKWTYNSTAFIQLRTEISHHIDMPHNFTLTRDAVKVGQLMHFDYSS
HKYVYSIGENFRSLLPEVSPILNRHYNVCAVVGNSGILTGSLCGAQIEKYDFVFRCNFAPTE
IFKKDVGRRTNMTTFNPSILEKYYNNLLTVQDRNNFFLSLKKLDGAILWIPAFFFHTSATVT
RTLVDFFVEHRGQLKVQLAWPGNIMQYINNYWKTKQLSPKRLSTGILMYTLASSMCDQIHLY
GFWPFGWDPNTGKELPYHYYDRKGTKFTTKWQESHQLPAEFKLLHKMHTEGLLKLTLSHCA

>Orylat_GT29_ST8Sia3-r

MVRIAKALGLGILCAVVLILSLISYVSLRRDSLFSSVGGPRIMFHAGFRSQSAMNFLDPSFI
PLTTALNEELQGKPYKWKFNRTAFYQQRKDIFSYIDIPNNFSLTKNSVRVGQLMHFDYSSHK
YVFSISNNFKSLLPEISPILNKHYSVCAVVGNSGILTGSHCGPEIDQADFVFRCNFAPTDVY
SKDVGRKTNMTTFNPSILERYYNNLLTIQDRNNFFLHLKKLEGAILWIPAFFLHTSATVTRT
LVDFFVEHKGQLKVELAWPGNIMHNVNKYWKTKNLSPKRLSTGILMYTLAFSMCDEIHLYGF
WPFGWDPNTGKDLPYHYYDKKGTKFTTKWQETHQLPSEFKLLYKLHREGVTKLGLSHCT

>Gasacu_GT29_ST8Sia3

MVRIVSALGLVMFSVALLILSLISYVSIKKDFLLSTPRYGANGGPRMSMLHAGFRSQLALKY
LDPAFSPLTNALGEDLQNSSKWTYNSSAFLQLKNEISQYIDIPHNFTLTRDSVRIGQLMHYD
YSGHKYVFSIGENFRSLLPEVSPILNKHYNVCAVVGNSGILTGSRCGPQIEKFDFVFRCNFA
PTEIFKKDVGRRTNMTTFNPSILEKYYNNLLTVQDRNNFFLSLKKLDGVILWIPAFFFHTSA
TVTRTLVDFFVEHRGQLKVRLAWPGNIMQYVNSYWKTKQLSPKRLSTGILMYTLASTMCDQI
HLYGFWPFGWDPNTGKELPYHYYDKKGTKFTTKWQESHQLPAEFKLLYKMHTQGLLKLSLSH
CA

>Gasacu_GT29_ST8Sia3-r

MVRIAKALGLVILCVAVLILSLISYVSLRKDSLFGSSKYYMGGPRIMFHAGFRSQFAMNFLD
PSFIPLTNALNEELQGKPSKWKFNKTAFHLQKKDIFSYIDIPTNFSLTKNSVRVGQLMHFDY
SSHKYVFSISNNLKSLLPDASPIRNKHYSVCAVVGNSGILTGSHCGPEIDQADFVFRCNFAP
TEVYSKDVGKKTNLTTFNPSILERYYNNLLTIQDRNNFFLNLKKLEGAILWIPAFFLHTSAT
VTRTLVDFFVEHKGQLKVELAWPGNIMHDVNKYWKTKNLSPKRLSTGILMYTLASAMCDEIH
LYGFWPFGWDPNTGNDLPYHYYDKKGTKFTTKWQETHQLPSEFKLLYKLHRDGVIKLSLTHC
S

>Homsap_GT29_ST8Sia5

MRYADPSPNRDLLGSRTLLFIFICAFALVTLLQQILYGRNYIKRYFEFYEGPFEYNSTRCLE
LRHEILEVKVLSMVKQSELFDRWKSLQMCKWAMNISEANQFKSTLSRCCNAPAFLFTTQKNT
PLGTKLKYEVDTSGIYHINQEIFRMFPKDMPYYRSQFKKCAVVGNGGILKNSRCGREINSAD
FVFRCNLPPISEKYTMDVGVKTDVVTVNPSIITERFHKLEKWRRPFYRVLQVYENASVLLPA
FYNTRNTDVSIRVKYVLDDFESPQAVYYFHPQYLVNVSRYWLSLGVRAKRISTGLILVTAAL
ELCEEVHLFGFWAFPMNPSGLYITHHYYDNVKPRPGGHAMPSEIFNFLHLHSRGILRVHTGT
CSCC

>Pantro_GT29_ST8Sia5

MRYADPSANRDLLGNRTLLFIFICAFALVTLLQQILYGRNYIKRYFEFYEGPFEYNSTRCLE
LRHEILEVKVLSMVKQSELFDRWKSLQMCKWAMNISEANQFKSTLSRCCNAPAFLFTTQKNT
PLGTKLKYEVDTSGIYHINQEIFRMFPKDMPYYRSQFKKCAVVGNGGILKNSRCGREINSAD
FVFRCNLPPISEKYTMDVGVKTDVVTVNPSIITERFHKLEKWRRPFYRVLQVYENASVLLPA
FYNTRNTDVSIRVKYVLDDFESPQAVYYFHPQYLVNVSRYWLSLGVRAKRISTGLILVTAAL
ELCEEVHLFGFWAFPMNPSGLYITHHYYDNVKPRPGFHAMPSEIFNFLHLHSRGILRVHTGT
CSCC

>Musmus_GT29_ST8Sia5

MRYADPSANRDLLGNRTLLFIFICAFALVTLLQQILYSKSYIKRYFEFYKEPLEFNSTRCLE
LRQEILEVKVLSMVKQSELFERWKSLQICKWAMGASEASLFKSTLSRCCNAPNFLFTTQKNT
PVETNLRYEVESSGLYHIDQEIFKMFPKEMPYYRSQFKKCAVVGNGGILKNSGCGKEINSAD
FVFRCNLPPISGIYTTDVGEKTDVVTVNPSIIIDRFHKLEKWRRPFFSVLQRYENASVLLPA
FYNVRNTLVSFRVKYMLDDFQSRQPVYFFHPQYLSSVSRYWLSLGVRARRISTGLSLVTAAL
ELCEEVHLFGFWAFPMNPSGFFITHHYYDNVKPKPGFHAMPSEIFTFLRMHSRGILRVHTGT
CNCC

>Macmul_GT29_ST8Sia5

MRYADPSANRDLLGNRTLLFIFICAFALVTLLQQILYGRNYIKRYFEFYEGPFEYNSTRCLE
LRHEILEVKVLSMVKQSELFDRWKSLQMCKWAMNISEANQFKSTLSRCCNAPAFLFTTQKNT
PLGTKLKYEVDTSGIYHINQEIFRMFPKDMPYYRSQFKKCAVVGNGGILKNSRCGREINSAD
FVFRCNLPPISEKYTMDVGVKTDVVTVNPSIITERFHKLEKWRRPFYRVLQVYENASVLLPA
FYNTRNTDVSIRVKYVLDDFESPQAVYYFHPQYLVNVSRYWLSLGVRAKRISTGLILATAAL
ELCEEVHLFGFWAFPMNPSGLYITHHYYDNVKPRPGFHAMPSEIFNFLHLHSRGILRVHTGT
CSCC

>Ratnor_GT29_ST8Sia5

MRYADPSANRDLLGNRTLLFIFICAFALVTLLQQILYSKSYIKRYFEFYKEPLEFNSTRCLE
LRQEILEVKVLSMVKQSELFERWKSLQICKWAMDASEASLFKSTLSRCCNAPNFLFTTQKNT
PVETNLRYEVESSGLYHIDQEIFKMFPKEMPYYRSQFKKCAVVGNGGILKNSGCGKEINSAD
FVFRCNLPPISGIYTTDVGEKTDVVTVNPSIIIDRFHKLEKWRRPFFSVLQRYENASVLLPA
FYNVRNTLVSFRVKYMLDDFQSGEPVYFFHPHYLSSVSRYWLSLGVRARRISTGLILVTAAL
ELCEEVHLFGFWAFPMNPSGFFITHHYYDNVKPKPGFHAMPSEIFTFLRMHSRGILRVHTGT
CNCC

>Bostau_GT29_ST8Sia5

MHYADPSANRDLLGNRTLLFIFICAFALVTLLQQILYGRNYIKRYFEFYEGPFEYNSTRCLE
LRHEILEVKVLSMVKQSELFDRWKSLQMCKWAMNISEANQFKSTLYRCCNAPPFLFTTQKNT
PLGTKLKYEVDTSGIYHINQEIFRMFPKDMPYYRSQFKKCAVVGNGGILKNSRCGREINSAD
FVFRCNLPPISEKYTMDVGVKTDVVTVNPSIITERFHKLEKWRRPFYRVLQLYENASVLLPA
FYNTRNTDVSIRVKYVLDDFESPQAVYYFHPQYLVNVSRYWLSLGVRAKRISTGLILATAAL
ELCEEVHLFGFWAFPMNPSGLYITHHYYDNVKPRPGFHAMPSEIFNFLHLHSRGILRVHTGT
CSCC

>Galgal_GT29_ST8Sia5

MGYTDPSSGRDLLGNRTLFFIFICAFAVVTLLQQILYGRNYLKRYFEFYEGPFEYNSTRCLE
LRHEILEVKVLSMVKQTELFDRWKSLQMCKWEMNVTEANILKSTLSRCCNAPAFLFTTQKNT
PLGTKLKYEVDTSGIFHINQEIFKMFPKDMPYHRSQFKKCAVVGNGGILKNSRCGREIDSAD
FVFRCNLPPISEKYLTDVGVKTDVVTVNPSIITERFHKLEKWRKPFYDVLQVYENASVLLPA
FYNTRNTDVSIRVKYVLDDFESQQAVYYFHPQYLINVSRYWLGQGVRAKRISTGLILVTAAL
ELCEEVHLFGFWAFPMNPSGIFITHHYYDNVKPRPGFHAMPSEIFNFLHMHSKGILRVHTGT
CGCC

>Siltro_GT29_ST8Sia5

MGYADPSGGRDLLGNRTLLFIFICAFALVTLLQQILYGRNYIKRYFEFYEGPLDFNSTKCLE
LRRDITNVKVLSMVKESDLFDRWKSLQVCKWEMNVTEANAFKSALTRCCNAPSFLFTTQKNT
PLGTKLRYEVDTSGIFPISSEIFNIFPKQDMPYYRSQFKKCAVVGNGGILKDSKCGKDIDST
DFVFRCNLPPITPKYVEDVGMKTDVVTINPSIITERFNKLEKWRRPFYEVLQGYENASLLLP
AFYNTRNTDVSIRVKYVLDDFESQQAVYYFHPQYLINVSRFWLMQGVHAKRISSGLILVTAA

LELCEEVHLYGFWGFPMDPSGNFITHHYYDNVKPRPGFHAMPSEIFNFIHMHSKGILRVHTG
TC

>Takrub_GT29_ST8Sia5

MLRRKKAYADTSSGKDILGNRSLCFIFICAFGLVTLIQQILSGKNYIKRYLGNYDGPFEYNS
TTCRELRQEIMDVKVLTMVKTSDLFERWRNLQICRWEQNKEETSNFKMSLSRCCNAPSFLFT
TKRNTPAGTKLRYEVDTSGILPITAEVFKMFPDDMPYSKSQFKKCAVVGNGGIIKNSKCGKE
IDSADFVFRCNIPPISEKYSADVGTKTDLVSINPSIITERFQKLEKWRRPFYEVLQNYENSS
AVLPAFYNTRNTDVSFRVKYMLDDFDSQRGVFFFHPQYLLNVQRFWAVQGVRAKRLSSGLML
VTAALEMCEEVHLYGFWAFPMNPSGIFITHHYYDNVKPRPGFHAMPHEIFNFIHMHTRGIVN
VHTGQCT

>Tetnig_GT29_ST8Sia5

MLRRKKTYPDTSSGKDILGNRSLCFMFICAFGLVTLIQQILYGKNYIKRYLGSYDGSLEFNS
TTCRELKQEIMDVKVLTMVKTSDLFERWRNLQVCKWEQNKEETSNFKMSLSRCCNAPSFLFT
TRRNTPAGTKLRYEVDTSGILPITAEIFKMFPDDMPYAKSQFKKCAVVGNGGIIKNSKCGTE
IDSADFVFRCNIPPISEKYSADVGTKTDLVSINPSIITERFQKLEKWRRPFYEVLQNYENSS
VVLPAFYNTRNTDVSFRVKYMLDDFDSQRGVFFFHPQYLLNVQRFWAVQGVRAKRLSSGLML
VTAALEMCEEVHLYGFWAFPMNPSGIFITHHYYDNVKPRPGFHAMPHEIFNFIHMHTRGIVN
VHTGRCV

>Gasacu_GT29_ST8Sia5

MLRRRMGYADPSSGKDILGNRSLCFMFLCAFGLVTLLQQILYGKNYIKRYLENYDGSYEYNS
TACRELRQEIMDVKVLTMVKTSDLFERWRNLQVCRWEQNKEETSNFKMSLTRCCNAPSFLFT
TKKNTPAGTKLRYEVDTSGILPITTEVFKMFPDDMPYSKSQYKKCAVVGNGGIIKSTKCGKE
IDSADFVFRCNLPPLDNGYENDVGIKTDLVTANPSILVCRFQKLEKWRRPFYEVLQKYENSS
VVLPAFYNTRNTDVSFRVKYMLDDFDSQRGVFFFHPQYLLNVQRFWAVQGVRAKRVSSGLML
VTAALEMCEEVHLYGFWAFPMNPSGIYITHHYYDNVKPRPGFHAMPHEIFNFIHMHTRGIVN
VHTGQCT

>Danrer_GT29_ST8Sia5

MGYSDPTASRDLLGNRSLCFIFICAFGLVTLLQQILYGKNYIKRYLERIDGSLQFNSSSCKE
LRQDITDVKVLTMVKTSELFERWRNLQVCKWDQNKEETDNFKMSLSRCCNAPSFLFTTKRNT
PSGTKLRYEVDTSGILHISPEIFKMFPDDMPFSKSQFKKCAVIGNGGIIKNSKCGREIDASD
FVFRCNIPPVSDLYSQDVGSKTDLVTINPSIITERFQKLEKWRKPFYEVLQNYENSSVVLPA
FYNTRNTDVSFRVKYMLDDFESSRGVFFFHPQYLLNVQRFWAVQGVRAKRLSSGLMLVTAAM
ELCEEVHLYGFWAFPMNPSGIFITHHYYDNVKPRPGFHAMPYEIFNFMHMHARGIVHVHTGP
CR

>Anocar_GT29_ST8Sia5

MGYTDPTSSRDLLGNRALLFIFVCAFAVVTLVQQILYGRNYLRRYFDFYEGPLEFNSSKCLE
LRKGITDVKVLSMVKQTELFERWKNLQMCKWEMNITQANIFKATLLRCCNAPAFLFTTQKNT
PLGTKLKYEVDTSGIFHVNQETFRMFPQEMPYSRSQFKKCAVVGNGGILKNSRCGREIDSAD

FVFRCNLPPISEKYIADVGVKTDIVTVNPSIITERFHKLEKWRKPFFNVLQTYENASVLLPA
FYNTRNTDVSIRVRYALDDFESQQSVYFFHPQYLINLSRYWLNQGVRAKRISTGLILVTAAL
ELCEEVHLFGFWAFPMNPSGIYITHHYYDNVKPRPGFHAMPSEIFNFLHMHSKGILRVHTDA
CNCC

>Calmil_GT29_ST8Sia5

MGYSERSGKDILGNRSLFFIFISAFAIVTLLQHILNGKNYIKRYFELTEGSYDYNSSTCREL
RNDITEVKVLSMVKQSELFERWRTLQLCKWELNKTEANTARLTNCCNAIQNFTVTQVNTALG
ANLTYDAQPKKQITITEDIFNLLPQEMPYSRSQFKKCAVVGNGGILKKSECGKEINSADFVF
SCNLPPLSNEYARDVGHRTQLVTANPSIIKKRFQKLDKWRRPFVELLQVYENTSVVMPAFYN
TRNTDVSLRVRYALDDFSAPQDLFYFHPRYLENVARFWGRQGVRAKRPSSGLMLVTAAMELC
QEVHLYGFWGFPMDPSGIHITHHYYDNVKPRPGFHSMPNEIFTFLHMHSRGVLQVHTTPCR

>Orylat_GT29_ST8Sia5

MRRRMGYSDPSSGKDILGSRSLCFMFICAFGLVTLLQQILYGKNYIKRYLETYDRSLEFNST
TCRELRQEIMDVKVLTMVKTSDLFERWRNLQVCKWEQSKEETSNFKLSLSRCCNAPSFLFTT
KRNTPAGSKLRYEVDTSGILPITNEVFKMFPHDMPYSKSQFKKCAVIGNGGIIKNSKCGKEI
DSADFVFRCNIPPIKEKYSTDVGSKTDLVTINPSIITERFQKLEKWRRPFYDVLQNYENSSV
VLPAFYNTRNTDVSFRVKYMLDDFDSQRSVFFFHPQYLLNVQRFWAVQGVRAKRLSSGLMLV
TAALEICEEVHLYGFWAFPMNPSGVYITHHYYDNVKPRPGFHAMPHEIFNFIHMHTRGIINV
HTDQCM

>Danrer_GT29_ST8Sia7-A

MALTSLRWGSTFLVVLIIFQCVCVMFYKQNTPSFGDVMTDTFQKMGCAKLRHKFSVIKPAKS
IKFKSFAREVSDFLSCPYKSNETEQELNRIRLRFCCNATESFVLTKRNTAISQSIPYETNSR
RTYKMNADVHKLLPEDSPWSAGALGLCAVVGSSGILKNSSCGRQIDSADYVIRFNLALINDS
DVGLKTNLITINPSQIRGYRNLEKKPGPLVKRVSVYGNSSLIIPAFAYVICTSPSLRVLRVL
QPIRPQQPVLFFSPHYLRALDRFWKGRGLKERRLSTGFMLISAALEMCEHVHVYGFWPFGTD
LQENPIPYHYYDQMRSGSVHRMPEEFLRLLQLHSQGALTLHLQPCDTH

>Danrer_GT29_ST8Sia7-B

MALVQWVFRLLVVLIVSQSVCVMFFYTLDTHSHGCRMRLQYCCNATHSLILTKRNTAVDHII
TYETEGKRTYRVKADLHKMLPEDSPWSAGALGLCAVVGSSGILKNSSCGRQIDSADFVIRFN
LALVNDSDVGLKTDLMNINPSQIRRYKTMEQDPGPLVERISVYGNSSLIIPAFAYGFCTSPS
LRVLRVLQPIRPQQPVVFFNPHYLRALDRFWKWRGLKEPRLSSGFILISAALEMCEHVHVYG
FWPFGTDLQENPVPYHYYDQSKPSPYMHRMPEEFVRLLQLHSQGALTLHLQPCHTHRAVFPN
PVPLGTPTVLIIGCLPYLRHPGFSKS

>Anocar_GT29_ST8Sia7-A

TQLQQCCNASFRFVTTKENIRLGSYIIFDGNPTRKLRVDTKLLDLLPEKSPFMDTSYRKCAV
VGNGGILLNSSCGQEIDRADLVIRFNLPPMNFSEDIGTKTSLVTINPSILQNRFKLLQERRK
PFVEALHSYSDATFLLPVLSFVGHNILGYRVLYTLEDFGVEQQAFFLNPQYLSNLANFWKKR

GLKTNRLSSGFMLVSMALEFCQHITLYGFWPFSYDLNNQSIPHHYYDNMMPTPGVHAMPTEF
SYYLSMYAEGVLRLRVGKCQ

>Oncmyk_GT29_ST8Sia7

MCYLVLFILLCSGTLLSITLIDYYQHQRDGFLDSVQKGLQCRRLRKRLVTMSTIKKVDIALF
SQDVRELMDCPWRLNLTHRELYRTELWSCCNASDRLMVTRQNTNLNQSLSYEIHRWKKRKVD
QALWEMLPQTVPWSKGSLSRCAVVGSGGILQNSSCGAEIDNADYVIRFNLAPINKSCDVGVK
TDLVTANPSQIIKGYTDLQQNPGHLAEVLSFYGHAHLLLPAFSSASGTAPCFKVYHALRKAR
PQQEVVFFHPDYLFELGRFWRHRGQRARRLSTGLMLASTALEICEQVHLYGFWPFPLDLSHN
SLPHHYYDNVGPNRKMHAMPEEFLLLLQLHSQGALQLHVGPCTLTPTP

>Esoluc_GT29_ST8Sia7

MRCWSAATRFCLLFVFLLFVLSSTIMNDYQFPRDGFMESVQKSLRCRKLRERLVAMTTMKKV
DIVPFSQVVRELMDCPWRLNLTHRELHRTELWACCNASDRLIVTRQNTHVNQTLTYEAERWR
KKLVDQTLWEMFPKNVPWSKGLLGRCAVVGSGGILQNSSCGEEIDRAHYIIRFNLAPVNQSR
DVGLKTDLITANPSQIIKQYPDLHKNPGRLAEQMSVYGNARLLLPAFSFAFGTAPCFKVYHA
LQKALPQQEVVFFHPDYLFELGQFWRRRGQKASRISTGLMLASSALEICDQVHLYGFWPFSV
DLSHNPLQHHYFDNVGPNHYMHAMPEEFLLLLQLHSQGALQLHVGHCR

>Colliv_GT29_ST8Sia7

MFMAFSKMPNNIIQKEYVRQGLLKMHKLWLTLLPVAAVSLVYMYFLVLNDRPDIEVPEAEAC
QRLLSNLTQPQKAELGRCCNASAWLAVTQDNTPLGSEIVYDAYPSKRLKVSSGLLEILPEKS
PFQESPYKTCAVVGNGGILRNSSCGSKIDGHEFVIRFNLPSVDFPEDVGKKSSIVTVNPSIL
HKRFHGLNGRRLPFVKAAASYGKTWFLIPAFSYPSNSEASYRALYALQDSASQSHIFFFHPQ
YLSSLSKYWHDHGFHTPRLSSGFMLVNAALELCQHITLYGFWPFSLHPDGHSLPHHYYDNVL
PNSRIHIMPKEFTYYVDMHFHGVLQLHLGR

>Latcha_GT29_ST8Sia7

MSEENRKRCGELRENLTSQELPKETDLNKCSNLTQRLVLTRENAPIGHEIEYEVAEQRVRVE
EPLFKLLPRFPPPFYQESPFKKAPYKRCAVVGNAGILLNSGCGRRIDQADFVFRCNLPPLNY
SKDVGSKTDLVTANPSIIIQKYHGLKESRRLFVERMKVYKDALILMAAFSQSFATEISLAVA
YALEDFGSKQKAIFFHPAYLRQLGSLWRSWGVRARRLSSGLMLVSAALELCDSVALYGFWPF
STGLDGEAVLHHYYDNVPPEPGVHAMSSEFVHYLQMHSKGVLQLHVGKC

>Chrpicbel_GT29_ST8Sia7

MLRRQSWALVLAVGITLSFSLSVLWRLQSPEATGMDAEQCRELGQELSQRVLPVRLHEAWIW
RRLKLMQSCPWAYNARALGRYREQLGHCCNASADLVLTRDNTPLGSRIVCDGQPAKKLLVQE
ALLEILPQGAPYDSCAVVGNGGILHNSGCGPEIDRAQFVIRFNLPPMGFAEDVGTKSSVITV
NPSILVLRFGALSRWRRPFAEAMGTYGAPLLLIPAFSFISFAAVSSQALYTLEDFGSPARAV
FMNPEYLAGLDGHWHRRGLRAKRLSSGFMLVNAALELCQHLTLYGFWPFPTDPEGRPLPYHY
YDKQTPKPGVHAMPDEFTRYLGMHLQGALRLHLGRCQEGLAGGHAQQGSDGG

>Lepocu_GT29_ST8Sia7

MALRCLQLQREVLGMQSLKKVNSTLLCGQVTQLMRCPWTLNLTQLEQQRTELRLSCNATGRL
VVTQDNTPLGGTITYDAEKWTRQVDTEIFNMLPQSPPWAGGPGARLRRCAVVGNGGILRNSS
CGAHIDRADFVIRFNLPPLNYSRDVGVKSSLVTVNPSQIINSYRNLNYARRPFVQRVSTYEN
AHLVLPAFAFSFCTDPCFRVYYTLQDNRPHQRALFYHPDYLRQLATYWQEKGLRETRLSTGL
MMVSAALELCDRVELYGFWPFSFDLSERPLSNHYYDDVPPNRGMHAMPEEFLRLLRMHSQGV
LRLRVGQCP

>Deiacu_GT29_ST8Sia7

MKPLRRPHPAPDLVFDMSALDSSLQVIILLPAPLNGVLLKKSSKWTPTSANLFCSRALDCCV
CGFHLGFSLSCFSFEDSKTWCVLRCATGERTAGIHPPFDGVKVKIITVDSKLTNMLLERFPL
ADAQYSKCALIGNGGILQDSRCGQEIDQADFIIRFNLPPLNRTEDVGTKTHLVTINPSILTN
RFKSLVRSPVAFIDAVRAYPNALFLIPALSFHDHIELGYRALHILKDIGLPHQAFFLNPHYL
GALDTYWKQKGMTEIRLSTGFMFTSFALEFCDHITLYGFWPFLFDLTGKPINHHYYDNVLPH
PFVHSMSEEFSRYIDMYAQGVLRIQLGKC

>Pytmol_GT29_ST8Sia7

MKPLRRPHPAPDLVFDMSALDSSLQVIILLPAPLNGVLLKKSSKWTPTSANLFCSRALDCCV
CGFHLGFSLSCFSFEDSKTWCVLRCATGERTAGIHPPFDGVKVKIITVDSKLTNMLLERSPL
AGFQYNKCALIGNGGILRHSSCGQEIDQADLIIRFNLPPMNYTEDVGTKTSLVTINPSILNN
KFQSLQGPQKPFLDALQAYRDALFLIPSLSFSSHHVLGCRAVSIMKDSGLTHRAFFLHPHYL
GAIRKYWEQKGLKEIRLSTGFLFISIALEFCEHITLYGFWPFSYDLTGEPLSHHYYDNILPN
AGFHTMSEEFLHYLNMYAQGVLRIQLGKC

>Strcamaus_GT29_ST8Sia7

MPNNISTEEYVNPGLLKVRKLWTGLLTMAAVSVVSMFFLVWSDGRPDIRVPEPEACQVLSSS
LTESFLKRVDEVWSLSHLKLMQSCRWNFNASALAQYRAELGHCCNASAWLALTQVNTPLGSN
IVYDGYRSKSLKVSSGLLEILPEESPFQEPFYKTCAVVGNGGILRNSSCGSEIDEHQFVIRF
NLPSMDFPEDVGRKSSIVTVNPSILQKRFHGLNGRRLPFVKAAAIYGETWFLIPAFSYPGQN
EASYRALYALQDSGSRSPIFFFHPQYLSALTRYWHEHGFHPTRLSSGFMLVNAALELCQHIT
LYGFWPFSLHPDGHPLPHHYYDNQLPNPRMHLMPQEFACYVNMHFQGVLQLHVGKC

>Apavit_GT29_ST8Sia7

MPHSISREEYVKPGLLKMHKLWVMLLAVAAVSFVSMCFLVLNGRPDIKVSEAEACQELLSNI
TQPHKAELGHCCNASAWLAVTQENAPLGSEIVYDGYPSKRLKVSSGLLEILPEKSPFQNPLY
KTCAVVGNGGILRNSSCGAKIDEHQFVIRFNLPSMDFPEDVGRKSSIVTVNPSILQKRFHGL
NGRRLPFVKAAAPYGKTWFLIPSFSFPDDTEASYRALYALQDSASESHVFFFHPHYLSALSK
YWHDLGFHTYRLSSGFMLVNAALELCQHITLYGFWPFSFHPDGHSLPHHYYDNVLPKQNIHI
MPKEFSYYVDMHFHGVLQLHLGRC

>Gekjap_GT29_ST8Sia7

MYHHLRLAKRDFMLVVSVVFLVSLTLSLMQRTTRSVLLHMTAREEELVRAVEKLPLQKLDEE
ILAHLLLTQGCPWQASARAMAQYRTELGRCCNASFWLAITKENTPLGSDILLDGNKGKKLPV
GAELMDLLPERSPIPGILYDQCAVVGNGGILQNSSCGQEIDQADLIIRFNLPPMNYSEDVGT

KTSLVTINPSILQTKFNKLEAHRKPFADALRPYRSALVFIPAFSFVGHSELAYRALYTMEDF
GTGQRAYFWNPHYLDTLGIYWKARGFYPHRLSSGFMLVNMALEFCKRITLYGFWPFSHDPAG
RPIPHHYYDNTWPKPGVHAMSQEFSHYLHMYAQGVLQLRLGSCQ

>Promuc_GT29_ST8Sia7

MDEVLRWQLMLIQECPWKPNATAVMQHRAELGQCCNASHRLVVTKENAPLGSTIHFDGVKVK
IITVDSKLTNMLLERFPLADAQYSKCALIGNGGILQDSRCGQEIDQADFIIRFNLPPLNRTE
DVGTKTHLVTINPSVLTNRFKNLVGPPMAFIDAVRAYPNALFLIPALSFHDHIELGYRALHI
LKDIGLPHQAFFLNPHYLGALDMYWKQKGMTEIRLSTGFMFTSFALEFCDHITLYGFWPFLF
DLTGKPISHHYYDNVLPNAFVHSMSEEFSRYIDMYAQGVLRIQLGKCQ

>Ambmex_GT29_ST8Sia7

MRLKGRPVNHLALMVVSCTTFILLYTFSVPADREQEELVCKRSPEVTITHSKAKCQKLRRIF
ATLSAHKKIGEKSIMRNAAELQRCPWQENREKKEFYRSEINQCCNSSHGLIVTQENTRVGEN
IVYETQKSVKINVTTEIFNMFPKESPFSGKAYKTCAVVGNGAILVDSCCGQQIDQAEFVFRF
NLPPLNYTKDSGKKVDLVTANPSIMINRFQSLNHRRKAFVDMLRAYRGALILMPTFSHTFGT
QLAFKVQYTLDDFGLGSRMLSFHPVYLENLAKYWRSKGLYVKRLSSGIMLVSAAMELCDSIT
LYGFWPFYSNLEGRTIGYHYYDKMAPNIQVHAMPKEFLIYSQMHAQGALKLQVGQCS

>Rancat_GT29_ST8Sia7

HHRVWKHGANVSTSECSHLRSLILNSVLRIFKQQAFFKVVKTLQGCPWKEDQTERNLLQANL
GKCCNASYSMIMTQENTVIGQTITFDAETKIKQNITKSLYSLFPKKSPFQKPIRTCAVVGNG
GILTNSSCGAQIDGADFVFRLNFPPLNWTDDIGAKTDLVTSNPSILMNKFSSLTEKRKPFIM
MVQEYSSPLILLPAFSYSANTEVSLRVLYTIEDFELNSKVVFFNPEYLKNLSAYWKSMGLKF
GRLSSGLMVVSIAMEVCDKVTLFGFWPFSKDLNGVPILHHYYDNVPPKPGIHAMPDEFYKYL
QMHIQGSLRLNLEHC

>Malterter_GT29_ST8Sia7

MVAPPRMLRRQSWALVLAVGIALSFSLSVLWRLQSQASQPKTALAAGKGARLDPKHPCRTMD
QNRSPIPEALVMGAEQCRGLMQNWSAGTLPAREQLGSSCNASANLVLTQNNTQLGSQIVYDA
QRDKKHPVKEELLEMLPQGSPFQGAPYECCAVVGNGGILRNSSCGSEIDRAQFVIRFNLPPM
DFADDVGTKSSVVTMNPSILHARFRGLSRWRRPFAEAVGIYGAPLLLIPAFSFVGQSTVSFQ
ALYTLEDFGSPARAVFMNPEYLARLDGHWRPRGLRAKRLSSGFMLVSAALELCQHLTLYGFW
PFPTDPEGRLLPHHYYDNQPPKPGIHAMPDEFTRYLGMHMQGALRLHLGRCQ

>Sinrhi_GT29_ST8Sia7

MALAFLRWIFRILMVLIVFQGVFVMFYYTPGTHSSRDGMTDTLQLLRCAKLRHKFSIIKPAK
SLNLESFTQELSDFMSCPYKSNTTERELNRMKLQFCCNATRSLFLTKRNTAVNQTIPYETST
ISTYTMNDTLHSMLPEDFPWSGRRLGRCAVVGSGGILKNSSCGREIDSADFVIRFNLASIND
SDVGLKTDLVTINPSQIQREYKDLEKKPNPLVKRVSVYGNASLIMPAFAYTFCTGLSIRTLK
ALHPIRPQQPVVFFSPYYLQTLDRFWKGRGLKSIRLSTGFMLISAALEVCEHVHVYGFWPFD
TDLQDNPVPYHYYDLRRPSKRMHKMPEEFVRLLQLHSQGALTLHLQPCSSDAS

>Sinans_GT29_ST8Sia7

MALAFLRWIFRLLTLLVVSHSVCVIFYYTADTHSSREAMPDTLQRLRCAKLGRKFSVITSVK
RLNMESFTEELVDFLKCPQKPNTTEQELNRMKLQFCCNATGSIFLTKRNTAINQTIPYETDT
KHTYRMNAAIHNMLPEDFPWSGRRLGRCAVVGSGGILKNSSCGREIDSADFVIRFNLASIND
SDVGLKTDLVTINPSQIQREYKDLEKKLNPLVKRVSVYGNTSLIMPAFAYTFCTGLSIKTLK
ALHPIRPQQPVVFFSPYYLQTLDHFWKGRGLKSIRLSTGFMLISTALELCENVHVYGFWPFD
TDLQDNLVPYHYYDLRRPSKSMHKMPEEFVRLLQLHSQGALTLHLQPCSSDAS

>Gymprzprz_GT29_ST8Sia7

MALVFLRWIFQLLTVLIVFQGVCVMFYYTRSDTHSSRDGMTETLQLLRCAKLRHKFSIIKPA
QSIKLESFTQELSDFMNCPHKPNTTERELNRMRMQFCCNATGSLFLTKRNTAINQTIPYETS
TIRTYNMNPVIHSMLPEDFPWTGRRLGRCAVVGSGGILKNSSCGREIDSADFVIRFNLAYIN
DSDVGLKTDLVTINPSQIRYKNLEQNPDPLVERVSVYGNASLAMAAFAYTFCTGLSIKTLKV
LHPIRPQQPVVFFSPIYLRTLDRFWKGRGLKSIRLSSGFMLINTALELCEHVHVYGFWPFGT
DLQNNSVPYHYYDELSPHRYKHKMP

>Catcat_GT29_ST8Sia7

MALAFLRLVFRLLMVLMVFQGVCVMIYYTRNTHSPRDGTADRLQKLRCTKLRHKFAIIKPAK
SIQLQRFTQELSDFMSCPYKSNTTERELNRMRLQYFCNATGSLFLTKRNTAINQTIPYETST
VKTYKMNAAIHGMLPEDFPWSGRRMGRCAVVGSGGILKNSSCGREIDSADYVIRFNLASIND
SDVGLKTDLITINPSQIQFKNLDKNPEPLVERVSVYGNASLIMPAFAYTFCTGQSIKTLRVL
HPIRPQQPVVFFSPTYLRTLDRFWKGRGLKEVRLSTGFMLISTALELCEDVHVYGFWPFGTD
LHNNSVPYHYYDQLSPHRYMHAMPEEFVRLLQLHSKGALTLHLQPCS

>Pimpro_GT29_ST8Sia7

MASASLQWIFTLMMVLVIFQGVCVMFYYTADTHSSRDAITKGFLRLHCEKLRRKFSIIKSAK
SINFKRFTQELSDYLSCPYKSNQTERELNRIRLQFCCNATGSFILTKRNTAITQSIQYETST
KTYKMKAELHKMLPEDIPWSGRRLGRCAVVGSGGILKNSSCGREIDNADFVIRFNLAEVNDS
DVGLKTDLITINPSQIRYKNLQNNPDPLVERVSVYGNASLIIPAFAYTFCTSVSIKVLKVLQ
PIRPHQPVVFFSPSYLRSLDRFWKGRGLQEERLSTGFMLISTALELCEHVHVYGFWPFDNDL
QDQPIPYHYYDLSRPSSHRHKMPEEFVRLLQLHSQGALTLHLLPCDSPETDRENNL

>Salsal_GT29_ST8Sia7

MRYSIYLVLFILVCSASLLSVTLISRYYHKRSGGFDSYAAFRCNRLRLKFLTLTSAKAINMR
TLIMDVRQLMTCTHRPNITQRELYRVILRSCCNATGEMILTKQNTKLGQKIHYETNQKLFKT
VDKKLHSMLPKDLPWSKGLLSHCAVVGSGGILQNSSCGAEIDSADYIIRFNLGPVTNSKDVG
NKTHLMTINPSQIRGYRNLTKAPQPLANRVAVYGNASLLLPAFSYQFSTGLSLDVYHALQPL
RPYQKVVFFNPNFMLNLGRKWKGQGLKEPRLSTGLMLASVAMELCEEVHIYGFWPFSLDLNH
NPLPHHYYDNVGPKIGFHSMPEEFQLLLKLHTQGALQLHLGRC

>Salsal_GT29_ST8Sia7-B

RDEFLDSVQKSLQCRRLREKLVTMTSMKKVDIALFSQEVRELMNCPWRLNITHRELHRTELW
SCCNASDRLMVTRQNTNQNQSLTYEVERRMKRKVDQALWEMLPQTVPWSKGSLSRCAVVGSG
GILQNSSCGAEIDSADYIIRFNLGPVTNSKDVGNKTHLMTINPSQIRGYRNLTKAPQPLANR
VAVYGNASLLLPAFSYQFSTGLSLDVYHALQPLRPYQKVVFFNPNFMLNLGRKWKGQGLKEP
RLSTGLMLASVAMELCEEVHIYGFWPFSLDLNHNPLPHHYYDNVGPKIGFHSMPEEFQLLLK
LHTQGALQLHLGRC

>Salfon_GT29_ST8Sia7

MRYNIYLVLFILVCSASLLSLTLIRRYSHKSINTRTLIMDVRELMNCPHRPNITQRELYRVI
LRSCCNATGEMILTNQNTKSGQKIHYETNQKLFKTVDKKLHSMLPKALPWSKGLLGHCAVVG
SGGILQNSSCGAEIDSADYIIRFNLGPVTNSKDVGNKTHLMTINPSQIRGYPNLTKDPQPLA
NRVAVYGNASLLLPAFSYQFCTGLSLNAYHALQPLRPNQKVVFFNPNFMLNLGRKWKGQGLK
EPRLSTGLMLASVAMELCEEVHIYGFWPFSLDLNHNPLPHHYYDNVGPKKGVHSMPDEFQLL
LKLHTQGVLQLHLGRC

>Corclu_GT29_ST8Sia7

MRYSIYLVSFILMCSTSLLSVTLIGRYYHKRSAGFDSYAAFLCNRLRLKFLTVTSAKAINPR
TLIMDIRQLMDCSHRPNITQRELYRVILRSCCNATGEMILTNQNTKLGQKIHYETNQKLFKT
VDKKLHSMLPNALPWSKGLLGRCAVVGSGGILQNSSCGAEIDSADYIIRFNLGPVSNSEDVG
NKTHLMTINPSQIRTGYRNLTKDPQPLANRVAAYGNASLLLSPFSYQFCTGLSLDVYHVLRP
LRPNQKVVFFNPNFLLQLSRKWKGRGLKEPRLTSGLMLASVAMELCEEVHLYGFWPFPLDLY
HNPLPHHYYDNVGPKKGVHSMPDEFLLMLQLHTQGVLQLHLGGC

>Thythy_GT29_ST8Sia7

MRYLILFILVCSGTLLSIILIDYYQHQRDGFLDSVQKRLHCRRLTEKLVTMTTMKKANIALF
SQEVRELMDCPWRLNLTHRELHRTELWSCCNASDRLMVTRQNTNQNQILTYEAEKWRKRKVD
KALWEMLPQTVPWSKGSLSRCAVVGSGGILQNSSCGAEIDTADYVIRFNLAPINKSCDVGVK
TDLITANPSQIIKGYHNLQRNPGPLVKRMSVYGHAHLLLHAFAFGFGTSPCFKVYHALRKAR
PQQEVVFFHPDYLLQLDRFWRRRGQRAPRLSTGLMLASTALEICEQVHLYGFWPFHLDLSQN
TLPHHYYDNVGPSRFMHAMPEEFLLLLQLHSQGALQLHVGPCTL

>Thythy_GT29_ST8Sia7-B

MRYSIYLVTFIMVCSASLLSVTFIGRYYHKRSGGFDGYAAFLCNRLRLKFLNVTSAKAINTR
TLIMEVRQLMNCTYRPNITQRELYRVTLRSCCNATGEMILTNQNTKLGQKIHYETNQKQFKT
VDKKLHSMLPKALPWSQGLLGHCAVVGSGGILQNSSCGAAIDSADYIIRFNLGPVSNSEDVG
NNTNLMTINPSQIRTCYRNLTKDPRPLANRVAVYGNASLLLSPFSYQFSTGLSLDVYRALRP
LRPNQVLFFNPNFLLKLGRKWKERGLKESRLTSGLMLASVAMELCEEVHLYGFWPFPLDLYD
NPLPHHYYDNVGPNKGIHSMPNEFRLLLKLHTLGVLQLHLGSC

>Angang_GT29_ST8Sia7

MAAYLLKWSFLLMMITMMVHTFVVLRGTIWIVRDEMTDFQKLRCTKLREIIFSLTASKINTK
SFTEDVRALMSCPHESNITQRERHRVELRSCCNATGSLFLTRQNTREGQRIKYETNRKKNIL
VDKSIFKMLPKSTPWRNDSRFQRCAVVGNGGILRNSSCGAEIDSADIVFRLNMAPINNSRDV

GVKTSLVTINPSQIRVGYPDLQKRPQPLVERVSAYGDAPLLMPAFAYTTCTDISFKVHKVVQ
KMRPNQKVVFFNPEYLLELFQYWKHRGLEELRLTTGLMLASVAMELCDSVHLYGFWPFELDL
FQCPVTHHYYDNVGPSRRMHAMPKEFLQLLKMHCQGSIHLQLTRCH

>Ambmex_GT29_ST8Sia7-B

PVKQTHSERRSPEAMITHSKAKCQKLRRIFATLSANKTINERSIMRNAVELQRCPWLENRKK
KEFYRSEINQCCKTSHELILTQENTQVGEDIVYETQKSKKINVTTKIFNMFPKESPFSGKAY
KTCAVVGNGAILVDSCCGQQIDQAEFVFRFNLPPLNYTRDAGRRVDLVTANPSIMINRFQSL
NQRRKAFGNTLREYRGALILMAAFSHTRSTQVAFKVQYTLDDFGLGSRMLSFHPVYLENLAK
YWRSKGLYVKRLSSGIMLVSAAMELCDSITLYGFWPFYSNLEGRTIGYHYYDKMAPNIQVHA
MPKEFLIYSQMHAQGALKLQVGQCS

>Pelnig_GT29_ST8Sia7

MVRRWKVLLILSTGLAGFLFYQNLQTQHGPVHHRSKKTKTTLPPKECSHLKSLILNSVMKIF
RRKTFFEVVKALQGCPWREDQTERNLLKAKFRKCCNASYSMMVTQENTPIGHIIHYDGDKGR
KNVTESLYSLFPEKSPFQKPIRSCAVVGNGGILNSSFCGAEIDRANFVFRLNLPPMNWTNDV
GTKTDVVTANPSILIDKFGSLMERRKPFITRMKEYGSTLIILPAFSYLLNTAVSLRALYTIS
DFNLNSRAVFFNPDYLRNLTAYWKDMGIKSLRLSSGLMLVSAAIEMCDKVTLYGFWPFSQDL
DGVPIPHHYYDNALPTPKIHSMPDEFYQFLQMHIQGSLRLNLGQC

>Aptausman_GT29_ST8Sia7

MPNSISMEEYVNPGLLKMHKLWVRLLAVAAVSLFSVFFLVWSDGRPDIKVPEAEACQVLLSN
LTESFLKRVNEVWSLRHLKLMQSCRWNFNASALAQYRAELGHCCNASAWLVLTRVNTPLGTK
IVYDGDHSKSLKVSSGLLEILPEESPFQDPFYKTCAVVGNGGILLNSSCGSEIDGHQFVIRF
NLPSTDFPEDVGRKSSIVTVNPSILQERFHGLTGRRLPFVKAAAFYGETWFLIPAFSYPGQN
EASYRALYALQDSGSRSPVFFFHPQYLSALSRYWHERGFHTPRLSSGFMLVNAALELCQHIT
LYGFWPFSLHPDGHPLPHHYYDNQLPKPRVHLMPQEFAYYVNMHFQGVLHLHLGKC

>Cuccancan_GT29_ST8Sia7

MSESDATMPVSAFSKMPNSISREEHVKPGLLKKHKLWVVLLVATAASLVSMWFLVLNDRTQL
GHYCNASAWLAVTQENTPLGSNIVFDGYRSKSLKVSSGLLEILPEKSPFQDLLYKTCAVVGN
GGILRNSSCGSQIDGYQFVIRFNLPSADFPEDVGRKSSIVTVNPSILHKRFHGLNGRRLPFV
EAAASYGKTWFFIPAFSYPGNSEASYRAFYALQDSESQSHVFFFHPQYLSALSKYWHDRGFH
TYRLSSGFMLVNAALELCEHITLYGFWPFSLHPDGHALPHHYYDNVLPNQRIHIMPREFAYY
VDMHFQGVLRLHVGRC

Supplementary data2: Multiple sequence alignment of the 147 selected vertebrate ST8Sia sequences: 129 mono- α2,8-sialyltransferases *i.e.* 17 ST8Sia I, 15 ST8Sia V, 63 ST8Sia VI and 34 ST8Sia VII and 18 oligo-α2,8-sialyltransferases ST8Sia III, which are used as an outgroup were aligned using Clustal Omega in MEGA 7.0. Species name abbreviations are given in supplemental data1 legend.

```
#Mega
!Title siat8.txt;

#Takrub_GT29_ST8Sia3-B
IP-----------LTNALNEELHGKASKWKFNKTAFHQQRKDILGFIDIPNNF-------
-----------SLTKDRVRVGQLMHFDYSSHKYVFSISNNFKSLLP-------D-TSPIH
NK---HYSLCSVVGNSGILTGSHCGANIDQADFVFRCNFAPTE-VYSKDVGRKTNMTTFN
PSILERYYNNLLTIQDRNNFFLNLKKLGGAILWIPAFFLHTSATVTRTLVDFFVEHKGQL
KVELAWPGNIMHDVNKYWKNKNLSPKRVSTGIFMYTLASAMCDEIHLYGFWPFGWDPNTG
KELPYHYYDKKGTKFTTKWQETHQLPTEFKLLYKLHREGVIRLSLTHCT-----------

#Tetnig_GT29_ST8Sia3-B
IP-----------LTNALNEELHGKASKWKFNKTAFHQQRKDILGFIDIPNNF-------
-----------SLTKDRVRVGQLMHFDYSSHKYVFSISNNFKSLLP-------D-TSPIH
NK---HYSLCSVVGNSGILTGSHCGANIDQADFVFRCNFAPTE-VYSKDVGRKTNMTTFN
PSILERYYNNLLTIQDRNNFFLNLKKLGGAILWIPAFFLHTSATVTRTLVDFFVEHKGQL
KVELAWPGNIMHDVNKYWKNKNLSPKRVSTGIFMYTLASAMCDEIHLYGFWPFGWDPNTG
KELPYHYYDKKGTKFTTKWQETHQLPTEFKLLYKLHREGVIRLSLTHCT-----------

#Orylat_GT29_ST8Sia3-B
IP-----------LTTALNEELQGKPYKWKFNRTAFYQQRKDIFSYIDIPNNF-------
-----------SLTKNSVRVGQLMHFDYSSHKYVFSISNNFKSLLP-------E-ISPIL
NK---HYSVCAVVGNSGILTGSHCGPEIDQADFVFRCNFAPTD-VYSKDVGRKTNMTTFN
PSILERYYNNLLTIQDRNNFFLHLKKLEGAILWIPAFFLHTSATVTRTLVDFFVEHKGQL
KVELAWPGNIMHNVNKYWKTKNLSPKRLSTGILMYTLAFSMCDEIHLYGFWPFGWDPNTG
KDLPYHYYDKKGTKFTTKWQETHQLPSEFKLLYKLHREGVTKLGLSHCT-----------

#Gasacu_GT29_ST8Sia3-rB
IP-----------LTNALNEELQGKPSKWKFNKTAFHLQKKDIFSYIDIPTNF-------
-----------SLTKNSVRVGQLMHFDYSSHKYVFSISNNLKSLLP-------D-ASPIR
NK---HYSVCAVVGNSGILTGSHCGPEIDQADFVFRCNFAPTE-VYSKDVGKKTNLTTFN
PSILERYYNNLLTIQDRNNFFLNLKKLEGAILWIPAFFLHTSATVTRTLVDFFVEHKGQL
KVELAWPGNIMHDVNKYWKTKNLSPKRLSTGILMYTLASAMCDEIHLYGFWPFGWDPNTG
NDLPYHYYDKKGTKFTTKWQETHQLPSEFKLLYKLHRDGVIKLSLTHCS-----------

#Anocar_GT29_ST8Sia3
VP-----------NTNSLNHELQDKSPKWIFNRTAFLQQRREILQHVDVIKNF-------
-----------SLTKNMVRIGQLMHYDYSSHKYVFSISNSFRMLLP-------D-VSPIQ
NK---HYNICAVVGNSGILVGSQCGQEIDKYDFVFRCNFAPTE-AFHKDVGRKTNITTFN
PSILEKYYNNLLTIQDRNNFFLSLKKLDGAILWIPAFFFHTSATITRTLVDFFIEHRAQL
KVQLAWPGNIMQHVNRYWKNKHLAPKRLSTGILMYTLASAICDEIHLYGFWPFGFNPNNR
EDLPYHYFDKKGTKFTTKWQESHQLPAEFQQLFRMHSEGLAKLTLSHCA-----------

#Siltro_GT29_ST8Sia3
VP-----------ITNSLTQELQEKPAKWVFNKTAYARQRREILQNVDVIRNF-------
-----------SLTKNSVRTGQLMHYDYSSHKYVFSISNNFRSLLP-------D-TSPVM
NK---RYNVCAVIGNSGILTESQCGAEIDKADFVFRCNFAPTE-GFQKDVGRKTNLTTFN
PSILEKYYNNLLTIQDRNNFFLSLKKLDGAILWIPAFFFHTSASVTRTLVDFFVEHRDQL
KVQLDWPGNIMQHVNRYWKNKHLSPKRLSTGILMYTLASSVCEEIHLYGFWPFGWDPNTG
```

KDLPYHYYDKKGTKFTTKWQESHQLPAEFKLLYKMHREGLTKLTLSQCA-----------

#Bostau_GT29_ST8Sia3
VP-----------FPHSVTHELQARP-KWTFNRTAFLHQRQEILQHVDVIKNF-------
-----------SLTKNSVRIGQLMHYDYSSHKYVFSISNNFRSLLP-------D-VSPIV
NK---RYNICAVVGNSGILTGSRCGPQIDKSDFVFRCNFAPTE-AFQRDVGRKTNLTTFN
PSILEKYYNNLLTIQDRNNFFLSLKKLDGAILWIPAFFFHTSATVTRTLVDFFVEHRGQL
KLQLAWPGNIMQHVNRYWKNKHLSPKRLSTGILMYTLASAVCEEIHLYGFWPFGFDPNTR
EDLPYHYYDKKGTKFTTKWQESHQLPAEFQLLYRMHGEGLTKLTLSHCA-----------

#Galgal_GT29_ST8Sia3
VP-----------ITNSLGQELQEKPSKWVFNRTAFAQQRQEILQHVDVIKNF-------
-----------SLTKNSVRIGQLMHYDYSSHKYVFSISNNFRSLLP-------D-VSPIL
NK---HYNVCAVVGNSGILTGSQCGQEIDKSDFVFRCNFAPTE-AFQKDVGRKTNLTTFN
PSILEKYYNNLLTIQDRNNFFLSLKKLDGAILWIPAFFFHTSATVTRTLVDFFVEHRGQL
KVQLAWPGNIMQHVNRYWKNKHLSPKRLSTGILMYTLASAICEEIHLYGFWPFGFDPNTR
EDLPYHYYDKKGTKFTTKWQESHQLPAEFQLLYRMHGEGLAKLTLSRCA-----------

#Canlupfam_GT29_ST8Sia3
VP-----------ITNSLTHELQEKPSKWTFNRTAFLHQRQEILQHVDVIKNF-------
-----------SLTKNSVRIGQLMHYDYSSHKYVFSISNNFRSLLP-------D-VSPIV
NK---HFNICAVVGNSGILTGSRCGQEIDKSDFVFRCNFAPTE-AFQRDVGRKTNLTTFN
PSILEKYYNNLLTIQDRNNFFLSLKKLDGAILWIPAFFFHTSATVTRTLVDFFVEHRGQL
KVQLAWPGNIMQHVNRYWKNKHLSPKRLSTGILMYTLASAICEEIHLYGFWPFGFDPNTR
EDLPYHYYDKKGTKFTTKWQESHQLPAEFQLLYRMHGEGLTKLTLSHCA-----------

#Ratnor_GT29_ST8Sia3
VP-----------ITNFLTHELQEKPSKWTFNRTAFLHQRQEILQHVDVIKNF-------
-----------SLTKNSVRIGQLIHYDYSSHKYVFSISNNFRSLLP-------D-VSPIL
NK---RYNICAVVGNSGILTGSQCGQEIDKSDFVFRCNFAPTE-AFHKDVGKKTNLTTFN
PSILEKYYNNLLTIQDRNNFFLSLKKLDGAILWIPAFFFHTSATVTRTLVDFFVEHRGQL
KVQLAWPGNIMQHVNRYWKNKHLSPKRLSTGFLMYTLASAICEEIHLYGFWPFGFDPNTR
EDLPYHYYDKKGTKFTTKWQESHQLPAEFQLLYRMHGEGLTKLTLSHCA-----------

#Musmus_GT29_ST8Sia3
VP-----------ITNSLTHELQEKPSKWTFNRTAFLHQRQEILQHVDVIKNF-------
-----------SLTKSSVRIGQLMHYDYSSHKYVFSISNNFRSLLP-------D-VSPIM
NK---RYNVCAVVGNSGILTGSQCGQEIDKSDFVSRCNFAPTE-AFHKDVGRKTNLTTFN
PSILEKYYNNLLTIQDRNNFFLSLKKLDGAILWIPAFFFHTSATVTRTLVDFFVEHRGQL
KVQLAWPGNIMQHVNRYWKNKHLSPKRLSTGILMYTLASAICEEIHLYGFWPFGFDPNTR
EDLPYHYYDKKGTKFTTKWQESHQLPAEFQLLYRMHGEGLTKLTLSHCA-----------

#Homsap_GT29_ST8Sia3
VP-----------ITNSLTQELQEKPSKWKFNRTAFLHQRQEILQHVDVIKNF-------
-----------SLTKNSVRIGQLMHYDYSSHKYVFSISNNFRSLLP-------D-VSPIM
NK---HYNICAVVGNSGILTFIQCGREIDKSDFVFRCNFAPSE-AFQRDVGRKTNLTTFN
PSILEKYYNNLLTIQDRNNFFLSLKKLDGAILWIPAFFFHTSATVTRTLVDFFVEHRGQL
KVQLAWPGNIMQHVNRYWKNKHLSPKRLSTGILMYTLASAICEEIHLYGFWPFGFDPNTR
EDLPYHYYDKKGTKFTTKWQESHQLPAEFQLLYRMHGEGLTKLTLSHCA-----------

#Pantro_GT29_ST8Sia3
VP-----------ITNSLTQELQEKPSKWTFNRTAFLHQRQEILQHVDVIKNF-------
-----------SLTKNSVRIGQLMHYDYSSHKYVFSISNNFRSLLP-------D-VSPIM
NK---HYNICAVVGNSGILTGSQCGQEIDKSDFVFRCNFAPTE-AFQRDVGRKTNLTTFN
PSILEKYYNNLLTIQDRNNFFLSLKKLDGAILWIPAFFFHTSATVTRTLVDFFVEHRGQL
KVQLAWPGNIMQHVNRYWKNKHLSPKRLSTGILMYTLASAICEEIHLYGFWPFGFDPNTR
EDLPYHYYDKKGTKFTTKWQESHQLPAEFQLLYRMHGEGLTKLTLSHCA-----------

#Danrer_GT29_ST8Sia3
TS-----------LNTALNENLQE-SSNWRFNRSAYAELNKEIAQHIDVPHNF-------
-----------TLTKNSVRVGQLMHYDYSSHKYVFSIGENLRSLLP-------D-ASPVL

NK---RYNTCAVVGNSGILTGSRCGPEIDKYDFVFRCNFAPTE-VFRRDVGRRTNLTTFN
PSILEKYYNNLLTIQDRNNFFLSLKKLDGAILWIPAFFFHTSATVTRTLVDFFVEHKGQL
KVQLAWPGNIMQYVNKYWKTKQLSPKRLSTGILMFTLASSLCEQVHLYGFWPFGWDPNTG
KELPYHYYDKKGTKFTTKWQESHQLPTEFKLLFKMHADGVLKLSLSHCA-----------

#Orylat_GT29_ST8Sia3-A
TS-----------LSSAINEDLKN-SSKWTYNSTAFIQLRTEISHHIDMPHNF-------
-----------TLTRDAVKVGQLMHFDYSSHKYVYSIGENFRSLLP-------E-VSPIL
NR---HYNVCAVVGNSGILTGSLCGAQIEKYDFVFRCNFAPTE-IFKKDVGRRTNMTTFN
PSILEKYYNNLLTVQDRNNFFLSLKKLDGAILWIPAFFFHTSATVTRTLVDFFVEHRGQL
KVQLAWPGNIMQYINNYWKTKQLSPKRLSTGILMYTLASSMCDQIHLYGFWPFGWDPNTG
KELPYHYYDRKGTKFTTKWQESHQLPAEFKLLHKMHTEGLLKLTLSHCA-----------

#Tetnig_GT29_ST8Sia3-A
IP-----------LTSALSEDLQN-SSKWSHNGSAFTQLKKEISQYIDIPRNF-------
-----------TLTRDSVRIGQLMHYDYSSHKYVFSIGENFRSLLP-------E-VSPIR
QK---HYNVCAVVGNSGILTGSRCGQEIDSLDFVFRCNFAPTE-LFKKDVGRRTNMTTFN
PSILEKYYNNLLTVQDRNNFFLSLKRLDPAILWIPAFFFHTSATVTRTLVDFFVEPRGQL
KVQLAWPGNIMQYINNYWKTKQLSPKRLSTGILMYTLASSMCERIHLYGFWPFGWDPNTG
RELPYHYYDKKGTKFTTKWQESHQLPTEFKLLYKMHREGVLKLSLSHCG-----------

#Takrub_GT29_ST8Sia3-A
TP-----------LTSSLSEDLQN-ASKWSFNSSAFAQLKNEISQYIDVPRNF-------
-----------TLTRDSVRIGQLMHYDYSSHKYVFSIGENFRSLLP-------E-TSPIL
QK---HYNVCAVVGNSGILTGSRCGQQIDRFDFVFRCNFAPTE-IFKKDVGRRTNMTTFN
PSILEKYYNNLLTVQDRNNFFLSLKKLDTTILWIPAFFFHTSATVTRTLVDFFVEHRGQL
KVRLAWPGNIMQYINNYWKTKQLSPKRLSTGILMYTLASSMCDQIHLYGFWPFGWDPNTG
KELPYHYYDKKGTKFTTKWQESHQLPTEFKLLYKMHREGLLKLSLSHCA-----------

#Gasacu_GT29_ST8Sia3-rA
SP-----------LTNALGEDLQN-SSKWTYNSSAFLQLKNEISQYIDIPHNF-------
-----------TLTRDSVRIGQLMHYDYSGHKYVFSIGENFRSLLP-------E-VSPIL
NK---HYNVCAVVGNSGILTGSRCGPQIEKFDFVFRCNFAPTE-IFKKDVGRRTNMTTFN
PSILEKYYNNLLTVQDRNNFFLSLKKLDGVILWIPAFFFHTSATVTRTLVDFFVEHRGQL
KVRLAWPGNIMQYVNSYWKTKQLSPKRLSTGILMYTLASTMCDQIHLYGFWPFGWDPNTG
KELPYHYYDKKGTKFTTKWQESHQLPAEFKLLYKMHTQGLLKLSLSHCA-----------

#Danrer_GT29_ST8Sia7-B
------------------------------------HGCRMRLQYCCNATHSL-------
-----------ILTKRNTAVDHIITYETEGKRT-YRVKADLHKMLP-------E-DSPWS
AG---ALGLCAVVGSSGILKNSSCGRQIDSADFVIRFNLALVN--DS-DVGLKTDLMNIN
PSQI-RRYKTME--QDPGPLVERISVYGNSSLIIPAFAYGFCTSPSLRVLRVLQPIRPQ-
QPVVFFNPHYLRALDRFWKWRGLKEPRLSSGFILISAALEMCEHVHVYGFWPFGTDLQE-
NPVPYHYYDQSKPSP-----YMHRMPEEFVRLLQLHSQGALTLHLQPCHTHRAVFPNPVP

#Danrer_GT29_ST8Sia7-A
SVIKPAKSIKFKSF---AREVSDFLSCPYKSNETEQELNRIRLRFCCNATESF-------
-----------VLTKRNTAISQSIPYETNSRRT-YKMNADVHKLLP-------E-DSPWS
AG---ALGLCAVVGSSGILKNSSCGRQIDSADYVIRFNLALIN--DS-DVGLKTNLITIN
PSQI-RGYRNLE--KKPGPLVKRVSVYGNSSLIIPAFAYVICTSPSLRVLRVLQPIRPQ-
QPVLFFSPHYLRALDRFWKGRGLKERRLSTGFMLISAALEMCEHVHVYGFWPFGTDLQE-
NPIPYHYYDQMRS-G-----SVHRMPEEFLRLLQLHSQGALTLHLQPCDTH---------

#Pimpro_GT29_ST8Sia7
SIIKSAKSINFKRF---TQELSDYLSCPYKSNQTERELNRIRLQFCCNATGSF-------
-----------ILTKRNTAITQSIQYETST-KT-YKMKAELHKMLP-------E-DIPWS
GR---RLGRCAVVGSGGILKNSSCGREIDNADFVIRFNLAEVN--DS-DVGLKTDLITIN
PSQI--RYKNLQ--NNPDPLVERVSVYGNASLIIPAFAYTFCTSVSIKVLKVLQPIRPH-
QPVVFFSPSYLRSLDRFWKGRGLQEERLSTGFMLISTALELCEHVHVYGFWPFDNDLQD-
QPIPYHYYDLSRPSS-----HRHKMPEEFVRLLQLHSQGALTLHLLPCDSPETDRENNL-

#Catcat_GT29_ST8Sia7
AIIKPAKSIQLQRF---TQELSDFMSCPYKSNTTERELNRMRLQYFCNATGSL-------
-----------FLTKRNTAINQTIPYETSTVKT-YKMNAAIHGMLP-------E-DFPWS
GR---RMGRCAVVGSGGILKNSSCGREIDSADYVIRFNLASIN--DS-DVGLKTDLITIN
PSQI--QFKNLD--KNPEPLVERVSVYGNASLIMPAFAYTFCTGQSIKTLRVLHPIRPQ-
QPVVFFSPTYLRTLDRFWKGRGLKEVRLSTGFMLISTALELCEDVHVYGFWPFGTDLHN-
NSVPYHYYDQLSPHR-----YMHAMPEEFVRLLQLHSKGALTLHLQPCS-----------

#Gymprzprz_GT29_ST8Sia7
SIIKPAQSIKLESF---TQELSDFMNCPHKPNTTERELNRMRMQFCCNATGSL-------
-----------FLTKRNTAINQTIPYETSTIRT-YNMNPVIHSMLP-------E-DFPWT
GR---RLGRCAVVGSGGILKNSSCGREIDSADFVIRFNLAYIN--DS-DVGLKTDLVTIN
PSQI--RYKNLE--QNPDPLVERVSVYGNASLAMAAFAYTFCTGLSIKTLKVLHPIRPQ-
QPVVFFSPIYLRTLDRFWKGRGLKSIRLSSGFMLINTALELCEHVHVYGFWPFGTDLQN-
NSVPYHYYDELSPHR-----YKHKMP----------------------------------

#Sinrhi_GT29_ST8Sia7
SIIKPAKSLNLESF---TQELSDFMSCPYKSNTTERELNRMKLQFCCNATRSL-------
-----------FLTKRNTAVNQTIPYETSTIST-YTMNDTLHSMLP-------E-DFPWS
GR---RLGRCAVVGSGGILKNSSCGREIDSADFVIRFNLASIN--DS-DVGLKTDLVTIN
PSQIQREYKDLE--KKPNPLVKRVSVYGNASLIMPAFAYTFCTGLSIRTLKALHPIRPQ-
QPVVFFSPYYLQTLDRFWKGRGLKSIRLSTGFMLISAALEVCEHVHVYGFWPFDTDLQD-
NPVPYHYYDLRRPSK-----RMHKMPEEFVRLLQLHSQGALTLHLQPCSSDAS-------

#Sinans_GT29_ST8Sia7
SVITSVKRLNMESF---TEELVDFLKCPQKPNTTEQELNRMKLQFCCNATGSI-------
-----------FLTKRNTAINQTIPYETDTKHT-YRMNAAIHNMLP-------E-DFPWS
GR---RLGRCAVVGSGGILKNSSCGREIDSADFVIRFNLASIN--DS-DVGLKTDLVTIN
PSQIQREYKDLE--KKLNPLVKRVSVYGNTSLIMPAFAYTFCTGLSIKTLKALHPIRPQ-
QPVVFFSPYYLQTLDHFWKGRGLKSIRLSTGFMLISTALELCENVHVYGFWPFDTDLQD-
NLVPYHYYDLRRPSK-----SMHKMPEEFVRLLQLHSQGALTLHLQPCSSDAS-------

#Lepocu_GT29_ST8Sia7
LGMQSLKKVNSTLL---CGQVTQLMRCPWTLNLTQLEQQRTELRLSCNATGRL-------
-----------VVTQDNTPLGGTITYDAEKWT--RQVDTEIFNMLP-------Q-SPPWA
GGPGARLRRCAVVGNGGILRNSSCGAHIDRADFVIRFNLPPLN--YSRDVGVKSSLVTVN
PSQIINSYRNLN--YARRPFVQRVSTYENAHLVLPAFAFSFCTDPCFRVYYTLQDNRPH-
QRALFYHPDYLRQLATYWQEKGLRETRLSTGLMMVSAALELCDRVELYGFWPFSFDLSE-
RPLSNHYYDDVPPNR-----GMHAMPEEFLRLLRMHSQGVLRLRVGQCP-----------

#Angang_GT29_ST8Sia7
FSLT-ASKINTKSF---TEDVRALMSCPHESNITQRERHRVELRSCCNATGSL-------
-----------FLTRQNTREGQRIKYETNRKKN-ILVDKSIFKMLP-------K-STPWR
ND--SRFQRCAVVGNGGILRNSSCGAEIDSADIVFRLNMAPIN--NSRDVGVKTSLVTIN
PSQIRVGYPDLQ--KRPQPLVERVSAYGDAPLLMPAFAYTTCTDISFKVHKVVQKMRPN-
QKVVFFNPEYLLELFQYWKHRGLEELRLTTGLMLASVAMELCDSVHLYGFWPFELDLFQ-
CPVTHHYYDNVGPSR-----RMHAMPKEFLQLLKMHCQGSIHLQLTRCH-----------

#Salsal_GT29_ST8Sia7-B
VTMTSMKKVDIALF---SQEVRELMNCPWRLNITHRELHRTELWSCCNASDRL-------
-----------MVTRQNTNQNQSLTYEVERRMK-RKVDQALWEMLP-------Q-TVPWS
KG---SLSRCAVVGSGGILQNSSCGAEIDSADYIIRFNLGPVT--NSKDVGNKTHLMTIN
PSQIR-GYRNLT--KAPQPLANRVAVYGNASLLLPAFSYQFSTGLSLDVYHALQPLRPY-
QKVVFFNPNFMLNLGRKWKGQGLKEPRLSTGLMLASVAMELCEEVHIYGFWPFSLDLNH-
NPLPHHYYDNVGPKI-----GFHSMPEEFQLLLKLHTQGALQLHLGRC------------

#Salsal_GT29_ST8Sia7
LTLTSAKAINMRTL---IMDVRQLMTCTHRPNITQRELYRVILRSCCNATGEM-------
-----------ILTKQNTKLGQKIHYETNQKLF-KTVDKKLHSMLP-------K-DLPWS
KG---LLSHCAVVGSGGILQNSSCGAEIDSADYIIRFNLGPVT--NSKDVGNKTHLMTIN
PSQIR-GYRNLT--KAPQPLANRVAVYGNASLLLPAFSYQFSTGLSLDVYHALQPLRPY-

```
QKVVFFNPNFMLNLGRKWKGQGLKEPRLSTGLMLASVAMELCEEVHIYGFWPFSLDLNH-
NPLPHHYYDNVGPKI-----GFHSMPEEFQLLLKLHTQGALQLHLGRC------------

#Salfon_GT29_ST8Sia7
-------SINTRTL---IMDVRELMNCPHRPNITQRELYRVILRSCCNATGEM-------
-----------ILTNQNTKSGQKIHYETNQKLF-KTVDKKLHSMLP-------K-ALPWS
KG---LLGHCAVVGSGGILQNSSCGAEIDSADYIIRFNLGPVT--NSKDVGNKTHLMTIN
PSQIR-GYPNLT--KDPQPLANRVAVYGNASLLLPAFSYQFCTGLSLNAYHALQPLRPN-
QKVVFFNPNFMLNLGRKWKGQGLKEPRLSTGLMLASVAMELCEEVHIYGFWPFSLDLNH-
NPLPHHYYDNVGPKK-----GVHSMPDEFQLLLKLHTQGVLQLHLGRC------------

#Corclu_GT29_ST8Sia7
LTVTSAKAINPRTL---IMDIRQLMDCSHRPNITQRELYRVILRSCCNATGEM-------
-----------ILTNQNTKLGQKIHYETNQKLF-KTVDKKLHSMLP-------N-ALPWS
KG---LLGRCAVVGSGGILQNSSCGAEIDSADYIIRFNLGPVS--NSEDVGNKTHLMTIN
PSQIRTGYRNLT--KDPQPLANRVAAYGNASLLLSPFSYQFCTGLSLDVYHVLRPLRPN-
QKVVFFNPNFLLQLSRKWKGRGLKEPRLTSGLMLASVAMELCEEVHLYGFWPFPLDLYH-
NPLPHHYYDNVGPKK-----GVHSMPDEFLLMLQLHTQGVLQLHLGGC------------

#Thythy_GT29_ST8Sia7-B
LNVTSAKAINTRTL---IMEVRQLMNCTYRPNITQRELYRVTLRSCCNATGEM-------
-----------ILTNQNTKLGQKIHYETNQKQF-KTVDKKLHSMLP-------K-ALPWS
QG---LLGHCAVVGSGGILQNSSCGAAIDSADYIIRFNLGPVS--NSEDVGNNTNLMTIN
PSQIRTCYRNLT--KDPRPLANRVAVYGNASLLLSPFSYQFSTGLSLDVYRALRPLRPN-
Q-VLFFNPNFLLKLGRKWKERGLKESRLTSGLMLASVAMELCEEVHLYGFWPFPLDLYD-
NPLPHHYYDNVGPNK-----GIHSMPNEFRLLLKLHTLGVLQLHLGSC------------

#Esoluc_GT29_ST8Sia7
VAMTTMKKVDIVPF---SQVVRELMDCPWRLNLTHRELHRTELWACCNASDRL-------
-----------IVTRQNTHVNQTLTYEAERWRK-KLVDQTLWEMFP-------K-NVPWS
KG---LLGRCAVVGSGGILQNSSCGEEIDRAHYIIRFNLAPVN--QSRDVGLKTDLITAN
PSQIIKQYPDLH--KNPGRLAEQMSVYGNARLLLPAFSFAFGTAPCFKVYHALQKALPQ-
QEVVFFHPDYLFELGQFWRRRGQKASRISTGLMLASSALEICDQVHLYGFWPFSVDLSH-
NPLQHHYFDNVGPNH-----YMHAMPEEFLLLLQLHSQGALQLHVGHCR-----------

#Oncmyk_GT29_ST8Sia7
VTMSTIKKVDIALF---SQDVRELMDCPWRLNLTHRELYRTELWSCCNASDRL-------
-----------MVTRQNTNLNQSLSYEIHRWKK-RKVDQALWEMLP-------Q-TVPWS
KG---SLSRCAVVGSGGILQNSSCGAEIDNADYVIRFNLAPIN--KSCDVGVKTDLVTAN
PSQIIKGYTDLQ--QNPGHLAEVLSFYGHAHLLLPAFSSASGTAPCFKVYHALRKARPQ-
QEVVFFHPDYLFELGRFWRHRGQRARRLSTGLMLASTALEICEQVHLYGFWPFPLDLSH-
NSLPHHYYDNVGPNR-----KMHAMPEEFLLLLQLHSQGALQLHVGPCTLTPTP------

#Thythy_GT29_ST8Sia7
VTMTTMKKANIALF---SQEVRELMDCPWRLNLTHRELHRTELWSCCNASDRL-------
-----------MVTRQNTNQNQILTYEAEKWRK-RKVDKALWEMLP-------Q-TVPWS
KG---SLSRCAVVGSGGILQNSSCGAEIDTADYVIRFNLAPIN--KSCDVGVKTDLITAN
PSQIIKGYHNLQ--RNPGPLVKRMSVYGHAHLLLHAFAFGFGTSPCFKVYHALRKARPQ-
QEVVFFHPDYLLQLDRFWRRRGQRAPRLSTGLMLASTALEICEQVHLYGFWPFHLDLSQ-
NTLPHHYYDNVGPSR-----FMHAMPEEFLLLLQLHSQGALQLHVGPCTL----------

#Takrub_GT29_ST8Sia6-A
------------EI---TDKVNRLYAQTLEKKEDKSKILRSELSSKCQGFEKA-------
-----------IITQANTVVGSKIVYDGERKSL-VQVTPEMFNTFP-------K-EHPFP
NK---TWETCAVVGNGGILSDSGCGKMIDSAQFVIRCNLPPLNNGYQDHVGVKTDLVTAN
PSILVEKYGALM--GRRRPFIESLRSYGNSLLLLPTFSYRSNTPVSLRAFYSIEDFGSP-
TRAISFNPQYLQKLDVFWRSKGLRAVRLSSGLMVASLALELCSNVHLYGFWPFSNHPNGL
RTLKNHYYDDIPPKK-----KFHAMPVEFELLLKLHTEGVLRLHLDDCVPGEVNLMGSTG

#Takrub_GT29_ST8Sia6-B
------------EI---IDKVNRLYAQTWEKKEDKYKKFRSELSSKCQGFEKA-------
```

-----------IITQANTVVGSKIVYDGERKRS-VKVSTEMFNTFP-------K-EHPFP
NK---TWETCAVVGNGGILSDSGCGKMIDSAQFVIRCNLPPLNNGYQDHVGVKTDLVTAN
PSILFEKYGALM--GRRRPFIESLRSYGNSLLLIPAFSYGNNTPVSLRAFYSIEDFGSP-
TRTIFFNPQYLQKLAVFWRSKGLRAVRLSSGLMVASLALELCSNVHLFGFWPFSNHPHGL
RTLKNHYYDDIQTKK-----KFHAMPVEFELLLQLHTEGVLRLHLDDCVPGEVNLMGSTR

#Orylat_GT29_ST8Sia6
------------EN---IMKALELYSEPWKKQEDNYHQFRSQLNSKCHGLEKA-------
-----------IITQANTPQGTKLVYDAERKRT-LVVNAEVFNTFI-------K-ENPFP
NK---TWDTCAVVGNGGILANSSCGKTIDSAQFVIRCNLPPLSNGFEKDVGIKSDIVTAN
PSIITEKYFSLM--RHRRPFAEAMRIYGNSMVLLPAFSFGHNTALSMRAFYTLEDFESS-
ARAVYFNPEYLKNLANFWRSEGLKSPRLSTGIMMASIALEVCSEVHLYGFWPFDVHPYSH
QGLTNHYYDDRKAKN-----KFHAMPTEFNLLLQLHRKGVLRLHLGDCTPDEK-------

#Notfur_GT29_ST8Sia6
------------ED---IKKALELYSQTWKKQEDSYQNFRSLLNRKCKCFDKA-------
-----------IITQNNTPLGSKLVYDGERKRT-LQVNQEIFNTFP-------K-GHPFS
NK---TLHTCAVVGNGGILANSSCGKTIDSAEFVIRCNLPPLSNGYEKHVGIKTHLVTAN
PSILMEKYAALM--ARRRPFVENLRSYGDSMLLLPAFSYGRNTPVSLRAFYTLEDFESP-
IQSIFFNPAYLRNLAAFWRSQGLKAVRLSTGIIMTSLALEICENVHLYGFWPFGVHPYSS
QDLTNHYYDDRKTKI-----KFHAMPDEFNLLLNLHSQGVLKMHLGDCEPDEKRFHRSD-

#Orenil_GT29_ST8Sia6-B
------------EI---ISKVQERYNQTWKKQEDNYLKFRTNLSVKCNGFDKA-------
-----------IITKNNTPVGLKLVYDGEKKRT-LQVNKDFFNIFT-------K-ENPFS
NK---KWDTCSVVGNGGILSESSCGKMIDSADFVIRCNLPPLENGYEKDVGIKTSLVTAN
PSIFTQRYGSLV--GRRLPFVESLHKYGNSLLLLPAFSFGINTAVCQRVAYTIEDFKSP-
IRPVFFNPQYLDSLAQFWRSEGLKERRLSTGLIMASMALELCENVHLYGFWPFSNHPYGF
YTLTNHYYDDKPAKT-----SFHAMPAEFDRLLQLHTEGVLRLHLEDCK-----------

#Gasacu_GT29_ST8Sia6
------------QF---IDKVRERYNKTWIKQVDDYLEFRSQLSRKCHGFDSA-------
-----------FITQYNTPVGAKIVYDGEKTRT-LQVTPEIFSTFP-------K-EHPFS
NK---IWGTCSVVGNGGILSNSSCGKMIDSAEFVMRCNLPPLDNGYENDVGIKTDLVTAN
PSILIKKYGSLQ--QRRRPFVESLRSYGNSLLLLPAFSYGFNTPLSLRAVYSIEDFKSP-
TRPVFFNPEYLESLGLFWRSRGLKAVRASTGLMMASLALEHCTDVHLYGFWPFGNHPQGL
HALTNHYYDDVQPKN-----TVHAMPVEFEFLLQLHSQGVLRLHLEDCQPGEK-------

#Anofim_GT29_ST8Sia6
------------EV---INKVIERYSKTWKKQEENYQKFRSQLSSKCHGFDKA-------
-----------IITQANTPVGAKLVYDGEKKRT-LQVTPEIFSTFA-------K-EHPFQ
NK---IWDTCAVVGNGGILTNSSCGKTIDSAQFVMRCNLPPLDKGYEKHVGIKTDLVTAN
PSILLEKYGALM--GRRRPFVESLRSYGNSLLLLPAFSFGFNTPVCLRAVYSIEDFESP-
TRPVFFNPEYLQKLALFLRSQGLRAPRPSTGIIMASLALEHCANVHLYGFWPFSSHPHGL
HALTNHYYDDRQTKK-----KFHSMPAEFDLLLQLHSQGVLRLHLGDCPPGER-------

#Larcro_GT29_ST8Sia6
------------EV---IDKVMERYSQSWERQEDNYQKFRAQLNNKCHGFDKA-------
-----------IITQANTPVGSKLAYDGEKKRI-LQVTPEIFSTFA-------K-ERPFP
NK---TWDTCAVVGNGGILTNSGCGKVIDSAQFVIRCNLPPLENGYEKHVGIKTDLVTAN
PSIFLEKYGGLM--GRRRPFVESLHSYGNSLLLIPAFSYGHNTPVSLRVAYSLEDFKSS-
TRPIFFNPEYLHSLALFWRSQGLRAVRLSTGIIMASLALELCANVDLYGFWPFSNHPHGF
HTLTNHYYDDRQTKV-----KFHAMPAEFELLLRLHSQGVLRLHLGDCQPSEK-------

#Ampoce_GT29_ST8Sia6
------------EV---IDKVVARYSQPWKRQEDSYQKFRFQLSSKCHGFNKA-------
-----------IITKTNTPVGSKLVYDGEKKRT-LQVTPEIFSTFI-------K-DHPFS
NK---TWDTCAVVGNGGILTNSSCGKTIDSAQFVIRCNLPPLANGYEKHVGVKTDLVTAN
PSILLEKYGALM--GRRRPFVESLRTYGDSLLLLPAFSFGHNTPVSLRAAYTIEDFESP-
TRPVFFNPEYLHSLALFWRSQGLKAVRLSTGLIMASLAVELCSNVHLYGFWPFSNHPHGL
QPLTNHYYDNRQTKT-----KFHAMPAEFDLLLRLHSQGVLRLHLGDCPTSGK-------

#Serlaldor_GT29_ST8Sia6
------------EI---IDNVIERYSQTWKKQEDNYQKFRSQLNSKCRGFNKA-------
-----------IITQANTPVGSKLVYDGEKRRS-LQVTPEIFSTFV-------K-EHPFS
NK---TWGTCAVVGNGGILTNSSCGQTIDSAQFVIRCNLPPLKNGYEKHVGNKTDLVTAN
PSILVEKYGALM--AHRRPFVDSLHTYGNSLLLLPAFSFGHNTPVSLRAAYTIEDFGSP-
IKPVFFNPEYLQRLAVFWRSQGLRAVRLSTGIIMASLALELCADVHLYGFWPFSTHPHGF
HPLTNHYYDDRKTKM-----KFHAMPAEFDLLLRLHSQGVLRLHLGDCRPHEK-------

#Paroli_GT29_ST8Sia6
------------EI---IDKVIQRYSQTWKRQEDNYQKIRSQLSSKCHGFDKA-------
-----------IITQANTPVGSKLVYDGEKKRT-LQVTPEVFSTFI-------K-ERPFS
NK---TWDTCAVVGNGGILTNSSCGQMIDSAQFVIRCNLPPLENGYEKHVGTKTDLVTAN
PSILMEKYGALM--GRRRPFVESLRIYGDSLLLIPAFSYGHNTPVSLRVVYTIEDFESP-
TRPIFFNPEYLQSLAVFWRSQGLKAVRLSTGIIMASLALELCSNVHLYGFWPFSNHPHGL
HVLNNHYYDDRKTKT-----KFHAMPAEFDLLLRLHSQGALRLHLGDC------------

#Lepocu_GT29_ST8Sia6
----------EKAN---IDKLAQVHSQTWRKQESRLQTFRAQLNMKCQGFSRA-------
-----------IITQANTLLGSKVTYDGERRKP-VEVTPKLYSTFP-------K-EHPFG
NV---SFQSCAVVGNGGILANSSCGEEIDGAQFVIRCNLPPVDRKYQDDVGNKTDLVTAN
PSILLERFEGLM--ELRRPFVESLGDYGQPMLALPAFSYGHNTPVSLRAVYTLQDFNSP-
VRAVFLNPEYLQNLARFWKAHGLRTVRLSTGLIVASLALELCASVTLYGFWPFSLHPFSK
QHLTNHYYDDQQSKK-----SFHAMPAEFEQLLRLHSQGVIRVHLGSC------------

#Amical_GT29_ST8Sia6
----------EQSV---IDRLLQTHIVDWEKQEHKFQSFRAQLNSRCDGLSKA-------
-----------IISQTNTPLGSKVVYDGEKRKP-IQVTPKLFSTFP-------K-EHPFG
NR---TFTSCAVVGNGGILMNSSCGGEIDEAQFVIRCNLPPVDHGYQRDVGNKTSLVTAN
PSILLDKFNGLM--ELRRPFVDSLGSYGDPLLVLPAFSYSRNTPVSLRALYTLQDFDSP-
VRPVFLNPEYLHNLARFWKAQGLQAIRLSTGLIMASLALELCANVQLYGFWPFSKDPHSK
HPLTNHYYDDQQAKK-----TIHAMSTEFSHLLMLHNQGIIRVQLGKCQAGTRRATPSLH

#Angang_GT29_ST8Sia6
----------EY-T---IDKMVELYSYAWKKQNANFNKFRSQLSSRCRGVSTA-------
-----------IVTQNNTPLDSKIYYDGEKRKP-LQVTPKLYSTFA-------K-EQPFE
NV---TWKTCAVVGNGGILVNSSCGEAIDSAHFVFRCNLPPLDKAYQKDVGNKTNLVTAN
PSILIEKFEGLM--EYRRPFVESLSDYGEALLLLPAFSYAHNTPVSLRAFYTLRDFGGR-
ARPAFLSPAYLQSLAHFWRAQGLRTVRLSTGLIVASLALELCANVHLYGFWPHAQHPHDR
RPLTNHYYDDRQGKK-----KVHAMPAEFGHLLRLHRQGVVRVHLGQCEDRPR-------

#Cluhar_GT29_ST8Sia6
----------DS-V---IDKVLERYSKNWKKQENNYKRFRALLSSRCHGATKA-------
-----------VVTQANTPLGSKVVYDGEKRKP-LQVTSALFNVFP-------K-EPPFG
NT---SWDTCSVVGNGGILANSSCGRRIDSAQFVIRCNLPPLEHGYEEDVGNKTDLVTAN
PSILHEKFGGLM--ERRRPFVEGLRPYGNSLILLPAFSYSHNTPVSLRAVYTLEDFRSP-
ARPVFLSPDYLTSLARFWRSQGLRSVRLSTGLIVASLALELCTNVDLYGFWPFSQHPHGH
QPLTNHYYDNRETKK-----KIHAMPAEFDHLLRLHGQGILRVHLGQCPRSDG-------

#Esoluc_GT29_ST8Sia6
----------DTTI---IEKVVEHYSHSWKKQEDNFRKFRSLLRNTCHGLTKA-------
-----------VVTQSNTPLGSKVVYDGEKRKP-LQVTAELFSTFA-------K-EHPFV
NA---TWDTCSVVGNGGILANSSCGERIDSAQFIIRCNLPPLANGYERDVGNKTDLVTAN
PSILQEKYKGLQ--EHRLPFVKSLHSYGDSFVLLPAFSYGHNTPLSLRALYTIQDFNSP-
SRPIFLNPEYLRSLARFWRSQGLKTARLSTGLIVASLALELCANVHLYGFWPFSQHPQHH
RPLTNHYYDDRQSKK-----TVHAMPAEFDQLQRLHNQGVLRLHLGECAPAAT-------

#Onckis_GT29_ST8Sia6-A
----------DT-V---TDKVVELYSHSWKKQEDKFRNFRSLLSNRCNGLTKA-------
-----------MVTQANTPLGSKVVYDGEKRKP-LQVTPELFSTFA-------K-ENPFV
NT---TWDTCSVVGNGGILANSSCGEKIDSAQFVIRCNLPPLENGYERDVGNKTDLVTAN

PSILMEKYGGLQ--ERRRPFVESLRSYGDSLMLLPAFSYGHNTPVSLRALYTIQDFDSP-
SRPVFLNPEYLQSLARFWQGQGLRTVRLSTGLIVASLALELCANVHLYGFWPFNEHPHRH
QPLTNHYYDDRQSKK-----TVHAMPAEFDHLLRLHTQGVLRIHLGECAPTARCRPSSPL

#Salalp_GT29_ST8Sia6
----------DT-V---TEKVVERYSHSWKKQEDKFRNFRSLLSNRCHGLTKA-------
-----------MVTQANTPLGSKVVYDGEKRKP-LQVTPELFSTFA-------K-ENPFV
NT---TWDTCSVVGNGGILANSSCGERIDSAQFVIRCNLPPLENGYERDVGNKTDLVTAN
PSILMEKYGGLQ--ERRRPFVESLRSYGDSLMLLPAFSYGPNTPVSLRALYTIQDFDSP-
LRPVFLNPEYLQSLAHFWQGQGLRTVRLSTGLIVASLALELCANVHLYGFWPFNEHPHRH
QPLTNHYYDNRQSKK-----TVHAMPAEFDHLLRLHSQGVLRIHLGECAPAAR-------

#Salsal_GT29_ST8Sia6
----------DT-V---TEKVVERYSHSWKKQEDKFRNFRSLLSNRCHGLTKA-------
-----------MVTQANTPLGSKVVYDGEKRKP-LQVTPELFSTFA-------K-ENPFV
NT---TWDTCSVVGNGGILANSSCGEKIDSAQFVIRCNLPPLENGYERDVGNKTDLVTAN
PSILMEKYGGLQ--ERRRPFVESLRSYGDSLMLLPAFSYGHNTPVSLRALYTIQDFDSP-
SRPVFLNPEYLQSLARFWQGQGLRTVRLSTGLIVASLALELCANVHLYGFWPFNKHPHRH
QNLTNHYYDDRQSKK-----TVHAMPAEFDHLLRLHTQGVLRIHLGECTPTASPGCSS-K

#Oncmyk_GT29_ST8Sia6
----------DT-V---TDKVLELYSHSWRKQEDKFRKFRSLLSNRCHGLTKA-------
-----------MVTQANTPLGSKVVYDGEKRKP-LQVTPELFSTFA-------K-ENPFV
NT---TWDTCSVVGNGGILANSSCGEKIDSAQFVIRCNLPPLENGYERDVGNKTDLVTAN
PSILMEKYGGLQ--ERRRPFVESLRSYGDSLMLLPAFSYGHNTPVSLRALYTIQDFDSP-
SRPVFLNPEYLQSLARFWQGQGLRTVRLSTGLIVASLALELCANVHLYGFWPFNEHPHRH
QPLTNHYYDNRQSKK-----TVHAMPAEFDHLLRLHTQGVLRIHLGECAPTASPGCSS-K

#Onctsh_GT29_ST8Sia6
----------DT-V---TDKVVELYSHSWRKQEDKFRNFRSLLSNRCHGLTKA-------
-----------MVTQANTPLGSKVVYDGEKRKP-LQVTPELFSTFA-------K-ENPFV
NT---TWDTCSVVGNGGILANSSCGEKIDSAQFVIRCNLPPLENGYERDVGNKTDLVTAN
PSILMEKYGGLQ--ERRRPFVESLRSYGDSLMLLPAFSYGHNTPVSLRALYTIQDFDSP-
SRPVFLNPEYLQSLARFWQGQGLRTVRLSTGLIVASLALELCANVHLYGFWPFNEHPHRH
QPLTNHYYDDRQSKK-----TVHAMPAEFDHLLRLHTQGVLRIHLGECAPTASPGCSS-K

#Danrer_GT29_ST8Sia6
----------DSVI---IGKALGNYSNSWKKHEANYKRFRLLLNEKCHAVSKA-------
-----------VVTQNNTPLGSNVVYDGERRKP-LQVTQALYNILA-------K-EQPFG
NA---TWESCAVVGNGGVLANSSCGEEINSAQFVIRCNLPPLDDRYEKDVGNKTNLVTAN
PSILHEKYSGLM--ERRRPFVESLHSYGQALLLLPAFSYGHNTPVSLRAFYTLEDFGREG
PLPIFLNPEYLRKLTKFWREQGLNSVRPSTGLIMASLALEICTNVHLYGFWPFGKHPNDS
RPITNHYYDNRESKK-----NVHSMPSEFEQLLKLHKQGVVHIHLGECQPAHR-------

#Cypcar_GT29_ST8Sia6
----------DDVL---IAKALENYSDKWKKQETNFKKFRSLLSNKCHAVSKA-------
-----------VVTQNNTPLGSNVIYDGERRKP-LHVTQALFNILA-------K-EQPFG
NA---TWESCAVVGNGGILANSSCGEEINSAQFVIRCNLPPLDKGYEKDVGNKTNLVTAN
PSILHEKYSGLM--ERRRPFVESLRPYGQALLLLPAFSYGHNTPVSLRAFYTLEDFGSNS
PLPVFLNPDYLRRLSKFWRERGLNSVRPSTGLIVASLALEICSNVHLYGFWPFSKHPYDS
QAITNHYYDNRESKK-----NVHSMPTEFEYLLKLHNQGVIRIHLGKCQPTHS-------

#Sinans_GT29_ST8Sia6-B
----------DSVL---IAKALENYSDKWKKHEANLKRFRSLMSSKCHAVSKA-------
-----------VVTQNNTPLGSNVIYDGERRKP-LQVTQALFNILA-------K-EQPFG
NA---TWESCAVVGNGGILANSSCGEEINSAQFVIRCNLPPLDKGYEKDVGKKTNLVTAN
PSILHEKYSGLM--ERRRPFVESLRPYGQALLLLPAFSYGHNTPVSLRAFYTLEDFGSNS
PLPVFLNPEYLRRLSKFWREQGLNSVRPSTGLIVASLALEICSNVHLYGFWPFSKHPYDS
QPITNHYYNDRESKK-----NVHSMPNEFAHLLKLHKQGVIRIHLGECQPTHS-------

#Sinans_GT29_ST8Sia6

```
----------DNVL---IAKALENYSHKWKKHEANFKRFRSLLSSKCHAVSKA-------
-----------VVTQNNTPLGSNVIYDGERRKP-LQVTQALFNILA-------K-EQPFG
NA---TWESCAVVGNGGILANSSCGEEINSAQFVIRCNLPPLDNRYEKDVGNKTSLVTAN
PSILHEKYSGLM--ERRRPFVESLRPYGQALLLLPAFSYGHNTPVSLRAFYTLEDFGSDS
PLPVFLNPEYLRRLSKFWRERGLNSVRPSTGLIMASLALEICSNVHLYGFWPFNKHPNDS
RPITNHYYDDRESKK-----NVHSMPTEFEHLLKLHKQGVIRIHLGECQPT---------

#Sinrhi_GT29_ST8Sia6
----------DNVL---IAKALENYSHKWKKHEANFKRFRSLLSSKCHAVSKA-------
-----------VVTQNNTPLGSNVIYDGERRKP-LQVTQALFNILA-------K-EQPFG
NA---TWESCAVVGNGGILANSSCGEEINSAQFVIRCNLPPLDNRYEKDVGNKTSLVTAN
PSILHEKYSGLM--ERRRPFVESLRPYGQALLLLPAFSYGHSTPVSLRAFYTLEDFGSDS
PLPVFLNPEYLRRLLKFWRERGLNSVRPSTGLIMASLALEICSNVHLYGFWPFNKHPNDS
RPITNHYYDDRESKK-----NVHSMPTEFEHLLKLHKQGVIRIHLGECQPT---------

#Pygnat_GT29_ST8Sia6
----------ENVI---IDKALRVYSSRWRRQEANFKRFRSLLSRNCHALSKA-------
-----------VVTQANTPVGSKLVYDGEKTKP-LQVTSALFNTFA-------K-EQPFG
NA---TWDTCAVVGNGGILANSSCGEKINSADFIIRCNLPPLGSGYEKDVGNQTSLVTAN
PSILIEKFGGLM--ERRRPFVESLRPYGDSLLVLPAFSYGHNTPVSLRAFYTLEDFGITS
ARPVFLNPEYLSSLARFWRGQGLRSARLSTGLIVASLALELCTNVHLYGFWPFSQHPYGR
QPITNHYYDDRQSKK-----NVHSMPAEFDHLLRLHMQGVIQMHLGACSMLNRTSN----

#Astmex_GT29_ST8Sia6
----------DNAI---IDRLLKSYSPRWKRREANFSKFRSLLSSSCHAVSKA-------
-----------VVTQSNTPVGSKVVYDGEKTKP-LQVTKALFSTFA-------K-EQPFG
NA---SWDTCAVVGNGGILVNSSCAEKINSANLVIRCNLPPLGNGYEKDVGNKTSLVTAN
PSILIEKFSGLM--ERRRPFVESLRPYGDSLLLLPAFSYSHNTPVSLRALYALEDFNAVG
PRPVFFNPEYLSSLARFWRGRGLRTARLSTGLIVTSLALELCTNVHLYGFWPFSLHPHGR
QTITNHYYDNRQSKK-----NVHSMPSEFEHLLRLHVSGVIQLHLGECSTTDLNHTEKT-

#Danrer_GT29_ST8Sia1
---------SDKEMV--EEVLR--QGQTWSRNQTAVELYRKLLTDCCNPKRMF-------
-----------AVTKENSPLGKVLWYDGEFYHY-HTVTNETYPIFV-------Q-DTPLQ
L----PLKRCSVVGNGGVLKHSGCGNEIDRADFIMRCNLPPLSKDYTDDVGTKTHLVSAN
PSIIEKSFQNLL--WSRKSFVESMKAYGSSYIYIPAFSMKPGTDPSLRAYHALADSSSN-
QTVLFANPDFLKNVGIFWKNHGVHGKRLSTGLFLVSLALGLCEEVTAYGFWPFSVGLDE-
RPVSHHYYDNILPSS-----RFHAMPEEFLQLWHLHKSGTLRMRVGSCAKERMELKREK-

#Orylat_GT29_ST8Sia1
---------RDKDIV--AEVLR--QGDVWRKNQTGIDLYRDLLTKCCNPKKMF-------
-----------AVTKENSPIGKVLWYDGEIYHS-HTVNSETYQLFI-------K-RRGAA
S----SCRRCAVVGNGGILKGSKKGKEIDHHHYIFRCNLPPLSREYVEDVGTKTHLVTAN
PSIIEKRFQNLV--WSRKTFVDSMKVYGSSYIYMPAFSMAPGTDPSLRAYFALTDVPSN-
LTMLFANPDFLLSVSKFWKAHGVHARRLSTGLFLVSLAMGLCDEVTTYGFWPFSVDLDE-
QPISHHYYDNILPYK-----WFHAMPEEFVQLWHLHKSGILRMKVGHCSPQN--------

#Gasacu_GT29_ST8Sia1
---------RDKDIV--EEVLK--QGGVWQKNQTGIDLYRKLLTECCDPRRMF-------
-----------AVTKENSPTGKVLWYDGEFYHS-HTVNNETYPLLV-------K-DNPLR
L----PLKKCAVVGNGGILRHSQCGRDIDQADFVLRCNLPPLSKEYVDDVGTRTHLVTAN
PSIIEKRFQNLL--WSRKVFVDSMKVYGSGYIYMPAFSMKTGTDPSLRAYHALADSSSN-
LTMLFANPEFLRTVGRFWKARGVHARRLSTGLFLVSLALGLCEEVTAYGFWPFSVGLDE-
QPVSHHYYDNILPYT-----WFHAMPEEFVQLWHLHKSGALRVRVGRCSAQGGGG-----

#Takrub_GT29_ST8Sia1
---------ADKEIV--DEVLR--QGEAWQKNQTGIDLYRKLLTECCDPPRMF-------
-----------AVTKQNSPLGKVMWYDGEFYHS-HTVNNETYPLFV-------Q-ENPLQ
L----PLKKCAVVGNGGILRGSKCGKDIDRADFIMRCNLPPLSKEYLEDVGTKTHLVTAN
PSIIENRFQNLL--WSRKHFVDSMKVYGTSYIYMPAFSMSPGTNPSLRAYYALADASAN-
LTMLFANPEFLRSVGKFWKARNVHAKRLSTGLFMVSLALALCKEVTVYGFWPFAIDLDE-
```

```
QPVSHHYYDNILPYK-----WFHTMPEEFVQLWHLHKSGTLRMRVGRCPPEDGGR-----

#Tetnig_GT29_ST8Sia1
---------GDKEIV--AEVLR--QGEAWHRNQTGIDLYRRLLTDCCDPPKMF-------
-----------AVTKRNSPLGKVMWYDGEFYHS-HTVNNETYRLFV-------Q-DNPLQ
L----PLKKCAVVGNGGILRASKCGKDIDQADFIMRCNLPPLSEEYLEDVGTKTHLVTAN
PSIIENRFQSLL--WSRKHFVDSIKVYGTSYIYMPAFSMRPGTEPSLRAYYALADTSSN-
LTVLFANPEFLRTVGKFWKARNVHAKRLSTGLFMVSLALALCEEVTVYGFWPFAIDLDE-
QPVSHHYYDNILPYK-----WFHTMPEEFVQLWHLHKSGTLRMRVGRCSSQGGGR-----

#Calmil_GT29_ST8Sia1
---------DQKEIV--NDVLH---QPVWRRNQTATTAFRRLLENCCETKRMF-------
-----------AVTQHNSPMGKNLWFDGEFLHS-LSVNNEMFAMFP-------Q-DTPFQ
I----PLKKCSVVGNGGILKRSACGDKIDKADFVMRCNLPPLSNEYARDVGHRTQLVTAN
PSIIKKRYQNLM--WSRKAFVESMKVYGRSYIYMPGFSMKAGTEPSFRTYYTLSDVNAN-
QTIIFANPDFLRNVQKFWKGKGVHAKRLSTGLFLVSVALGLCEEVTLYGFWPFSTDLEE-
RTISHHYYDNVMPDT-----SYHAMPEEFLQLWLLHKSGILKIHVGKCGQIPM-------

#Xenlae_GT29_ST8Sia1
---------GHKEMI--RQVL--QFGPRWGRNRSSGDSFRKLLQDCCDPPRLF-------
-----------SMTKANTALGENLWYDGEFFQS-LTIDNTTRSLFP-------Q-DTPIK
L----PLKRCSVVGNGGILKNSRCGEQIDEADFVMRCNLPPLSREYTDDVGTKTQLVTVN
PSIIDKRFQNLL--WSRKSFVESVSVYKQSYVYMPAFSTKRGTDPSLRVYYTLEDFGTN-
QTVLFANPNFLRNVGKFWKSKGVHSKRLSTGLFMVSAALSLCEEVTIYGFWPFQMDLGG-
RHISHHYYDNMLPLS-----GVHAMPEEFLQLWHLHKSGVLQMQLDQCKKDVSSKKPH--

#Siltro_GT29_ST8Sia1
---------GHKEMV--REVL--RFGPGWRKNRTEMDSFRKLLQDCCDPPHLF-------
-----------SLTKVNTPLGENLWFDGEFFHS-LTIDNSTRSLFP-------Q-DTPFK
L----PLKRCSVVGNGGILKNSRCGEQIDEADFVMRCNLPPLSREYTEDVGTRTQLVTVN
PSIIDKRYQNLL--WSRKSFVENLRVYQQSYVYMPAFSTKRGTDPSLRVYYTLADFGTN-
QTVLFANPNFLRNVGKFWKSRGIHSKRLSTGLFMVSAALSLCEEVTIYGFWPFQMDLGG-
RYISHHYYDNTLPLS-----GVHAMPEEFLQLWLLHKSGVLQMQLDQCKKDVSSQKPH--

#Anocar_GT29_ST8Sia1
---------GEKEIM--CGMRLQQKCKAWQRGHHAAHAFRKLLKECCDPGQLF-------
-----------AMTKENSPVGKNLWYDGEFLYS-FTIDNETFSLFP-------K-ATPFQ
L----PLKKCSVVGNGGILKKSNCGRQIDQADFVMRCNLPPLTSEYSKDVGSKTQLVTAN
PSIIKKRFQNLL--WSRKAFVDSVKVYNQSYIYMPAFSMKAGTEPSLRVYYTLADVGAR-
QTVIFANPNFLRDIGKFWKSKGIHGKRLSTGLFLVSAALGMCEEVTIYGFWPFSMDLHG-
NFISHHYYDNILPDS-----GFHAMPEEFLQLWFLHKSGVLRMQLEHCEDSIFHPAS---

#Galgal_GT29_ST8Sia1
---------DEKEIV--QGVLMQQ-GKAWRRNHTAVAVFRKLLEDCCDPGQLF-------
-----------AMTKQNSPMGKNLWFDGEFLYS-FTIDNTTYSLFP-------Q-ATPLQ
L----PLKKCSVVGNGGILKKSGCGKQIDQADFVMRCNLPPLSREYSKDVGLKTQLVTAN
PSIIQKRFQNLL--WSRKAFVDSVKVYNHSYIYMPAFSMKTGTGPSLRVYYTLKDFGAK-
QAVLFANPNFLRDIGKFWKNKGIHAKRLSTGLFLVSAALGLCEEVTIYGFWPFSVDLHG-
KFISHHYYDNVLPDS-----GFHAMPEEFLQLWFLHKSGVLRMQLEQCEDPLTQSSA---

#Bostau_GT29_ST8Sia1
---------DEKEIV--QGVL-QQ-GTAWRRNRTAAGIFRKQMEDCCDPAHLF-------
-----------AMTKMNAPMGKSLWYDGEFLYS-FTIDNSTYSLFP-------Q-ATPFQ
L----PLKKCAVVGNGGILKKSGCGRQIDEADFVMRCNLPPLSSEYTKDVGSKSHLVTAN
PSIIRQRFQNLL--WSRKTFVDHMKVYNHSYIYMPAFSMKTGTEPSLRVYYTLSDVGAN-
QTVLFANPNFLRSIGKFWKSRGIHAKRLSTGLFLVSAALGLCEEVAIYGFWPFSVNMHE-
QPISHHYYDNVLPFS-----GFHAMPEEFLQLWYLHKIGALRMQLDPCEDNSL-QPTS--

#Canlupfam_GT29_ST8Sia1
---------NEKEIV--QGVL-QQ-GTAWRRNQTAARVFRKQMEDCCDPAHLF-------
-----------AMTKLNSPMGKSMWYDGEFLYS-FTIDNSTYSLFP-------Q-ATPFQ
```

L----PLKKCAVVGNGGILKKSGCGRQIDEANFVMRCNLPPLSSEYTKDVGSKSHLVTAN
PSIIRQRFQNLL--WSRKTFVDNMKIYNHSYIYMPAFSMKTGTEPSLRVYYTLSDVGAN-
QTVLFANPNFLRSIGKFWKSRGIHAKRLSTGLFLVSAALGLCEEVAIYGFWPFSVNMHE-
QPISHHYYDNVLPFS-----GFHAMPEEFLQLWYLHKIGALRMQLDPCEDTSL-QPTS--

#Pantro_GT29_ST8Sia1
---------NEKEIV--QGVL-QQ-GTAWRRNQTAARAFRKQMEDCCDPAHLF-------
-----------AMTKMNSPMGKSMWYDGEFLYS-FTIDNSTYSLFP-------Q-ATPFQ
L----PLKKCAVVGNGGILKKSGCGRQIDEANFVMRCNLPPLSSEYTKDVGSKSQLVTAN
PSIIRQRFQNLL--WSRKTFVDNMKIYNHSYIYMPAFSMKTGTEPSLRVYYTLSDVGAN-
QTVLFANPNFLRSIGKFWKSRGIHAKRLSTGLFLVSAALGLCEEVAIYGFWPFSVNMHE-
QPISHHYYDNVLPFS-----GFHAMPEEFLQLWYLHKIGALRMQLERCEDTSL-QPTS--

#Homsap_GT29_ST8Sia1
---------NEKEIV--QGVL-QQ-GTAWRRNQTAARAFRKQMEDCCDPAHLF-------
-----------AMTKMNSPMGKSMWYDGEFLYS-FTIDNSTYSLFP-------Q-ATPFQ
L----PLKKCAVVGNGGILKKSGCGRQIDEANFVMRCNLPPLSSEYTKDVGSKSQLVTAN
PSIIRQRFQNLL--WSRKTFVDNMKIYNHSYIYMPAFSMKTGTEPSLRVYYTLSDVGAN-
QTVLFANPNFLRSIGKFWKSRGIHAKRLSTGLFLVSAALGLCEEVAIYGFWPFSVNMHE-
QPISHHYYDNVLPFS-----GFHAMPEEFLQLWYLHKIGALRMQLDPCEDTSL-QPTS--

#Macmul_GT29_ST8Sia1
---------NEKEIV--QGVL-QQ-GTAWRRNQTAARAFRKQMEDCCDPAHLF-------
-----------AMTKMNSPMGKSMWYDGEFLYS-FTIDNSTYSLFP-------Q-ATPFQ
L----PLKKCAVVGNGGILKKSGCGRQIDEANFVMRCNLPPLSSEYTKDVGSKSHLVTAN
PSIIRQRFQNLL--WSRKTFVDNMKIYNHSYIYMPAFSMKTGTEPSLRVYYTLSDVGAN-
QTVLFANPNFLRSIGKFWKSRGIHAKRLSTGLFLVSAALGLCEEVAIYGFWPFSVNMHE-
QPISHHYYDNVLPFS-----GFHAMPEEFLQLWYLHKIGALRMQLDPCEDTSL-QPTS--

#Musmus_GT29_ST8Sia1
---------NEKEIV--QGVL-AQ-STAWRTNQTSASLFRRQMEDCCDPAHLF-------
-----------AMTKMNSPMGKSLWYDGELLYS-FTIDNSTYSLFP-------Q-ATPFQ
L----PLKKCAVVGNGGILKMSGCGRQIDEANFVMRCNLPPLSSEYTRDVGSKTQLVTAN
PSIIRQRFENLL--WSRKKFVDNMKIYNHSYIYMPAFSMKTGTEPSLRVYYTLKDVGAN-
QTVLFANPNFLRNIGKFWKSRGIHAKRLSTGLFLVSAALGLCEEVSIYGSWPFSVNMQG-
DPISHHYYDNVLPFT-----GYHAMPEEFLQLWYLHKIGALRMQLDPCEEP-SPQPTS--

#Ratnor_GT29_ST8Sia1
---------NEKEIV--QGVL-AQ-RTAWRRNQTSARLFRKQMEDCCNPAHLF-------
-----------AMTKVNSPMGKSLWYDGEFLYS-LTIDTSTYSLFP-------Q-ATPFQ
L----PLKKCAVVGNGGILKMSGCGRQIDEANFVMRCNLPPLSSEYTRDVGSKTQLVTAN
PSIIRQRFENLL--WSRKKFVDNMKIYNHSYIYMPAFSMKTGTEPSLRVYYTLKDAGAN-
QTVLFANPNFLRNIGKFWKGRGIHAKRLSTGLFLVSAALGLCEEVSIYGFWPFSVNMQG-
EPISHHYYDNVLPFS-----GFHAMPEEFLQLWYLHKMGALRMQLDPCENPPSPQPTS--

#Calmil_GT29_ST8Sia6
ASASNQWR-SEEDV---LRNIAKIHQCKWGKQKRAVENFRWELRKCCRTLSGS-------
-----------FVTQRNTPVGTELFYEAEPKKK-IKITPSIFAIFP-------K-DSPFR
GR---SIQRCAVVGNAGILHNSSCGAEIDQADFVFRCNLPPMGGNFTKDVGSKTHLVTAN
PKVIIERYAELH--KRRKPFANTLVIYNDALLLFPAFYHSKNTALSFRAHYTLQDFKSK-
QRVIFFNPIYLKHLAHFWLSKGMQVQNLSTGITVASMAMELCSEVWIYGFWPFGKNTEG-
ELMSHHYFDSLLAEP-----DLHSTSNEFYQLLRMHSKGIVKLQMGQCEAEETDEFTLKP

#Hetzeb_GT29_ST8Sia6
ASSPIQWK-TEKDV---IKSVAKLQKCQWHKQEVAAEELRSELKRCCHAAYNY-------
-----------LVTQKNMPVGTELIYDAESKNK-INITPQIFSIFP-------K-NSFFS
GR---QYRRCAVIGNGGILANSSCGAEIDQADFVFRCNLPPMGGEFTRDIGTKTNLVTAN
PSIILERYAKLQ--DRRRSFFTNLAVYDEALLLLPAFSYSKNTDLCFRALYTLQDFQAK-
QKVVFFNPVYLKNLANFWLSKGIQVKRLSTGIMIVSVAVELCEEVWIYGFWPFGKNVEG-
KVVSHHYFDNLLPKP-----DTHSMPLEFHHLLQMHSKGMIKLQMTQCQADPK-------

```
#Squaca_GT29_ST8Sia6
----------EEEV---IKTVAQLQQCQWHKQELEAEKFRSELKRCCYAAYDG-------
-----------LVTQRNTPVGTELTYDAEPKKK-IKITHPIFTIFP-------K-DSAFS
SR---QYRRCAVIGNGGILANSSCGAEIDQADFVFRCNLPPVGGDFAQDVGTKTNLVTAN
PSIIAERYAELH--DRRKPFFTNLTVYNEALLLLPAFSYTKNTVLCFRALYTLQDFQAK-
QKVVFFNPVYLKHLTEFWLSKGIQVNRLSTGIMVVSVAIELCEEVWIYGFWPFGRNVEG-
RVMSHHYFDNLLPKP-----DVHSMPSEFHQLLQMHSKGIIKLQTAQCQADPKEMGNI--

#Calmil_GT29_ST8Sia5
TEVKVLSMVKQSEL---FERWRTLQLCKWELNKTEANT--ARLTNCCNAIQNF-------
-----------TVTQVNTALGANLTYDAQPKKQ-ITITEDIFNLLP-------Q-EMPYS
RS---QFKKCAVVGNGGILKKSECGKEINSADFVFSCNLPPLSNEYARDVGHRTQLVTAN
PSIIKKRFQKLD--KWRRPFVELLQVYENTSVVMPAFYNTRNTDVSLRVRYALDDFSAP-
QDLFYFHPRYLENVARFWGRQGVRAKRPSSGLMLVTAAMELCQEVHLYGFWGFPMDPSG-
IHITHHYYDNVKPRP-----GFHSMPNEIFTFLHMHSRGVLQVHTTPCR-----------

#Danrer_GT29_ST8Sia5
TDVKVLTMVKTSEL---FERWRNLQVCKWDQNKEETDNFKMSLSRCCNAPSFL-------
-----------FTTKRNTPSGTKLRYEVDTSGI-LHISPEIFKMFP-------D-DMPFS
KS---QFKKCAVIGNGGIIKNSKCGREIDASDFVFRCNIPPVSDLYSQDVGSKTDLVTIN
PSIITERFQKLE--KWRKPFYEVLQNYENSSVVLPAFYNTRNTDVSFRVKYMLDDFESS-
RGVFFFHPQYLLNVQRFWAVQGVRAKRLSSGLMLVTAAMELCEEVHLYGFWAFPMNPSG-
IFITHHYYDNVKPRP-----GFHAMPYEIFNFMHMHARGIVHVHTGPCR-----------

#Gasacu_GT29_ST8Sia5
MDVKVLTMVKTSDL---FERWRNLQVCRWEQNKEETSNFKMSLTRCCNAPSFL-------
-----------FTTKKNTPAGTKLRYEVDTSGI-LPITTEVFKMFP-------D-DMPYS
KS---QYKKCAVVGNGGIIKSTKCGKEIDSADFVFRCNLPPLDNGYENDVGIKTDLVTAN
PSILVCRFQKLE--KWRRPFYEVLQKYENSSVVLPAFYNTRNTDVSFRVKYMLDDFDSQ-
RGVFFFHPQYLLNVQRFWAVQGVRAKRVSSGLMLVTAALEMCEEVHLYGFWAFPMNPSG-
IYITHHYYDNVKPRP-----GFHAMPHEIFNFIHMHTRGIVNVHTGQCT-----------

#Orylat_GT29_ST8Sia5
MDVKVLTMVKTSDL---FERWRNLQVCKWEQSKEETSNFKLSLSRCCNAPSFL-------
-----------FTTKRNTPAGSKLRYEVDTSGI-LPITNEVFKMFP-------H-DMPYS
KS---QFKKCAVIGNGGIIKNSKCGKEIDSADFVFRCNIPPIKEKYSTDVGSKTDLVTIN
PSIITERFQKLE--KWRRPFYDVLQNYENSSVVLPAFYNTRNTDVSFRVKYMLDDFDSQ-
RSVFFFHPQYLLNVQRFWAVQGVRAKRLSSGLMLVTAALEICEEVHLYGFWAFPMNPSG-
VYITHHYYDNVKPRP-----GFHAMPHEIFNFIHMHTRGIINVHTDQCM-----------

#Takrub_GT29_ST8Sia5
MDVKVLTMVKTSDL---FERWRNLQICRWEQNKEETSNFKMSLSRCCNAPSFL-------
-----------FTTKRNTPAGTKLRYEVDTSGI-LPITAEVFKMFP-------D-DMPYS
KS---QFKKCAVVGNGGIIKNSKCGKEIDSADFVFRCNIPPISEKYSADVGTKTDLVSIN
PSIITERFQKLE--KWRRPFYEVLQNYENSSAVLPAFYNTRNTDVSFRVKYMLDDFDSQ-
RGVFFFHPQYLLNVQRFWAVQGVRAKRLSSGLMLVTAALEMCEEVHLYGFWAFPMNPSG-
IFITHHYYDNVKPRP-----GFHAMPHEIFNFIHMHTRGIVNVHTGQCT-----------

#Tetnig_GT29_ST8Sia5
MDVKVLTMVKTSDL---FERWRNLQVCKWEQNKEETSNFKMSLSRCCNAPSFL-------
-----------FTTRRNTPAGTKLRYEVDTSGI-LPITAEIFKMFP-------D-DMPYA
KS---QFKKCAVVGNGGIIKNSKCGTEIDSADFVFRCNIPPISEKYSADVGTKTDLVSIN
PSIITERFQKLE--KWRRPFYEVLQNYENSSVVLPAFYNTRNTDVSFRVKYMLDDFDSQ-
RGVFFFHPQYLLNVQRFWAVQGVRAKRLSSGLMLVTAALEMCEEVHLYGFWAFPMNPSG-
IFITHHYYDNVKPRP-----GFHAMPHEIFNFIHMHTRGIVNVHTGRCV-----------

#Siltro_GT29_ST8Sia5
TNVKVLSMVKESDL---FDRWKSLQVCKWEMNVTEANAFKSALTRCCNAPSFL-------
-----------FTTQKNTPLGTKLRYEVDTSGI-FPISSEIFNIFP-------KQDMPYY
RS---QFKKCAVVGNGGILKDSKCGKDIDSTDFVFRCNLPPITPKYVEDVGMKTDVVTIN
PSIITERFNKLE--KWRRPFYEVLQGYENASLLLPAFYNTRNTDVSIRVKYVLDDFESQ-
```

QAVYYFHPQYLINVSRFWLMQGVHAKRISSGLILVTAALELCEEVHLYGFWGFPMDPSG-
NFITHHYYDNVKPRP-----GFHAMPSEIFNFIHMHSKGILRVHTGTC------------

#Musmus_GT29_ST8Sia5
LEVKVLSMVKQSEL---FERWKSLQICKWAMGASEASLFKSTLSRCCNAPNFL-------
-----------FTTQKNTPVETNLRYEVESSGL-YHIDQEIFKMFP-------K-EMPYY
RS---QFKKCAVVGNGGILKNSGCGKEINSADFVFRCNLPPISGIYTTDVGEKTDVVTVN
PSIIIDRFHKLE--KWRRPFFSVLQRYENASVLLPAFYNVRNTLVSFRVKYMLDDFQSR-
QPVYFFHPQYLSSVSRYWLSLGVRARRISTGLSLVTAALELCEEVHLFGFWAFPMNPSG-
FFITHHYYDNVKPKP-----GFHAMPSEIFTFLRMHSRGILRVHTGTCNCC---------

#Ratnor_GT29_ST8Sia5
LEVKVLSMVKQSEL---FERWKSLQICKWAMDASEASLFKSTLSRCCNAPNFL-------
-----------FTTQKNTPVETNLRYEVESSGL-YHIDQEIFKMFP-------K-EMPYY
RS---QFKKCAVVGNGGILKNSGCGKEINSADFVFRCNLPPISGIYTTDVGEKTDVVTVN
PSIIIDRFHKLE--KWRRPFFSVLQRYENASVLLPAFYNVRNTLVSFRVKYMLDDFQSG-
EPVYFFHPHYLSSVSRYWLSLGVRARRISTGLILVTAALELCEEVHLFGFWAFPMNPSG-
FFITHHYYDNVKPKP-----GFHAMPSEIFTFLRMHSRGILRVHTGTCNCC---------

#Anocar_GT29_ST8Sia5
TDVKVLSMVKQTEL---FERWKNLQMCKWEMNITQANIFKATLLRCCNAPAFL-------
-----------FTTQKNTPLGTKLKYEVDTSGI-FHVNQETFRMFP-------Q-EMPYS
RS---QFKKCAVVGNGGILKNSRCGREIDSADFVFRCNLPPISEKYIADVGVKTDIVTVN
PSIITERFHKLE--KWRKPFFNVLQTYENASVLLPAFYNTRNTDVSIRVRYALDDFESQ-
QSVYFFHPQYLINLSRYWLNQGVRAKRISTGLILVTAALELCEEVHLFGFWAFPMNPSG-
IYITHHYYDNVKPRP-----GFHAMPSEIFNFLHMHSKGILRVHTDACNCC---------

#Galgal_GT29_ST8Sia5
LEVKVLSMVKQTEL---FDRWKSLQMCKWEMNVTEANILKSTLSRCCNAPAFL-------
-----------FTTQKNTPLGTKLKYEVDTSGI-FHINQEIFKMFP-------K-DMPYH
RS---QFKKCAVVGNGGILKNSRCGREIDSADFVFRCNLPPISEKYLTDVGVKTDVVTVN
PSIITERFHKLE--KWRKPFYDVLQVYENASVLLPAFYNTRNTDVSIRVKYVLDDFESQ-
QAVYYFHPQYLINVSRYWLGQGVRAKRISTGLILVTAALELCEEVHLFGFWAFPMNPSG-
IFITHHYYDNVKPRP-----GFHAMPSEIFNFLHMHSKGILRVHTGTCGCC---------

#Bostau_GT29_ST8Sia5
LEVKVLSMVKQSEL---FDRWKSLQMCKWAMNISEANQFKSTLYRCCNAPPFL-------
-----------FTTQKNTPLGTKLKYEVDTSGI-YHINQEIFRMFP-------K-DMPYY
RS---QFKKCAVVGNGGILKNSRCGREINSADFVFRCNLPPISEKYTMDVGVKTDVVTVN
PSIITERFHKLE--KWRRPFYRVLQLYENASVLLPAFYNTRNTDVSIRVKYVLDDFESP-
QAVYYFHPQYLVNVSRYWLSLGVRAKRISTGLILATAALELCEEVHLFGFWAFPMNPSG-
LYITHHYYDNVKPRP-----GFHAMPSEIFNFLHLHSRGILRVHTGTCSCC---------

#Homsap_GT29_ST8Sia5
LEVKVLSMVKQSEL---FDRWKSLQMCKWAMNISEANQFKSTLSRCCNAPAFL-------
-----------FTTQKNTPLGTKLKYEVDTSGI-YHINQEIFRMFP-------K-DMPYY
RS---QFKKCAVVGNGGILKNSRCGREINSADFVFRCNLPPISEKYTMDVGVKTDVVTVN
PSIITERFHKLE--KWRRPFYRVLQVYENASVLLPAFYNTRNTDVSIRVKYVLDDFESP-
QAVYYFHPQYLVNVSRYWLSLGVRAKRISTGLILVTAALELCEEVHLFGFWAFPMNPSG-
LYITHHYYDNVKPRP-----GGHAMPSEIFNFLHLHSRGILRVHTGTCSCC---------

#Pantro_GT29_ST8Sia5
LEVKVLSMVKQSEL---FDRWKSLQMCKWAMNISEANQFKSTLSRCCNAPAFL-------
-----------FTTQKNTPLGTKLKYEVDTSGI-YHINQEIFRMFP-------K-DMPYY
RS---QFKKCAVVGNGGILKNSRCGREINSADFVFRCNLPPISEKYTMDVGVKTDVVTVN
PSIITERFHKLE--KWRRPFYRVLQVYENASVLLPAFYNTRNTDVSIRVKYVLDDFESP-
QAVYYFHPQYLVNVSRYWLSLGVRAKRISTGLILVTAALELCEEVHLFGFWAFPMNPSG-
LYITHHYYDNVKPRP-----GFHAMPSEIFNFLHLHSRGILRVHTGTCSCC---------

#Macmul_GT29_ST8Sia5
LEVKVLSMVKQSEL---FDRWKSLQMCKWAMNISEANQFKSTLSRCCNAPAFL-------

-----------FTTQKNTPLGTKLKYEVDTSGI-YHINQEIFRMFP-------K-DMPYY
RS---QFKKCAVVGNGGILKNSRCGREINSADFVFRCNLPPISEKYTMDVGVKTDVVTVN
PSIITERFHKLE--KWRRPFYRVLQVYENASVLLPAFYNTRNTDVSIRVKYVLDDFESP-
QAVYYFHPQYLVNVSRYWLSLGVRAKRISTGLILATAALELCEEVHLFGFWAFPMNPSG-
LYITHHYYDNVKPRP-----GFHAMPSEIFNFLHLHSRGILRVHTGTCSCC---------

#Ambmex_GT29_ST8Sia7
ATLSAHKKIGEKSI---MRNAAELQRCPWQENREKKEFYRSEINQCCNSSHGL-------
-----------IVTQENTRVGENIVYETQKSVK-INVTTEIFNMFP-------K-ESPFS
GK---AYKTCAVVGNGAILVDSCCGQQIDQAEFVFRFNLPPLN--YTKDSGKKVDLVTAN
PSIMINRFQSLN--HRRKAFVDMLRAYRGALILMPTFSHTFGTQLAFKVQYTLDDFGLG-
SRMLSFHPVYLENLAKYWRSKGLYVKRLSSGIMLVSAAMELCDSITLYGFWPFYSNLEG-
RTIGYHYYDKMAPNI-----QVHAMPKEFLIYSQMHAQGALKLQVGQCS-----------

#Ambmex_GT29_ST8Sia7-B
ATLSANKTINERSI---MRNAVELQRCPWLENRKKKEFYRSEINQCCKTSHEL-------
-----------ILTQENTQVGEDIVYETQKSKK-INVTTKIFNMFP-------K-ESPFS
GK---AYKTCAVVGNGAILVDSCCGQQIDQAEFVFRFNLPPLN--YTRDAGRRVDLVTAN
PSIMINRFQSLN--QRRKAFGNTLREYRGALILMAAFSHTRSTQVAFKVQYTLDDFGLG-
SRMLSFHPVYLENLAKYWRSKGLYVKRLSSGIMLVSAAMELCDSITLYGFWPFYSNLEG-
RTIGYHYYDKMAPNI-----QVHAMPKEFLIYSQMHAQGALKLQVGQCS-----------

#Latcha_GT29_ST8Sia7
TS---------------------------------QELPKETDLNKCSNLTQRL-------
-----------VLTRENAPIGHEIEYEVAE-QR-VRVEEPLFKLLPRFPPPFYQ-ESPFK
KA---PYKRCAVVGNAGILLNSGCGRRIDQADFVFRCNLPPLN--YSKDVGSKTDLVTAN
PSIIIQKYHGLK--ESRRLFVERMKVYKDALILMAAFSQSFATEISLAVAYALEDFGSK-
QKAIFFHPAYLRQLGSLWRSWGVRARRLSSGLMLVSAALELCDSVALYGFWPFSTGLDG-
EAVLHHYYDNVPPEP-----GVHAMSSEFVHYLQMHSKGVLQLHVGKC------------

#Rancat_GT29_ST8Sia7
LNSVLRIFKQQAF----FKVVKTLQGCPWKEDQTERNLLQANLGKCCNASYSM-------
-----------IMTQENTVIGQTITFDAETKIK-QNITKSLYSLFP-------K-KSPFQ
K----PIRTCAVVGNGGILTNSSCGAQIDGADFVFRLNFPPLN--WTDDIGAKTDLVTSN
PSILMNKFSSLT--EKRKPFIMMVQEYSSPLILLPAFSYSANTEVSLRVLYTIEDFELN-
SKVVFFNPEYLKNLSAYWKSMGLKFGRLSSGLMVVSIAMEVCDKVTLFGFWPFSKDLNG-
VPILHHYYDNVPPKP-----GIHAMPDEFYKYLQMHIQGSLRLNLEHC------------

#Pelnig_GT29_ST8Sia7
LNSVMKIFRRKTF----FEVVKALQGCPWREDQTERNLLKAKFRKCCNASYSM-------
-----------MVTQENTPIGHIIHYDGDK-GR-KNVTESLYSLFP-------E-KSPFQ
K----PIRSCAVVGNGGILNSSFCGAEIDRANFVFRLNLPPMN--WTNDVGTKTDVVTAN
PSILIDKFGSLM--ERRKPFITRMKEYGSTLIILPAFSYLLNTAVSLRALYTISDFNLN-
SRAVFFNPDYLRNLTAYWKDMGIKSLRLSSGLMLVSAAIEMCDKVTLYGFWPFSQDLDG-
VPIPHHYYDNALPTP-----KIHSMPDEFYQFLQMHIQGSLRLNLGQC------------

#Strcamaus_GT29_ST8Sia7
TESFL-KRVDEVWS---LSHLKLMQSCRWNFNASALAQYRAELGHCCNASAWL-------
-----------ALTQVNTPLGSNIVYDGYRSKS-LKVSSGLLEILP-------E-ESPFQ
EP---FYKTCAVVGNGGILRNSSCGSEIDEHQFVIRFNLPSMD--FPEDVGRKSSIVTVN
PSILQKRFHGLN--GRRLPFVKAAAIYGETWFLIPAFSYPGQNEASYRALYALQDSGSR-
SPIFFFHPQYLSALTRYWHEHGFHPTRLSSGFMLVNAALELCQHITLYGFWPFSLHPDG-
HPLPHHYYDNQLPNP-----RMHLMPQEFACYVNMHFQGVLQLHVGKC------------

#Aptausman_GT29_ST8Sia7
TESFL-KRVNEVWS---LRHLKLMQSCRWNFNASALAQYRAELGHCCNASAWL-------
-----------VLTRVNTPLGTKIVYDGDHSKS-LKVSSGLLEILP-------E-ESPFQ
DP---FYKTCAVVGNGGILLNSSCGSEIDGHQFVIRFNLPSTD--FPEDVGRKSSIVTVN
PSILQERFHGLT--GRRLPFVKAAAFYGETWFLIPAFSYPGQNEASYRALYALQDSGSR-
SPVFFFHPQYLSALSRYWHERGFHTPRLSSGFMLVNAALELCQHITLYGFWPFSLHPDG-
HPLPHHYYDNQLPKP-----RVHLMPQEFAYYVNMHFQGVLHLHLGKC------------

#Cuccancan_GT29_ST8Sia7
---QLGHYCNASAWL-------
-----------AVTQENTPLGSNIVFDGYRSKS-LKVSSGLLEILP-------E-KSPFQ
DL---LYKTCAVVGNGGILRNSSCGSQIDGYQFVIRFNLPSAD--FPEDVGRKSSIVTVN
PSILHKRFHGLN--GRRLPFVEAAASYGKTWFFIPAFSYPGNSEASYRAFYALQDSESQ-
SHVFFFHPQYLSALSKYWHDRGFHTYRLSSGFMLVNAALELCEHITLYGFWPFSLHPDG-
HALPHHYYDNVLPNQ-----RIHIMPREFAYYVDMHFQGVLRLHVGRC------------

#Colliv_GT29_ST8Sia7
TQP-----------------------------------QKAELGRCCNASAWL-------
-----------AVTQDNTPLGSEIVYDAYPSKR-LKVSSGLLEILP-------E-KSPFQ
ES---PYKTCAVVGNGGILRNSSCGSKIDGHEFVIRFNLPSVD--FPEDVGKKSSIVTVN
PSILHKRFHGLN--GRRLPFVKAAASYGKTWFLIPAFSYPSNSEASYRALYALQDSASQ-
SHIFFFHPQYLSSLSKYWHDHGFHTPRLSSGFMLVNAALELCQHITLYGFWPFSLHPDG-
HSLPHHYYDNVLPNS-----RIHIMPKEFTYYVDMHFHGVLQLHLGR-------------

#Apavit_GT29_ST8Sia7
TQP-----------------------------------HKAELGHCCNASAWL-------
-----------AVTQENAPLGSEIVYDGYPSKR-LKVSSGLLEILP-------E-KSPFQ
NP---LYKTCAVVGNGGILRNSSCGAKIDEHQFVIRFNLPSMD--FPEDVGRKSSIVTVN
PSILQKRFHGLN--GRRLPFVKAAAPYGKTWFLIPSFSFPDDTEASYRALYALQDSASE-
SHVFFFHPHYLSALSKYWHDLGFHTYRLSSGFMLVNAALELCQHITLYGFWPFSFHPDG-
HSLPHHYYDNVLPKQ-----NIHIMPKEFSYYVDMHFHGVLQLHLGRC------------

#Chrpicbel_GT29_ST8Sia7
SQRVLPVRLHEAWIWRR---LKLMQSCPWAYNARALGRYREQLGHCCNASADL-------
-----------VLTRDNTPLGSRIVCDGQPAKK-LLVQEALLEILP-------Q-G----
-A---PYDSCAVVGNGGILHNSGCGPEIDRAQFVIRFNLPPMG--FAEDVGTKSSVITVN
PSILVLRFGALS--RWRRPFAEAMGTYGAPLLLIPAFSFISFAAVSSQALYTLEDFGSP-
ARAVFMNPEYLAGLDGHWHRRGLRAKRLSSGFMLVNAALELCQHLTLYGFWPFPTDPEG-
RPLPYHYYDKQTPKP-----GVHAMPDEFTRYLGMHLQGALRLHLGRCQEGLAGGHAQQG

#Malterter_GT29_ST8Sia7
SPI--P----EALVMGAEQCRGLM--QNWSA---GTLPAREQLGSSCNASANL-------
-----------VLTQNNTQLGSQIVYDAQRDKK-HPVKEELLEMLP-------Q-GSPFQ
GA---PYECCAVVGNGGILRNSSCGSEIDRAQFVIRFNLPPMD--FADDVGTKSSVVTMN
PSILHARFRGLS--RWRRPFAEAVGIYGAPLLLIPAFSFVGQSTVSFQALYTLEDFGSP-
ARAVFMNPEYLARLDGHWRPRGLRAKRLSSGFMLVSAALELCQHLTLYGFWPFPTDPEG-
RLLPHHYYDNQPPKP-----GIHAMPDEFTRYLGMHMQGALRLHLGRCQ-----------

#Promuc_GT29_ST8Sia7
-------------RW----QLMLIQECPWKPNATAVMQHRAELGQCCNASHRL-------
-----------VVTKENAPLGSTIHFDGVKVKI-ITVDSKLTNMLL-------E-RFPLA
DA---QYSKCALIGNGGILQDSRCGQEIDQADFIIRFNLPPLN--RTEDVGTKTHLVTIN
PSVLTNRFKNLV--GPPMAFIDAVRAYPNALFLIPALSFHDHIELGYRALHILKDIGLP-
HQAFFLNPHYLGALDMYWKQKGMTEIRLSTGFMFTSFALEFCDHITLYGFWPFLFDLTG-
KPISHHYYDNVLPNA-----FVHSMSEEFSRYIDMYAQGVLRIQLGKCQ-----------

#Deiacu_GT29_ST8Sia7
VI-----ILLPAPLN----GVLLKKSSKWTPTSANLFCSRAL--DCCVCGFHLGFSLSCF
SFEDSKTWCVLRCATGERTAGIHPPFDGVKVKI-ITVDSKLTNMLL-------E-RFPLA
DA---QYSKCALIGNGGILQDSRCGQEIDQADFIIRFNLPPLN--RTEDVGTKTHLVTIN
PSILTNRFKSLV--RSPVAFIDAVRAYPNALFLIPALSFHDHIELGYRALHILKDIGLP-
HQAFFLNPHYLGALDTYWKQKGMTEIRLSTGFMFTSFALEFCDHITLYGFWPFLFDLTG-
KPINHHYYDNVLPHP-----FVHSMSEEFSRYIDMYAQGVLRIQLGKC------------

#Pytmol_GT29_ST8Sia7
VI-----ILLPAPLN----GVLLKKSSKWTPTSANLFCSRAL--DCCVCGFHLGFSLSCF
SFEDSKTWCVLRCATGERTAGIHPPFDGVKVKI-ITVDSKLTNMLL-------E-RSPLA
GF---QYNKCALIGNGGILRHSSCGQEIDQADLIIRFNLPPMN--YTEDVGTKTSLVTIN

PSILNNKFQSLQ--GPQKPFLDALQAYRDALFLIPSLSFSSHHVLGCRAVSIMKDSGLT-
HRAFFLHPHYLGAIRKYWEQKGLKEIRLSTGFLFISIALEFCEHITLYGFWPFSYDLTG-
EPLSHHYYDNILPNA-----GFHTMSEEFLHYLNMYAQGVLRIQLGKC------------

#Anocar_GT29_ST8Sia7-A
--TQLQQCCNASFRF-------
-----------VTTKENIRLGSYIIFDGNPTRK-LRVDTKLLDLLP-------E-KSPFM
DT---SYRKCAVVGNGGILLNSSCGQEIDRADLVIRFNLPPMN--FSEDIGTKTSLVTIN
PSILQNRFKLLQ--ERRKPFVEALHSYSDATFLLPVLSFVGHNILGYRVLYTLEDFGVE-
QQAFFLNPQYLSNLANFWKKRGLKTNRLSSGFMLVSMALEFCQHITLYGFWPFSYDLNN-
QSIPHHYYDNMMPTP-----GVHAMPTEFSYYLSMYAEGVLRLRVGKCQ-----------

#Gekjap_GT29_ST8Sia7
LQ-----KLDEEILA----HLLLTQGCPWQASARAMAQYRTELGRCCNASFWL-------
-----------AITKENTPLGSDILLDGNKGKK-LPVGAELMDLLP-------E-RSPIP
GI---LYDQCAVVGNGGILQNSSCGQEIDQADLIIRFNLPPMN--YSEDVGTKTSLVTIN
PSILQTKFNKLE--AHRKPFADALRPYRSALVFIPAFSFVGHSELAYRALYTMEDFGTG-
QRAYFWNPHYLDTLGIYWKARGFYPHRLSSGFMLVNMALEFCKRITLYGFWPFSHDPAG-
RPIPHHYYDNTWPKP-----GVHAMSQEFSHYLHMYAQGVLQLRLGSCQ-----------

#Amblat_GT29_ST8Sia6
LSATL-RKRYSEDFY--IQSFRKVQNCTWTRRPEEYYKFRSKLASCCNAVNNF-------
-----------IVSQNNTSLGSNMSYEVDNKKN-IVISETIFKMLP-------Q-SQPFV
ER---SYKHCAVVGNGGILQNSSCGAEIDESDFVFRCNLPPVNGDFHKDVGNKTNVVTVN
PSIIALRYGKLS--QKKTVFLQNITKYGDAYFLLPAFSYRSNTAVCFKVFNALKEAKAN-
QKAIFFHPKYLKNLGQFWRANGVRAYRLSTGLMIASAAIELCDHVKLYGFWPFSKNTEG-
NLISHHYYDNQLPKP-----GFHAMPKEFIQYLQLHNKGILKLQVGECENKTKI------

#Ambmex_GT29_ST8Sia6
LSATL-RKRYSEDFY--IQSFRKVQNCTWTRRPEEYYKFRSKLASCCNAVNNF-------
-----------IVSQNNTSLGSNMSYEVDTKKN-IVISETIFKMLP-------Q-SQPFV
ER---SYKHCAVVGNGGILQNSSCGAEIDESDFVFRCNLPPVNGDFHKDVGNKTNVVTVN
PSIIALRYGKLS--QKKTVFLQNITKYGDAYFLLPAFSYRSNTAVCFKVFNALKEAKAN-
QKAIFFHPKYLKNLGQFWRANGVRAYRLSTGLMIASAAIELCDHVKLYGFWPFSKNTEG-
NLISHHYYDNQLPKP-----GFHAMPKEFIQYLQLHNKGILKLQVGECENKTKI------

#Siltro_GT29_ST8Sia6
LTVSL-KKRYSEDYY--IQTVNDLQNCTWHKRPEERSKFRLDLSSCCNGVKNF-------
-----------IVSQNNTSVGINITYEVESKKR-ILITEEIYRMLP-------K-SQPFD
GT---PFKQCAVVGNGGILANSGCGVEIDQSDFVFRCNLPPTSGNVSVDVGNKTNLVTVN
PSIISQKYRKLN--KVKNVFLKYVSNYGNSLLLLPAFSYSSNTAISFEVHRVLEKNQAK-
QKAVFFHPNYLKNLAKFWKGKGVRAYRLSTGLMITSAAMELCEEVILYGFWPFSKDLEG-
KPISHHYYDNMLPKP-----GFHAMPKEFYQVLQLHHKGVLRLQIGKCEKR---------

#Xenlae_GT29_ST8Sia6
LTVSL-KKRYSEDYY--IQTVNELQNCTWQKRPQEYSKFRLYLSTCCNGLKNF-------
-----------IVSQNNTSLGSNITYEVESKKK-ILIAEEIYRMFP-------K-SQPFV
GA---PFKQCAVVGNGGILANSGCGAEIDQSDFVFRCNLPPTWGNISVDVGNKTNLVTVN
PSIISRKYRKLN--KVKNVFLKNVSNYGYSFLLLPAFSYSSNTAISFEVHRILEKNQAK-
QKAIFFHPYYLKNLAQFWKGRGVRAYRLSTGLMITSAAIELCEEVKLYGFWPFSKNQEG-
KPISHHYYDNILPKP-----GFHAMPKEFYQVLQLHHKGVLRLQIGKCEKR---------

#Litcat_GT29_ST8Sia6
LSVSL-RKRYSEDYY--IQAVNDVQNCTWKKKPQEYSRFRLYLSSCCNAVNNF-------
-----------IVSQNNTSLGSNITYEVESKKN-ILIAEDIYKMLP-------K-SQPFD
GI---PFKQCAVVGNGGILKNSSCGAEIDQSDFVFRCNLPPIWGNVSIDVGNKTDLVTVN
PSIIALKYGKLN--DMKTAFLKNLTNYGYSFLLLPAFSYSSNTAISFEVHHLLRKYQAK-
QKAIFFHPNYLKSLAQFWKGRGVRAYRLSTGFMITSAAIELCQDVTLYGFWPFSKNLDG-
KPISHHYYDNQLPKP-----GFHAMPKEFYQVLQLHHKGVLKLQLGECEKR---------

#Rhimar_GT29_ST8Sia6

```
LTVSL-RKRYSEDCY--IQAVNEVQNCTWQKKPQEYSRFRFYLSSCCNAVNNF-------
-----------IVSQNNTSLGSNITYEVESKKN-ILIAEDIYKMLP-------K-SQPFE
GI---PFKQCAVVGNGGILTNSSCGAEIDRSDFVFRCNLPPIWGNAAVDVGNKTDLVTVN
PSIIALKYGKLN--EMKTAFLRNLTSYGGSFLLLPAFSYSSNTAISFEVHNLLKKYQAK-
QRAIFFHPNYLKSLAQFWRGRGVRAYRLSTGFMITSAAIELCEDVKLYGFWPFSKNPNG-
KPISHHYYDNQLPKP-----GFHAMPKEFYQVLQLHYKGVLKLQIGECQKR---------

#Latcha_GT29_ST8Sia6
MSATTPRKRYSDDYY--IQTVNELQKCSWVIRPEEHEKFRSELSVCCNAVRNF-------
-----------IVSQNNTPLGTNMSYEVENGRT-FLITQRIFKMFP-------Q-SQPFA
GY---PYNQCAVVGNGGILKNSACGTEIDQADFVFRCNLPPTLGNISIDVGSKTSLVTLN
PSIITHRFGKLN--EKRKPFVETVSHYGEAFLLLPAFSFRSNTALSFKVYHTLEAFRGK-
QKTIFFHPKYLKSLALFWRSKGIKVYRLSSGFMIANAAIELCKEVRLYGFWPFSKNNEG-
KNISHHYYDNQLPKP-----GIHSMPKEFYHFLKLHNKGIIKLQFGRCDIT---------

#Latmen_GT29_ST8Sia6
MSATTPRKRYSDDYY--IQTVNELQKCSWVIRPEEHEKFRSELSVCCNAVRNF-------
-----------IVSQNNTPLGTNMSYEVENGRT-FLITQRIFKMFP-------Q-SQPFA
GY---PYNQCAVVGNGGILKNSACGTEIDQADFVFRCNLPPTLGNISIDVGSKTSLVTLN
PSIITHRFGKLN--EKRKPFVETVSHYGEAFLLLPAFSFRSNTALSFKVYHTLEAFRGK-
QKTIFFHPKYLKSLALFWRSKGIKVYRLSSGFMIANAARRLCKEVRLYGFWPFSKNNEG-
KNISHHYYDNQLPKP-----GIHSMPKEFYHFLKLHNKGIIKLQFGRCDIT---------

#Canlupfam_GT29_ST8Sia6
QRLYSKAKNYLESDY--LQIIRNIQNCPWKRQEEEYENFRAKLASCCDAAQNF-------
-----------IVSQNNTPAGTNMSYEVESKNE-ILIRENIFNMFP-------V-SQPFV
EY---PYNQCAVVGNGGILNKSLCGAEIDKSDFVFRCNLPPITGNISKDVGSKTNVVTVN
PSIIRLKYGNLK--KKKEIFLEDIATYGDAFLLLPAFSFRANTIASFKVYSTLKESNAR-
QKVIFFHPKYLRNLALFWRTEGVTEFRLSSGLMITSVAVELCEHVKLYGFWPFSRTVKD-
TPVSHHYYDNNLPKR-----GFHEMPKEYRQILQLHLKGILKLQFSKCEIA---------

#Musmus_GT29_ST8Sia6
NSLSNKTRRYSEDDY--LQTITNIQRCPWNRQAEEYDNFRAKLASCCDAIQDF-------
-----------VVSQNNTPVGTNMSYEVESKKH-IPIRENIFHMFP-------V-SQPFV
DY---PYNQCAVVGNGGILNKSLCGAEIDKSDFVFRCNLPPITGSASKDVGSKTNLVTVN
PSIITLKYQNLK--EKKAQFLEDISTYGDAFLLLPAFSYRANTGISFKVYQTLKESKMR-
QKVLFFHPRYLRHLALFWRTKGVTAYRLSTGLMIASVAVELCENVKLYGFWPFSKTIED-
TPLSHHYYDNMLPKH-----GFHQMPKEYSQMLQLHMRGILKLQFSKCETA---------

#Ratnor_GT29_ST8Sia6
DSLANKTRRYSEDDY--LQIVTNIQRCPWKRQAEEYENFRAKLASCCDAIQDF-------
-----------VVSQNNTPVGSNMTYEVESKKQ-IPIRENIFHMFP-------V-SQPFV
DY---PYNQCAVVGNGGILNKSLCGAEIDKSDFVFRCNLPPITGSASQDVGSKTNLVTVN
PSIITLKYKNLK--EKKARFLEDISAYGDAFLLLPAFSYRVNTGISFKVYQTLKESKVR-
QKVLFFHPRYLRHLALFWRTKGVTAYRLSTGLMIASIAVELCENVTLYGFWPFSKTVEE-
IPLSHHYYDNKLPKH-----GFHQMPKEYSQMLQLHMKGILKLQFSKCEAA---------

#Macmul_GT29_ST8Sia6
ESFSNNTKGYSENDY--LQIITDIQSCPWKRQAEEYANFRAKLASCCDAVQNF-------
-----------VVSQNNTPVGTNMSYEVESKKK-IPIKKNIFHMFP-------V-SQPFV
EY---PYNQCAVVGNGGILNKSLCGAEIDKSDFVFRCNLPPTTGDVSKDVGSKTNLVTIN
PSIITLKYGNLK--EKRALFLEDIATYGDAFFLLPAFSFRANTGTSFKVYYTLEESKAR-
QKVLFFHPKYLKHLALFWRTKGVTAYRLSTGLMITSVAVELCENVKLYGFWPFSKTVED-
IPVSHHYYDNKLPKR-----GFHQMPKEYSQILQLHMKGILKLQFSKCEVA---------

#Ponpyg_GT29_ST8Sia6
ESFSNKTKGYSENDY--LQIITDIQSCPWKRQAEEYANFRAKLASCCDAVQNF-------
-----------VVSQNNTPVGTNMSYEVESKKE-IPIKKNIFHMFP-------V-SQPFV
EY---PYNQCAVVGNGGILNKSLCGTEIDKSDFVFRCNLPPTTGDVSKDVGSKTNLVTIN
PSIITLKYGNLK--EKKALFLEDIATYGDAFFLLPAFSFRANTGTSFKVYYTLEESKAR-
QKVLFFHPKYLKDLALFWRTKGVTAYRLSTGLMITSVAVELCKNVKLYGFWPFSKTVED-
```

```
TPVSHHYYDNKLPKR-----GFHQMPKEYSQILQLHMKGILKLQFSKCEVA---------

#Homsap_GT29_ST8Sia6
ESFSNKTKRYSENDY--LQIITDIQSCPWKRQAEEYANFRAKLASCCDAVQNF-------
-----------VVSQNNTPVGTNMSYEVESKKE-IPIKKNIFHMFP-------V-SQPFV
DY---PYNQCAVVGNGGILNKSLCGTEIDKSDFVFRCNLPPTTGDVSKDVGSKTNLVTIN
PSIITLKYGNLK--EKKALFLEDIATYGDAFFLLPAFSFRANTGTSFKVYYTLEESKAR-
QKVLFFHPKYLKDLALFWRTKGVTAYRLSTGLMITSVAVELCKNVKLYGFWPFSKTVED-
IPVSHHYYDNKLPKH-----GFHQMPKEYSQILQLHMKGILKLQFSKCEVA---------

#Pantro_GT29_ST8Sia6
ESFSNKTKGYSENDY--LQIITDIQSCPWKRQAEEYANFRAKLASCCDAVQNF-------
-----------VVSQNNTPVGTNMSYEVESKKE-IPIKKNIFHMFP-------V-SQPFV
DY---PYNQCAVVGNGGILNKSLCGTEIDKSDFVFRCNLPPTTGDVSKDVGSKTNLVTIN
PSIITLKYGNLK--EKKALFLEDIATYGEAFFLLPAFSFRANTGTSFKVYYTLEESKAR-
QKVLFFHPKYLKDLALFWRTKGVTAYRLSTGLMITSVAVELCKNVKLYGFWPFSKTVED-
IPVSHHYYDNKLPKH-----GFHQMPKEYSQILQLHMKGILKLQFSKCEVA---------

#Bostau_GT29_ST8Sia6
ETLSNRTKGYSEDEY--LQIITNIQSCPWKRQVEEYENFRAKLASCCDAVQNF-------
-----------IVSQNNTPIGTNMTYEVESKSK-IQIKENIFDMLP-------V-VQPFV
GY---SFNQCAVVGNGGILNQSLCGAEIDKADFVFRCNLPPTTGNVTNDVGTKTNLVTLN
PSIIKLRYGNLK--EKKAIFLEDVAAYGDAFVLLPAFSFRANTAASFKVYYTLKESKAR-
QKVLFFHPKYLKNLALFWRTKGVTEYRLSSGLMITSVAVELCENVKLYGFWPFSRTGEN-
MPVSHHYYDNKLPKR-----GFHEMPKEYSQILQLHVKGILKLQFSKCETA---------

#Susscr_GT29_ST8Sia6
KALSNKTRKYSEDDY--LQIITNIQSCPWKRQAEAYENFRAKLASCCDAVQNF-------
-----------IVSQNNTPVGTNMSYEVESKSE-IQIRESIFDMLP-------V-FQPFI
GY---PYNQCAVVGNGGILNQSLCGAEIDKSDFVFRCNLPPTTGNISNDVGSKTNLVTLN
PSIIRLRYGNLK--EKKAVFLEDIATYGDAFVLLPAFSFRANTAASFKVYYALKESNSR-
QKVLFFHPKYLKHLAVFWRTKGVTEYRLSSGLMIASVAVELCENVKLYGFWPFSKTGEN-
TPVSHHYYDNKLPKR-----GFHEMPKEYRQILQLHVKGILQLQFSKCDTS---------

#Gekjap_GT29_ST8Sia6
WAASVKRKRYAEDYY--LQVVGRLQNCTWNKRPQEYAKFKSELASCCDAAHNF-------
-----------ITSQNNTPLGSNMSYEVDNKKT-IHITEEIFRMLP-------E-SQPL-
EQ---PFKNCAVVGNGGILKNSNCGAEIDQSDFVFRCNLPPTMGSIRQDVGSKTNLVTIN
PSIISQKYNKLN--EKKATFLENIASYGDTFLLLPAFSFRSNTAASFKVHHTLKEFSAK-
QKAIFFYPRYLRNLAQFWRTKGVKAYRLSSGFMITSAALELCENVKLYGFWPFSKNVAG-
TPISHHYYDNQLPKP-----GFHAMPKEYNQILQLHGRGILKLQFGKCLPD---------

#Anocar_GT29_ST8Sia6
WSSSIKKKRYSEDYY--LQIVGKLQNCTWKKRPQEYAKFRSELTSCCDAIHNF-------
-----------ISSQNNTPLGSNMSYEVDNKKT-IHITEEIFKMLP-------E-SQPL-
DY---PFKQCAVVGNGGILKNSNCGAEIDKSDFVFRCNLPPTEGSVSQDVGNKTNLVTVN
PSIIAQKYNKLN--EKKATFLENIASYGDAFLLLPAFSFRSNTATSFKVHHTLREFSAK-
QKAIFFYPRYLKNLAQFWRTKGVKAYRLSSGFMITSAAVELCENVKLYGFWPFSKSIAG-
NPISHHYYDNQLPKP-----GFHAMPKEYNQILQLHGRGILKLQLGKCHTE---------

#Tingut_GT29_ST8Sia6
LSSLTKKKRYSEDDF--LHLVTKLQNCTWVKRPEECTKFRSELASCCDAVHNF-------
-----------VASQNNTPLGSNMSYEVESKKT-ILITENIFRMLP-------V-SSPFS
AY---PFKNCAVVGNGGILKNSSCGAEIDRADFVFRCNLPPTTGSMSKDVGNKTNLVTVN
PSIIAQKYNKLN--KKKTEFLQNIAVYGDAFLLLPAFSYRSNTATSFKVYQTLKEFKAM-
QRAIFFHPTYLKSLAQFWRTKGVKAYRLSSGFMITSAAIELCENVKIYGFWPFSKSTEK-
MPISHHYYDNQLPKP-----GFHAMPKEYNQILQLHGKGILKLQFGKCESE---------

#Chemyd_GT29_ST8Sia6
WSSSIKKKRYSEDYY--LQIVTKIQNCTWKRRPEEYTKFRLELASCCDAVHNF-------
-----------IASQNNTALGSNMSYEVDNKKT-LLITEDIFRMLP-------E-SPPLL
```

DY---PFKHCAVVGNGGILKNSSCGAEIDKSDFVFRCNLPPTTGSVSKDVGNKTNLVTVN
PSIIAQKYNKLN--EKKVTFLENIASYGNAFLLLPAFSFKSNTAASFKVYHTLQEFRAK-
QRAIFFHPRYLKSLAQFWRTKGVKAYRLSSGFMIASAAIELCENVKLYGFWPFSKTTEE-
MPISHHYYDNQLPKP-----GFHAMPKEYNQILQLHGKGIVKLQFGKCDSDXKGTSDSSK

#Chrpicbel_GT29_ST8Sia6
WSSSIKKKRYSEDYY--LQIVTKIQNCTWKRRPEEYTKFRLELASCCDAVHNF-------
-----------IASQNNTLLGSNMSYEVDNKKT-ILITEDIFRMLP-------E-SPPFL
GY---PFKHCAVVGNGGVLKNSSCGAEIDNSDFVFRCNLPPTTGSVSKDVGNKTSLVTVN
PSIIAQKYNKLN--EKKVTFLENIASYGNAFLLLPAFSFKSNTAASFKVYHTLQEFRAK-
QRAIFFHPRYLKNLAQFWRTKGVKAYRLSSGFMIASAAIELCENVKLYGFWPFSKTTEE-
MPISHHYYDNQLPKP-----GFHAMPKEYNQILQLHGKGILKLQFGKCDSD---------

#Galgal_GT29_ST8Sia6
LSSSFKKKRYPEDYY--LHIVKKLQNCTWVRRPEESAKFRSELASCCNAVHNF-------
-----------IASQNNTPLGSNMSYEVDSKKT-ILITEDIFRMLP-------V-SSPLS
VY---PFKNCAVVGNGGILKNSSCGAEIDRSDFVFRCNLPPTMGSISKDVGNKTKLVTVN
PSIIAQKYNKLN--EKKTEFVENVAVYGDAFLLLPAFSFRSNTATSFKVYHTLQEFKAT-
QRAIFFHPTYLKNLAQFWRTKGVKAYRLSSGFMITSAAVELCENVKLYGFWPFSKSTEK-
MPISHHYYDNQLPKP-----GFHAMPKEYNQILQLHGKGIKKLQFGKCESD---------

#Chavoc_GT29_ST8Sia6
LSSSIKKKRYSEDYY--LHIVTKLQNCTWTRRPEESTKFRSELASCCDAVHNF-------
-----------IASQNNTPLGSNMSYEVDSKKT-ILITEDIFKMLP-------V-SSPLS
AY---PFKTCAVVGNGGILKNSSCGAEVDRSDFVFRCNLPPTTGSISKDVGNKTNLVTVN
PSIIAQKYNKLN--EKKTEFLENIAVYGDAFLLLPAFSFRSNTATSFKVYHTLQEFKAT-
QRAIFFHPTYLRSLAQFWRTKGVKAYRLSSGFMITSAALELCENVKLYGFWPFSKSTEK-
MPISHHYYDNQLPKP-----GFHAMPKEYNQILQLHGKGILKLQFGKCESD---------

#Egrgar_GT29_ST8Sia6
LSSSIKKKRYSEDYY--LHIVTKLQNCTWIRRPEESMKFRSELASCCDAVHNF-------
-----------IASQNNTPLGSNMSYEVDSKKT-ILITEDIFKMLP-------V-SSPLS
VY---PFKTCAVVGNGGILKNSSCGAEIDRSDFVFRCNLPPTTGSISKDVGNKTNLVTVN
PSIIAQKYNKLN--EKKTEFLEDVAVYGDAFLLLPAFSFRSNTATSFKVYHTLKEFRAS-
QRAIFFHPTYLKSLAQFWRTKGVKAYRLSSGFMITSAALELCENVKLYGFWPFSKSTEK-
MPISHHYYDNQLPKP-----GFHAMPKEYNQILQLHGKGILKLQFGKCESD---------

#Calpug_GT29_ST8Sia6
LSSSIKKKRYSEDYY--LHIVTKLQNCTWIRRPEESTKFRSELASCCDAVHNF-------
-----------IASQNNTPLGSNMSYEVDSKKT-VLITEDIFKMLP-------V-SSPLS
VY---PFKTCAVVGNGGILKNSSCGAEIDRSDFVFRCNLPPTTGSISKDVGNKTNLVTVN
PSIIAQKYNKLN--EKKTEFLDNIAVYGDAFLLLPAFSFRSNTATSFKVYHTLQEFKAT-
QRAIFFHPTYLKSLAQFWRTKGVKAYRLSSGFMITSAALELCENVKLYGFWPFSKSTEK-
MPISHHYYDNQLPKP-----GFHAMPKEYNQILQLHSKGILKVQFGKCESD---------

#Psehum_GT29_ST8Sia6
LSSSIKKKRYSEDYY--LHIVTKLQNCTWIRKPEESTKFRSELASCCDAVHNF-------
-----------IASQNNSPLGSNMSYEVDSKKT-ILITEDIFKMLP-------V-SSPLS
VY---PFKNCAVVGNGGILKNSSCGAEIDSSDFVFRCNLPPTTGNISKDVGNKTNLVTVN
PSIIAQKYNKLN--EKKTEFLENIAAYGDAFLLLPAFSFRSNTATSFKVYHTLKEFKAT-
QRAIFFHPSYLKSLAQFWRTKGVKAYRLSSGFMITSAALELCENVKLYGFWPFSKSIEK-
MPISHHYYDNQLPKP-----GFHAMPKEYNQILQLHGKGVLKLQFGKCESD---------

#Parmaj_GT29_ST8Sia6
LSSSIKKKRYSEDYY--LHIVTKLQNCTWIRKPEESTKFRSELASCCDAVHNF-------
-----------IASQNNSPLGSNMSYEVDSKKT-ILITEDIFKMLP-------V-SSPLS
VY---PFKNCAVVGNGGILKNSSCGAEIDSSDFVFRCNLPPTTGNISKDVGNKTNLVTVN
PSIIAQKYNKLN--EKKTEFLENIAAYGDAFLLLPAFSFRSNTATSFKVYHTLKEFKAT-
QRAIFFHPAYLKSLAQFWRTKGVKAYRLSSGFMITSAALELCENVKLYGFWPFSKSIEK-
MPISHHYYDNQLPKP-----GFHAMPKEYNQILQLHGKGILKLQFGKCESD---------

#Lepcor_GT29_ST8Sia6-B
LSSSIKKKRYSEDYY--LHIVTKLQNCTWIRRPEESTKFRSELASCCDAVHNF-------
-----------IASQNNSPLGSNMSYEVDSKKT-ILITEDIFKMLP-------V-SSPLS
VY---PFKTCAVVGNGGILKNSSCGAEIDSSDFVFRCNLPPTTGSISKDVGNKTNLVTVN
PSIIAQKYNKLN--EKKTEFLENIAVYGDAFLLLPAFSFRSNTATSFKVYHTLKEFKAT-
QRAIFFHPAYLKSLAQFWRTKGVKAYRLSSGFMITSAALELCENVKLYGFWPFSKSTEK-
MPISHHYYDNQLPKP-----GFHAMPKEYNQILQLHGKGILKLQFGKCESD---------

#Lepcor_GT29_ST8Sia6-A
LSSSIKKKRYSEDYY--LHIVTKLQNCTWIRRPEESTKFRSELASCCDAVHNF-------
-----------IASQNNSPLGSNMSYEVDSKKT-ILITEDIFKMLP-------V-SSPLS
VY---PFKTCAVVGNGGILKNSSCGAEIDSSDFVFRCNLPPTTGSISKDVGNKTNLVTVN
PSIIAQKYNKLN--EKKTEFLENIAVYGDAFLLLPAFSFRSNTATSFKVYHTLKEFKAT-
QRAIFFHPAYLKSLAQFWRTKGVKAYRLSSGFMITSAALELCENVKLYGFWPFSKSTEK-
MPISHHYYDNQLPKP-----GFHAMPKEYNQILQLHGKGILKLQFGKCESD---------

#Aquchrcan_GT29_ST8Sia6
LSSSIKKKRYSEDYY--LHIVTKLQNCTWIRRPEESTKFRSELASCCDAVHNF-------
-----------IASQNNSPLGSNMSYEVDSKKT-ILITEDIFKMLP-------V-SSPLS
VY---PFKTCAVVGNGGILKNSSCGAEIDHSDFVFRCNLPPTTGSISKDVGNKTNLVTVN
PSIIAQKYNKLN--EKKTEFLENIAVYGDAFLLLPAFSFRSNTATSFKVYHTLQEFKAT-
QRAIFFHPTYLKSLAQFWRTKGVKAYRLSSGFMITSAALELCENVKLYGFWPFSKSTEK-
MPISHHYYDNQLPKP-----GFHAMPKEYNQILQLHGKGILKLQFGKCESD---------

#Halleuala_GT29_ST8Sia6
LSSSIKKKRYSEDYY--LHIVTKLQNCTWIRRPEESTKFRSELASCCDAVHNF-------
-----------IASQNNSPLGSNMSYEVDSKKT-ILITEDIFKMLP-------V-SSPLS
VY---PFKTCAVVGNGGILKNSSCGAEIDHSDFVFRCNLPPTTGSISKDVGNKTNLVTVN
PSIIAQKYNKLN--EKKTEFLENIAVYGDAFLLLPAFSFRSNTATSFKVYHTLQEFKAT-
QRAIFFHPTYLKSLAQFWRTKGVKAYRLSSGFMITSAALELCENVKLYGFWPFSKSTEK-
MPISHHYYDNQLPKP-----GFHAMPKEYNQILQLHGKGILKLQFGKCESD---------
